# Supplementary figures and images for: SUMOylation of Bonus, the Drosophila homolog of Transcription Intermediary Factor 1, safeguards germline identity by recruiting repressive chromatin complexes to silence tissue-specific genes (part 1 of 2)
Source: eLife. 2023 Nov 24;12:RP89493. doi: 10.7554/eLife.89493 (PMC10672805; doi:10.7554/eLife.89493)

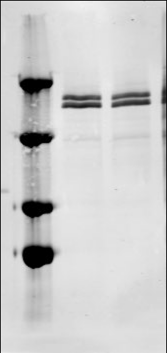

Supplement: Figure 4—source data 1. [file elife-89493-fig4-data1.zip › Figure 4 - source data 1/Raw_image_Figure 4B-Input-SetDB1.tif]

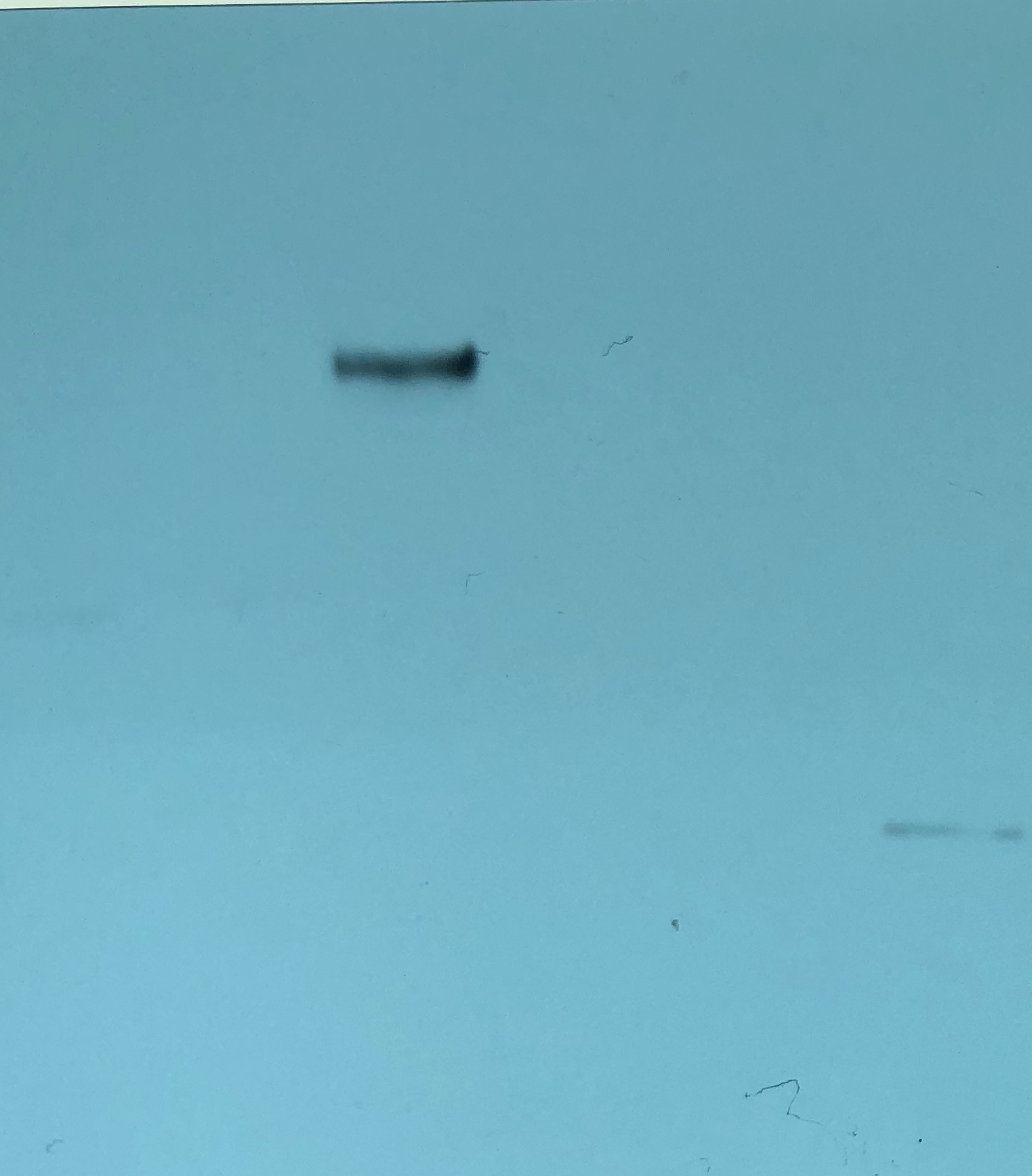

Supplement: Figure 4—source data 1. [file elife-89493-fig4-data1.zip › Figure 4 - source data 1/Raw_image_Figure 4B-IP-SetDB1.tif]

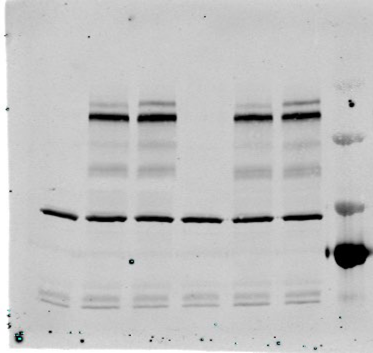

Supplement: Figure 4—source data 1. [file elife-89493-fig4-data1.zip › Figure 4 - source data 1/Raw_image_Figure 4B-Input-Bonus.tif]

Figure 4B

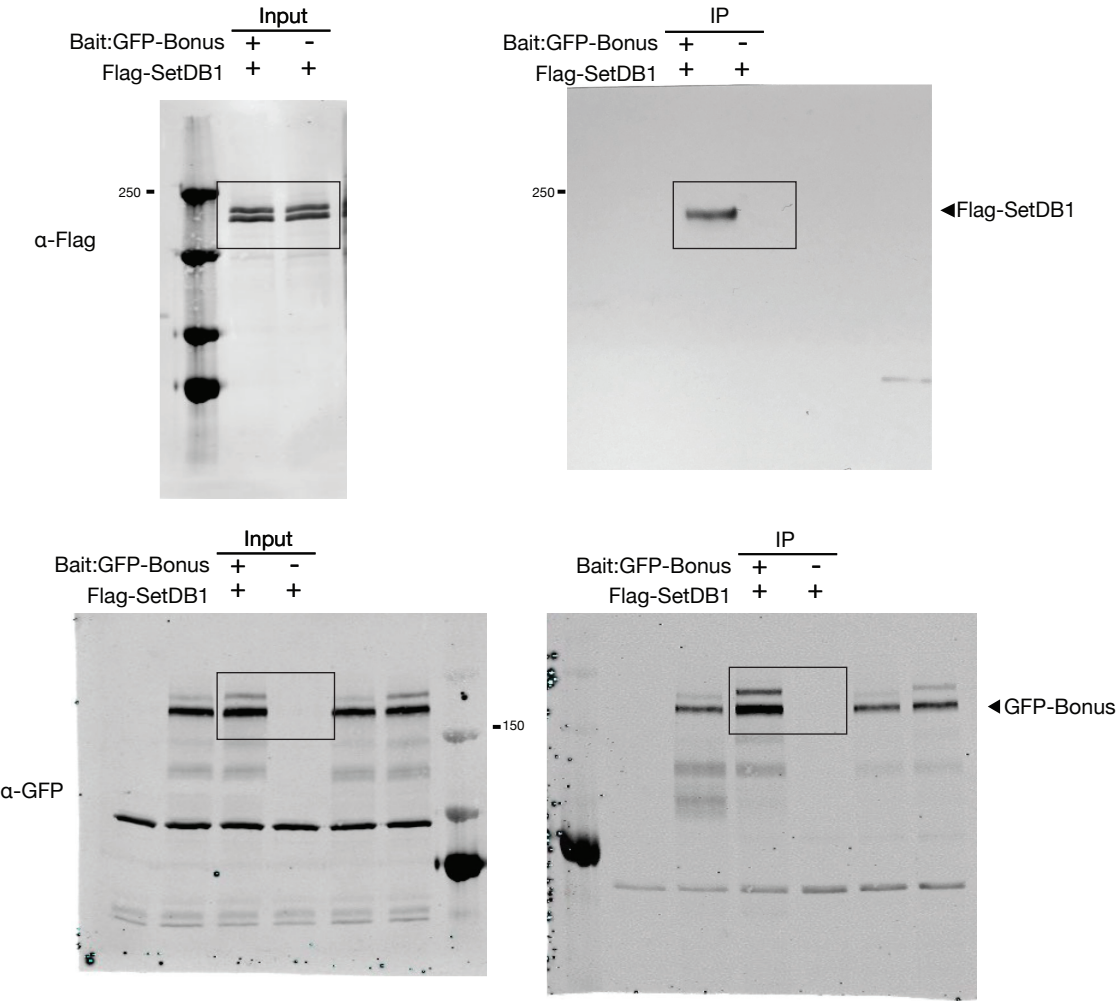

Supplement: Figure 4—source data 1. [file elife-89493-fig4-data1.zip › Figure 4 - source data 1/Figure4B-uncropped blot.pdf]

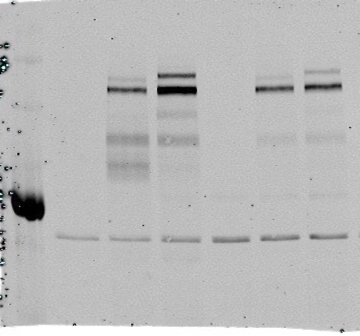

Supplement: Figure 4—source data 1. [file elife-89493-fig4-data1.zip › Figure 4 - source data 1/Raw_image_Figure 4B-IP-Bonus.tif]

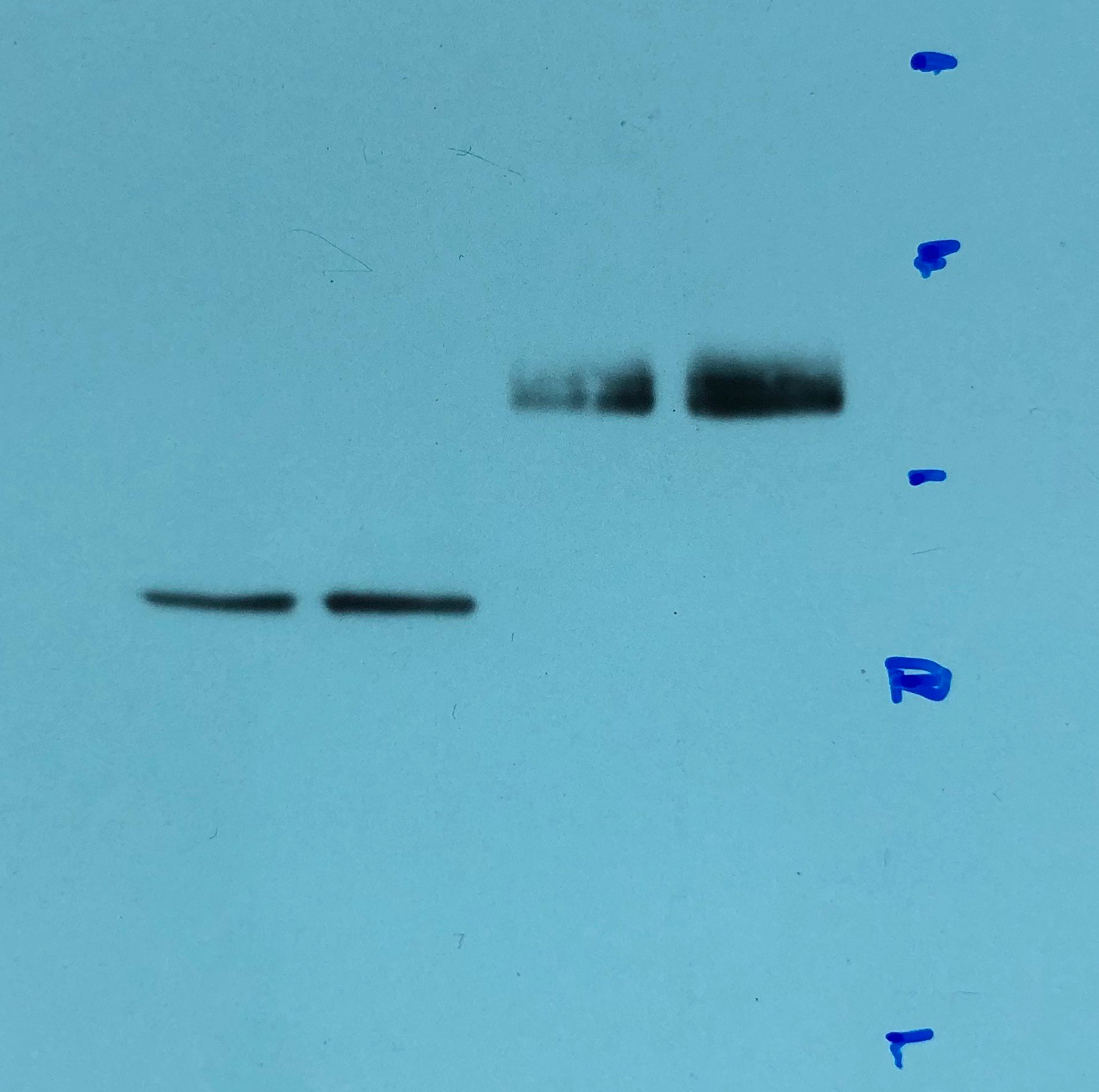

Supplement: Figure 4—source data 2. [file elife-89493-fig4-data2.zip › Figure 4 - source data 2/Raw_image_Figure 4C-Input-Rpd3.tif]

Figure 4C

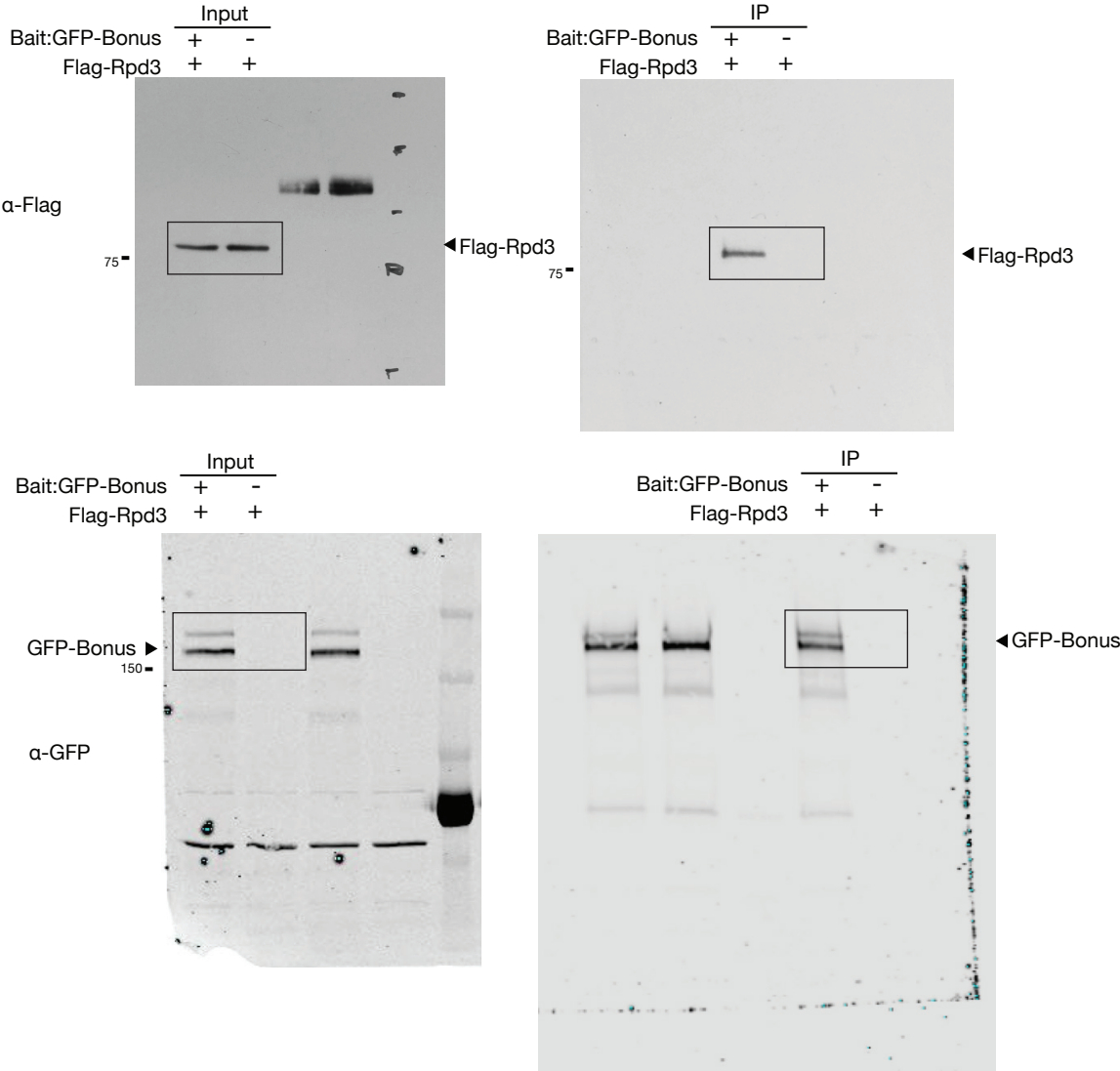

Supplement: Figure 4—source data 2. [file elife-89493-fig4-data2.zip › Figure 4 - source data 2/Figure4C-uncropped blot.pdf]

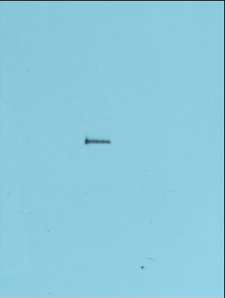

Supplement: Figure 4—source data 2. [file elife-89493-fig4-data2.zip › Figure 4 - source data 2/Raw_image_Figure 4C-IP-Rpd3.tif]

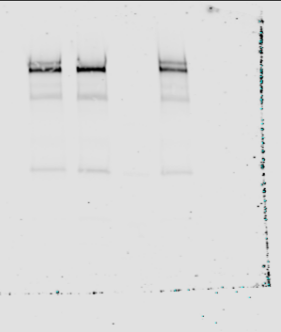

Supplement: Figure 4—source data 2. [file elife-89493-fig4-data2.zip › Figure 4 - source data 2/Raw_image_Figure 4C-IP-Bonus.tif]

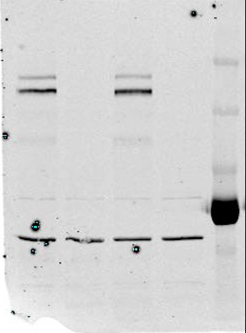

Supplement: Figure 4—source data 2. [file elife-89493-fig4-data2.zip › Figure 4 - source data 2/Raw_image_Figure 4C-Input-Bonus.tif]

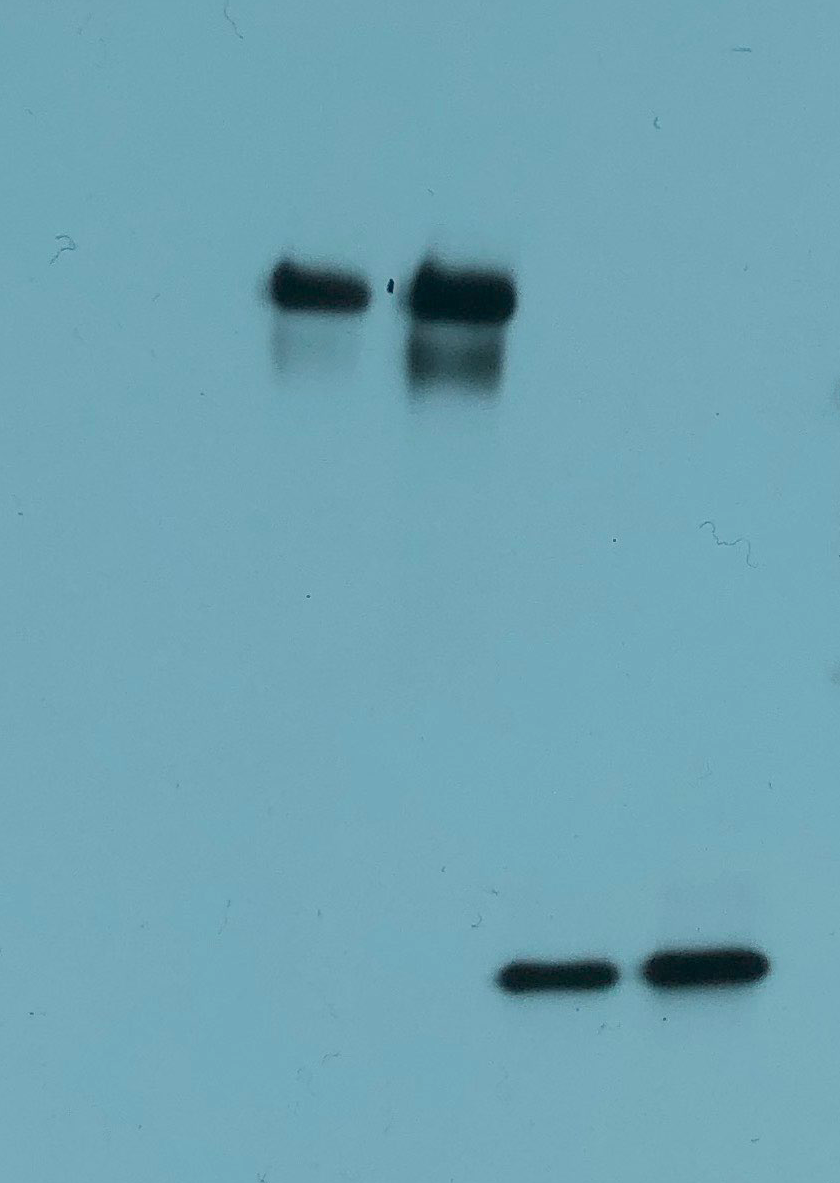

Supplement: Figure 4—source data 3. [file elife-89493-fig4-data3.zip › Figure 4 - source data 3/Raw_image_Figure 4D-Input-Mi2.tif]

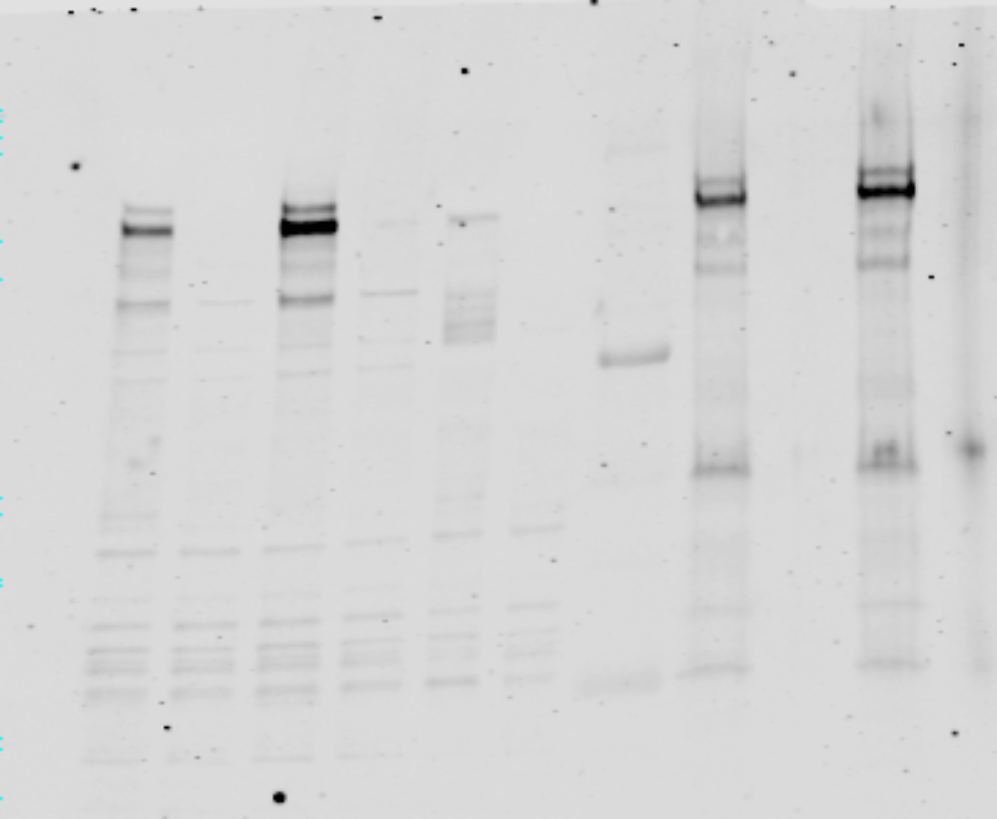

Supplement: Figure 4—source data 3. [file elife-89493-fig4-data3.zip › Figure 4 - source data 3/Raw_image_Figure 4D-Input-Bonus-intensity2.tif]

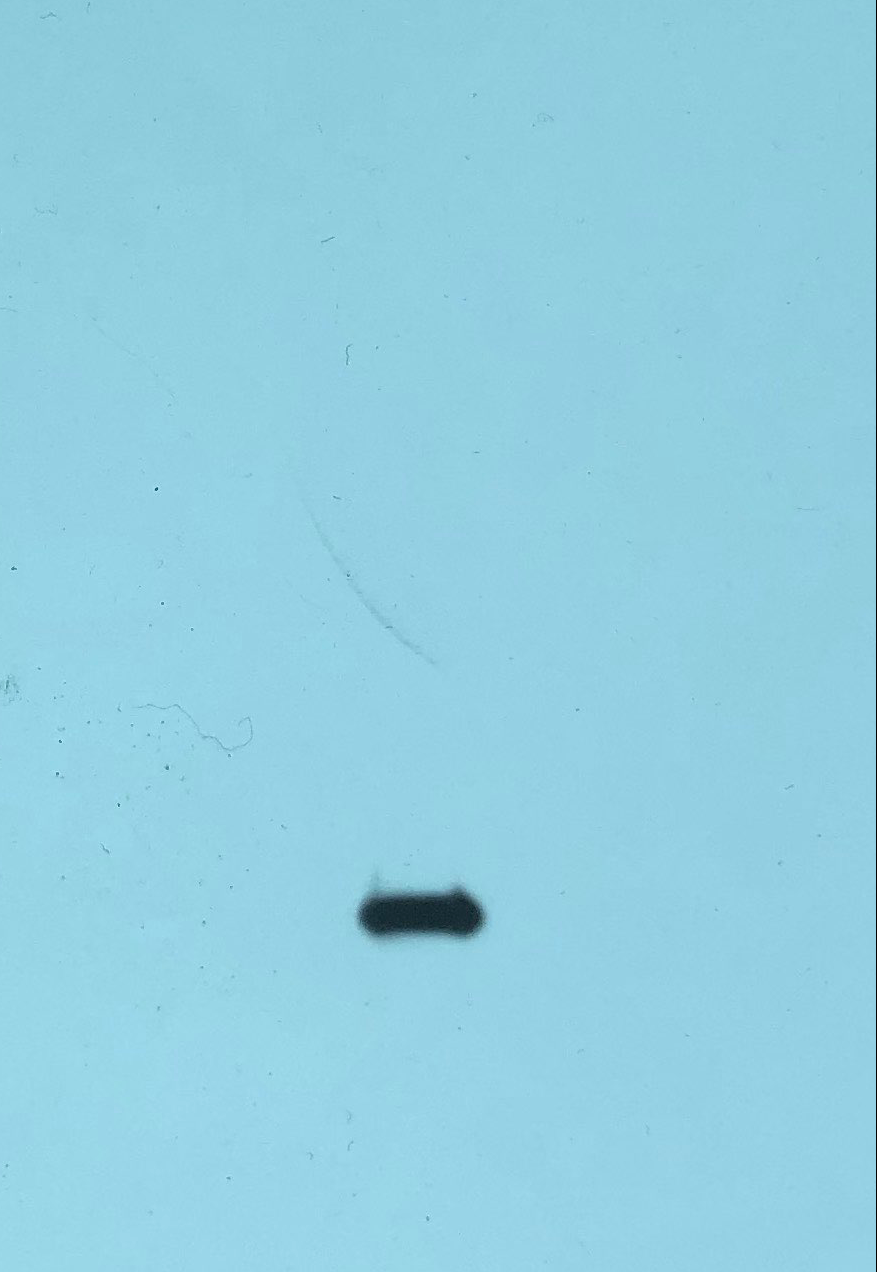

Supplement: Figure 4—source data 3. [file elife-89493-fig4-data3.zip › Figure 4 - source data 3/Raw_image_Figure 4D-IP-Mi2.tif]

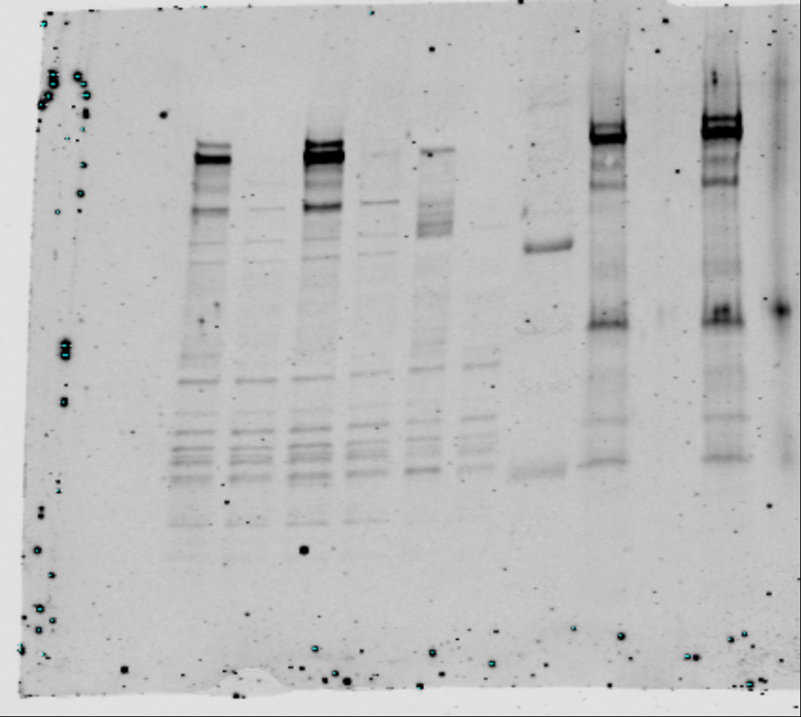

Supplement: Figure 4—source data 3. [file elife-89493-fig4-data3.zip › Figure 4 - source data 3/Raw_image_Figure 4D-Input-Bonus-intensity1.tif]

Figure 4D

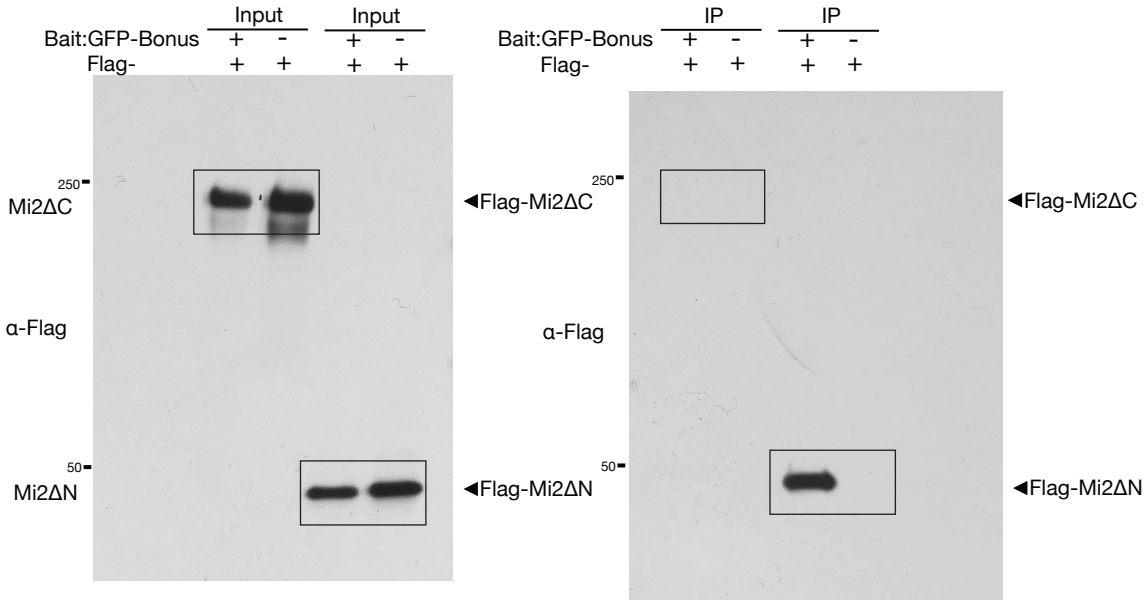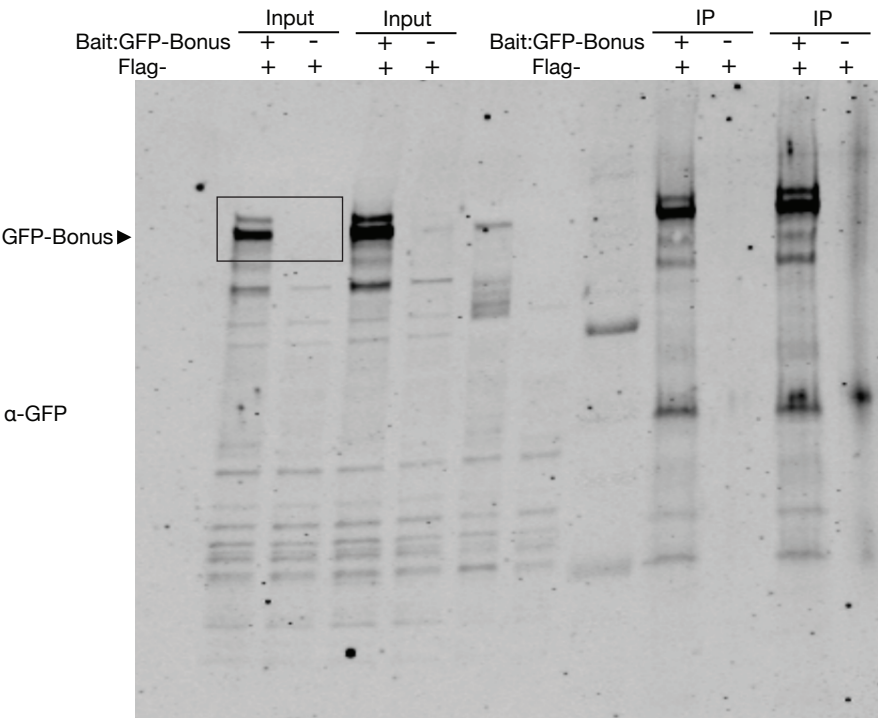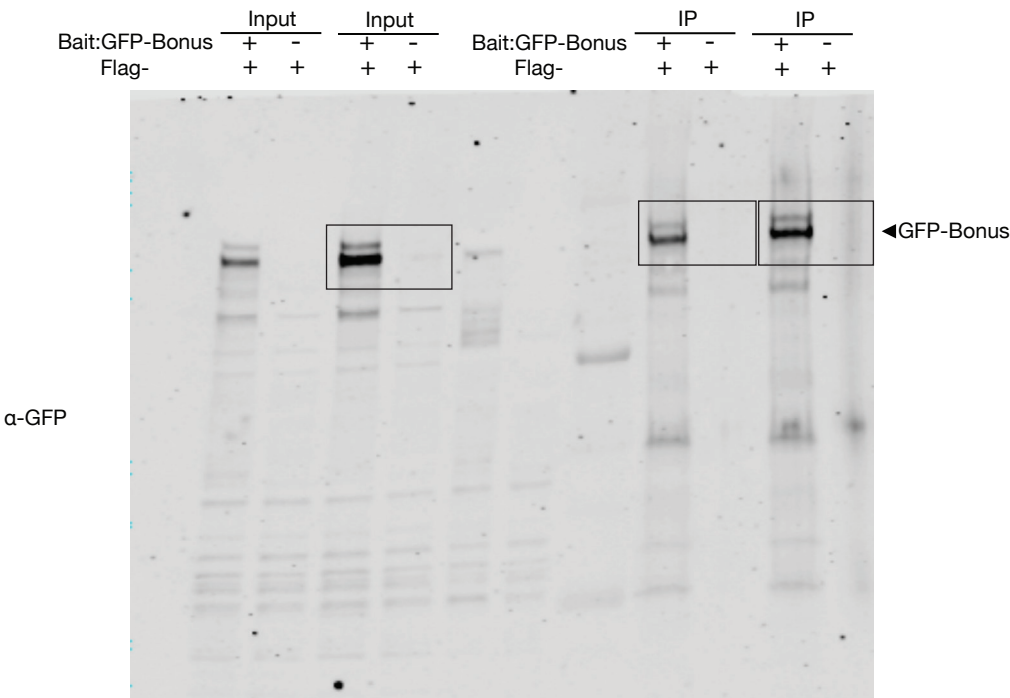

Supplement: Figure 4—source data 3. [file elife-89493-fig4-data3.zip › Figure 4 - source data 3/Figure4D-uncropped blot.pdf]

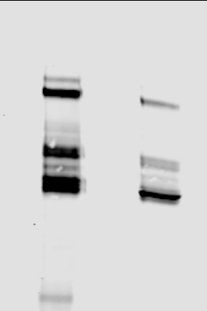

Supplement: Figure 4—figure supplement 1—source data 1. [file elife-89493-fig4-figsupp1-data1.zip › Figure 4 - figure supplement 1 - source data 1/Raw_image_Fig4-Fig-suppl1A-IP-Bonus.jpg]

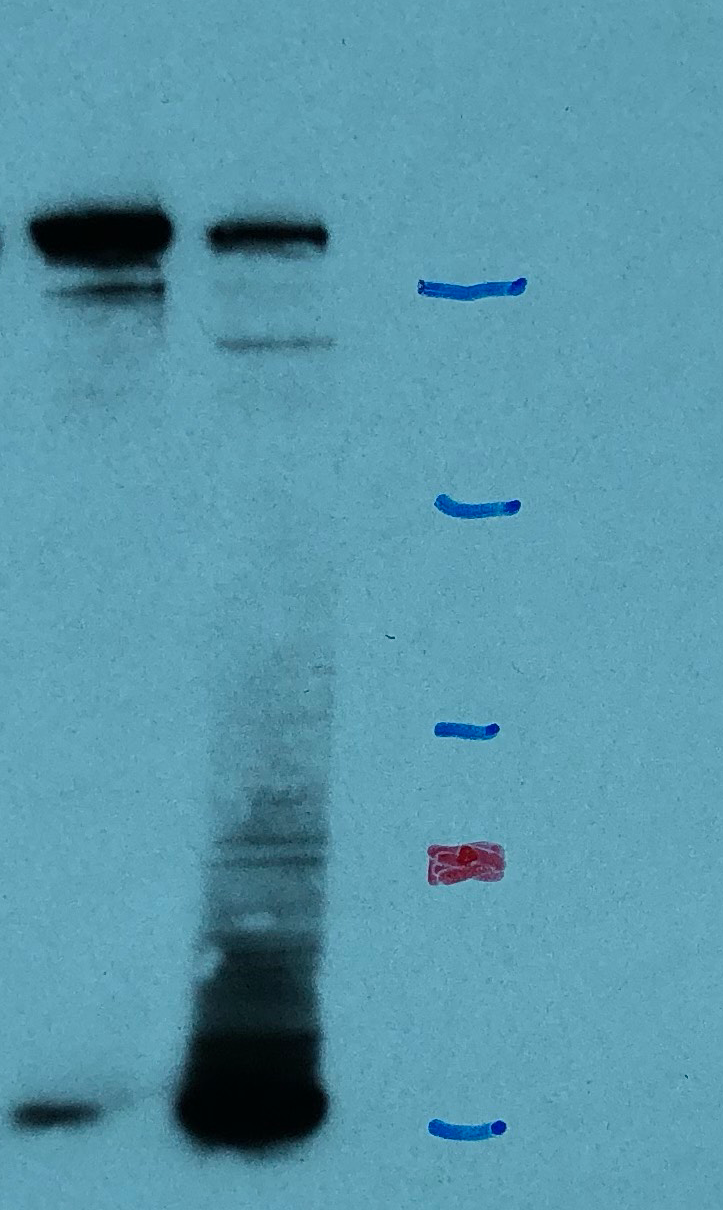

Supplement: Figure 4—figure supplement 1—source data 1. [file elife-89493-fig4-figsupp1-data1.zip › Figure 4 - figure supplement 1 - source data 1/Raw_image_Fig4-Fig-suppl1A-Input-Mi2.jpg]

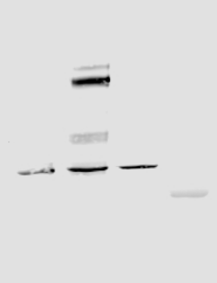

Supplement: Figure 4—figure supplement 1—source data 1. [file elife-89493-fig4-figsupp1-data1.zip › Figure 4 - figure supplement 1 - source data 1/Raw_image_Fig4-Fig-suppl1A-Input-Bonus.jpg]

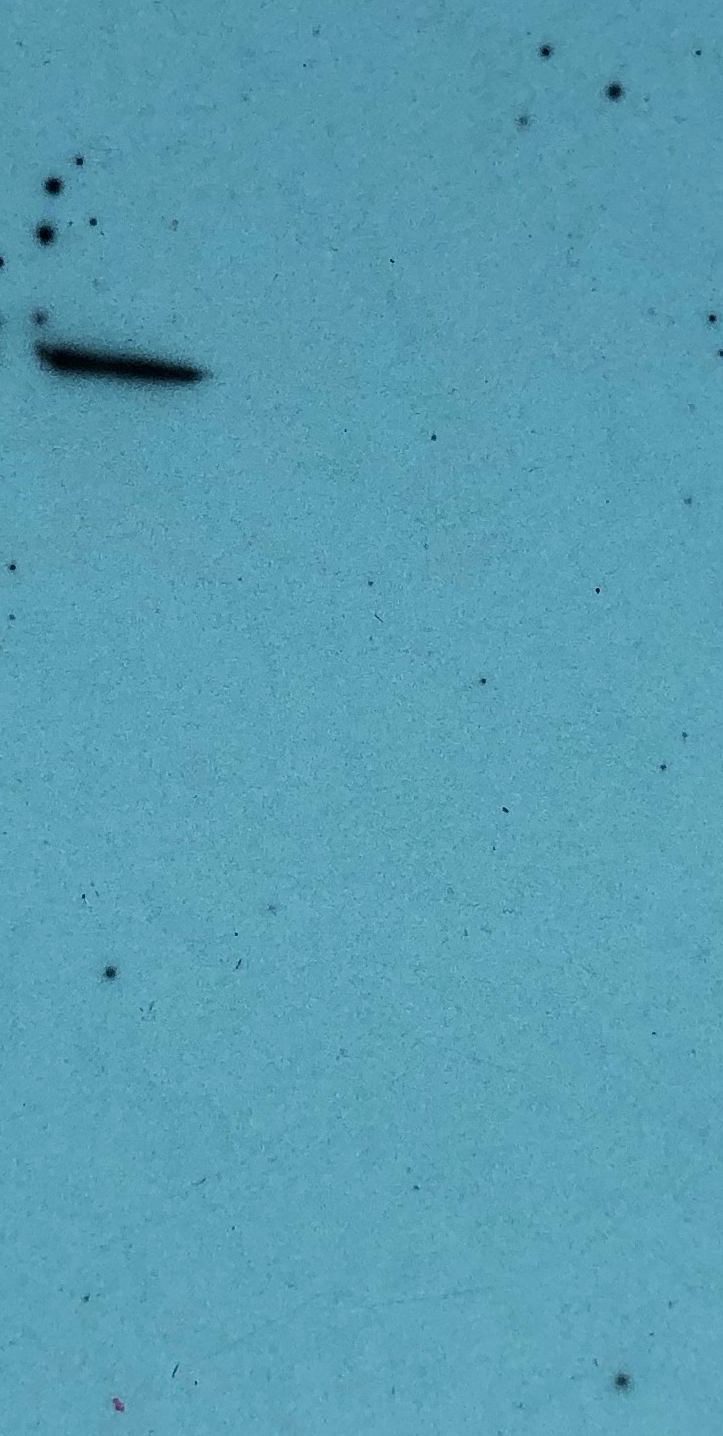

Supplement: Figure 4—figure supplement 1—source data 1. [file elife-89493-fig4-figsupp1-data1.zip › Figure 4 - figure supplement 1 - source data 1/Raw_image_Fig4-Fig-suppl1A-IP-Mi2.jpg]

**Figure 4 - figure supplement 1A**

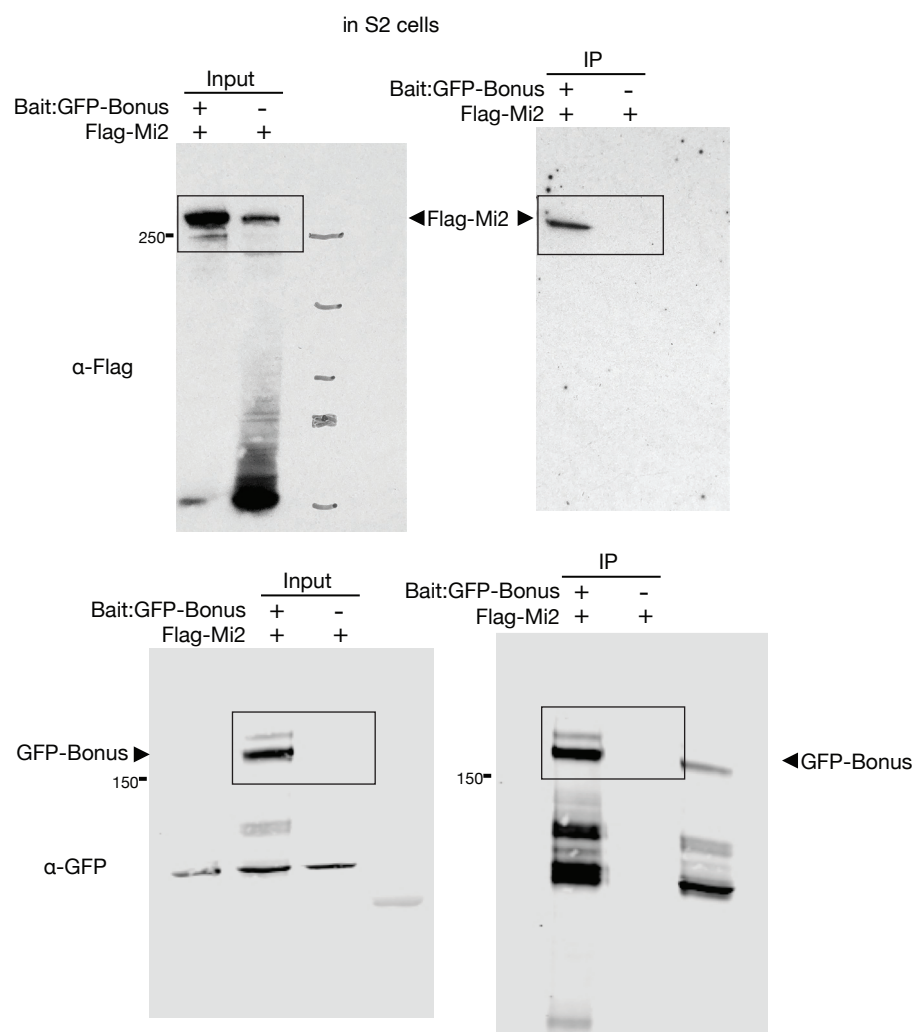

Supplement: Figure 4—figure supplement 1—source data 1. [file elife-89493-fig4-figsupp1-data1.zip › Figure 4 - figure supplement 1 - source data 1/Fig4-Figure suppl1A-uncropped blot.pdf]

Figure 4 - figure supplement 1B

in S2 cells

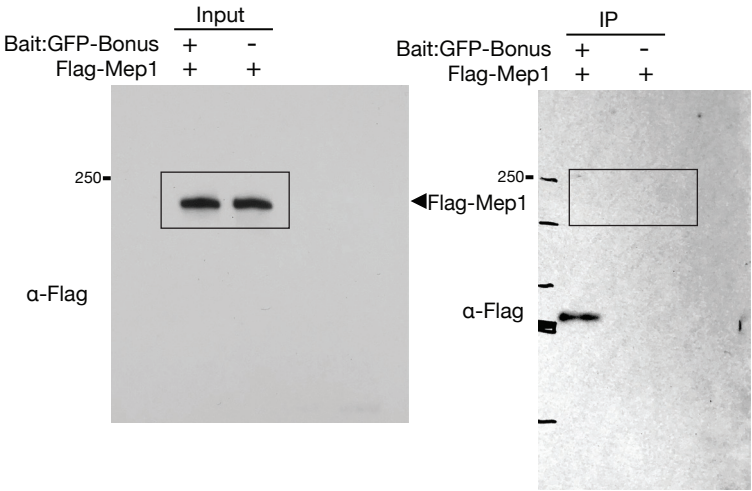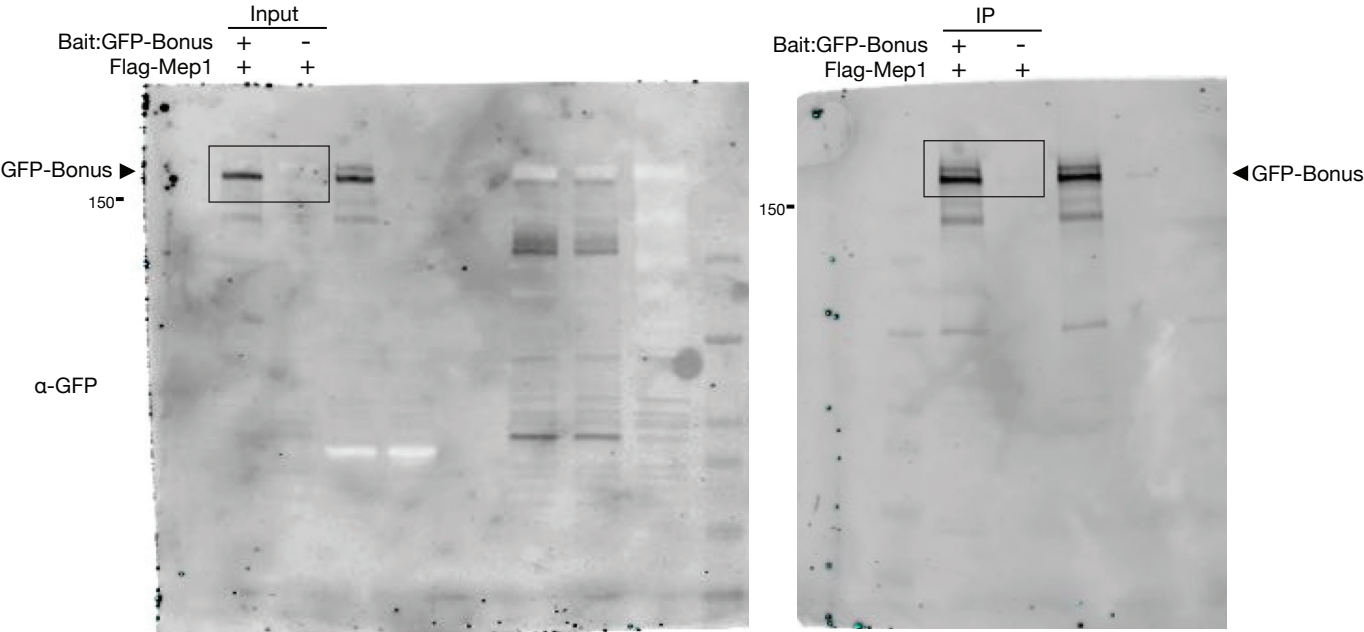

Supplement: Figure 4—figure supplement 1—source data 2. [file elife-89493-fig4-figsupp1-data2.zip › Figure 4 - figure supplement 1 - source data 2/Fig4-Figure suppl1B-uncropped blot.pdf]

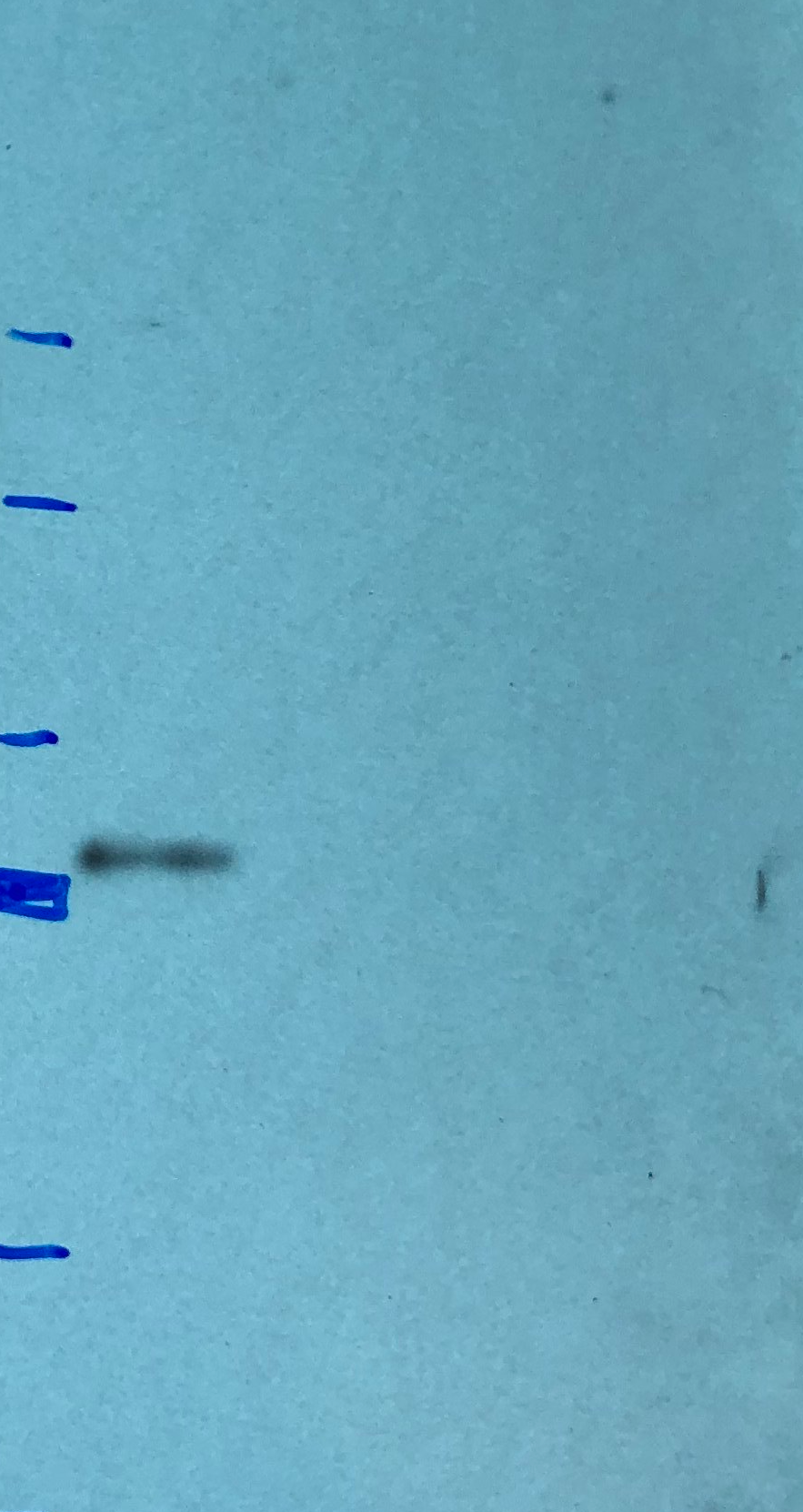

Supplement: Figure 4—figure supplement 1—source data 2. [file elife-89493-fig4-figsupp1-data2.zip › Figure 4 - figure supplement 1 - source data 2/Raw_image_Fig4-Fig-suppl1B-IP-Mep1.jpg]

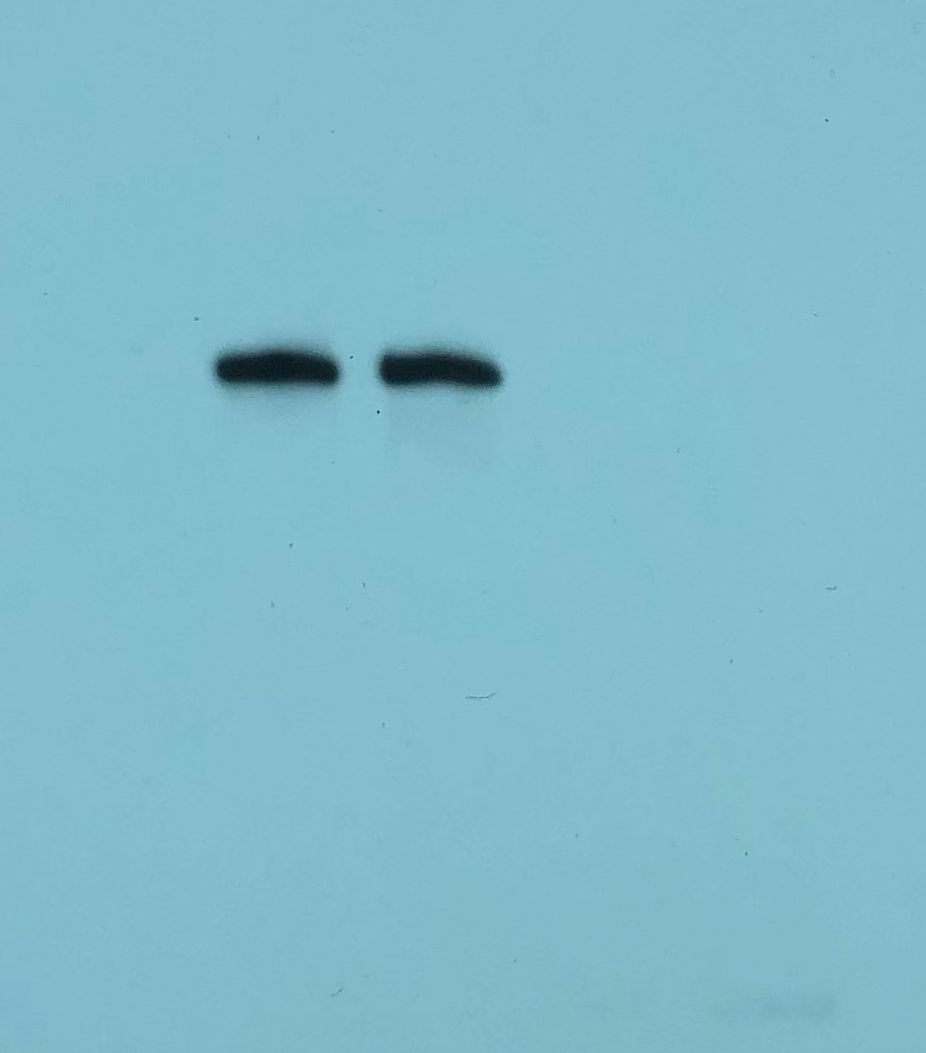

Supplement: Figure 4—figure supplement 1—source data 2. [file elife-89493-fig4-figsupp1-data2.zip › Figure 4 - figure supplement 1 - source data 2/Raw_image_Fig4-Fig-suppl1B-Input-Mep1.jpg]

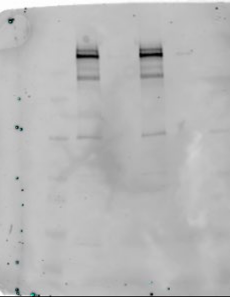

Supplement: Figure 4—figure supplement 1—source data 2. [file elife-89493-fig4-figsupp1-data2.zip › Figure 4 - figure supplement 1 - source data 2/Raw_image_Fig4-Fig-suppl1B-IP-Bonus.jpg]

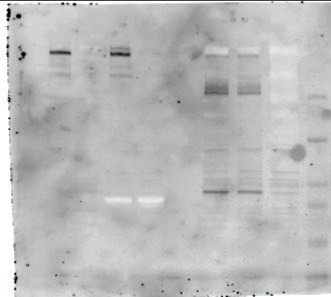

Supplement: Figure 4—figure supplement 1—source data 2. [file elife-89493-fig4-figsupp1-data2.zip › Figure 4 - figure supplement 1 - source data 2/Raw_image_Fig4-Fig-suppl1B-Input-Bonus.jpg]

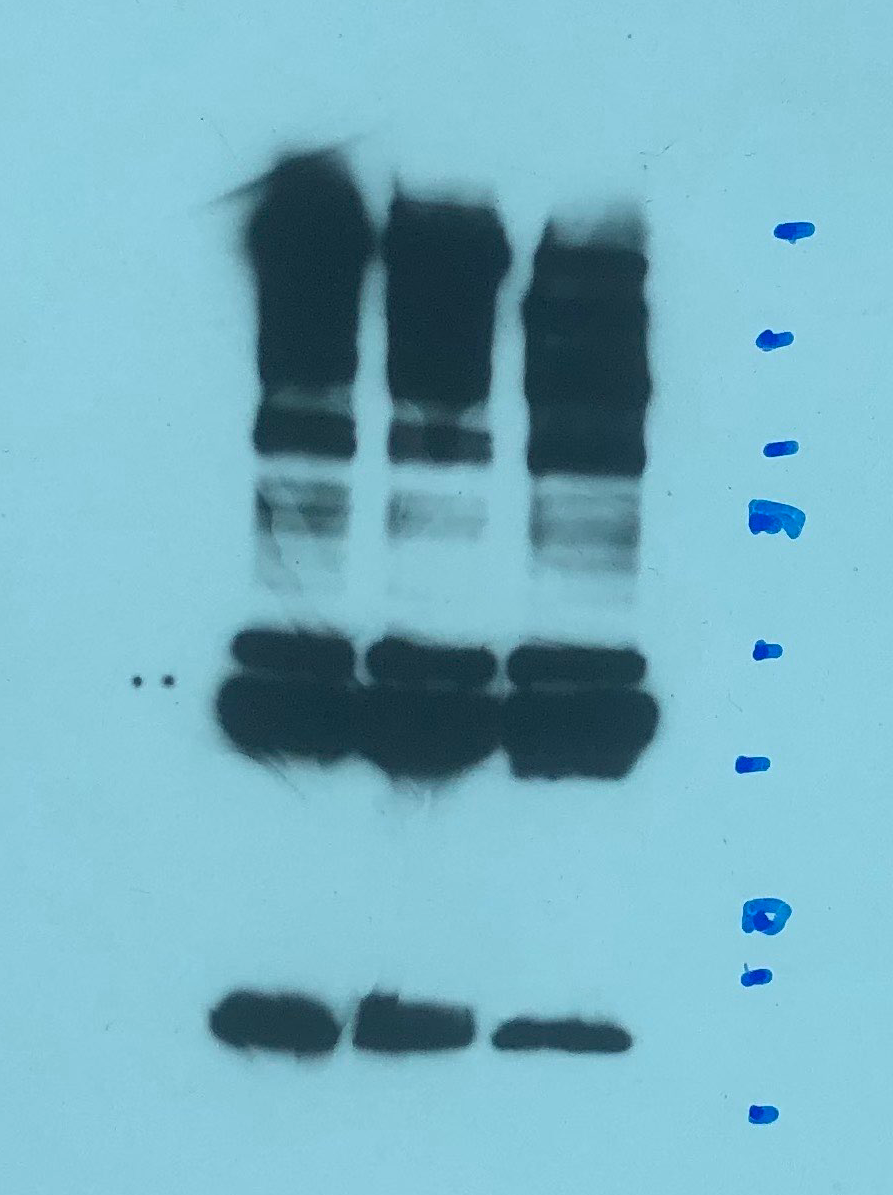

Supplement: Figure 5—source data 1. [file elife-89493-fig5-data1.zip › Figure 5 - source data 1/Raw_image_Figure5B-Input-SUMOylated.tif]

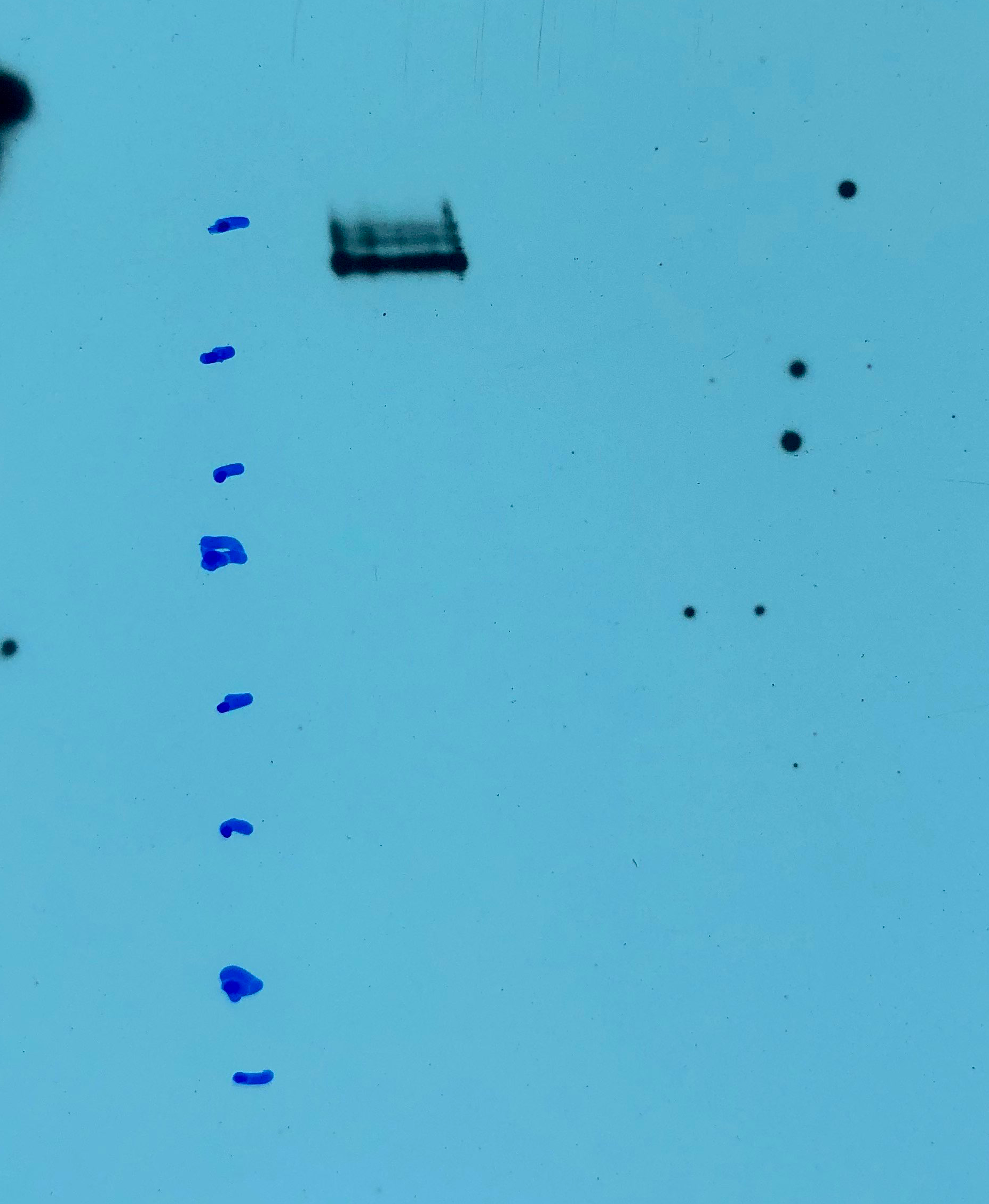

Supplement: Figure 5—source data 1. [file elife-89493-fig5-data1.zip › Figure 5 - source data 1/Raw_image_Figure5B-IP-SUMOylated.tif]

Figure 5B.

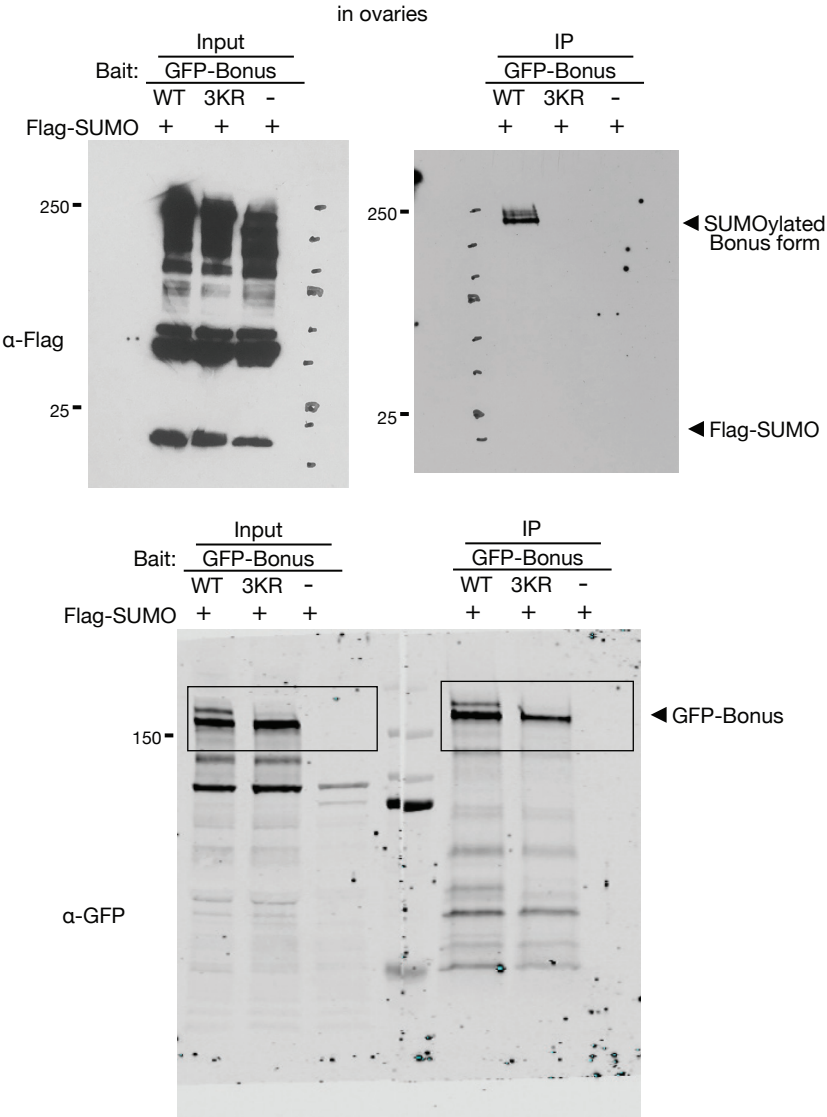

Supplement: Figure 5—source data 1. [file elife-89493-fig5-data1.zip › Figure 5 - source data 1/Figure5B-uncropped blot.pdf]

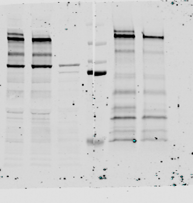

Supplement: Figure 5—source data 1. [file elife-89493-fig5-data1.zip › Figure 5 - source data 1/Raw_image_Figure5B-Bonus.tif]

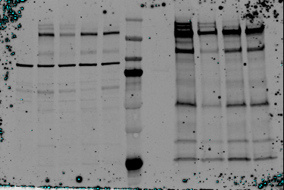

Supplement: Figure 5—source data 2. [file elife-89493-fig5-data2.zip › Figure 5 - source data 2/Raw_image_Figure5C-Bonus-intensity1.tif]

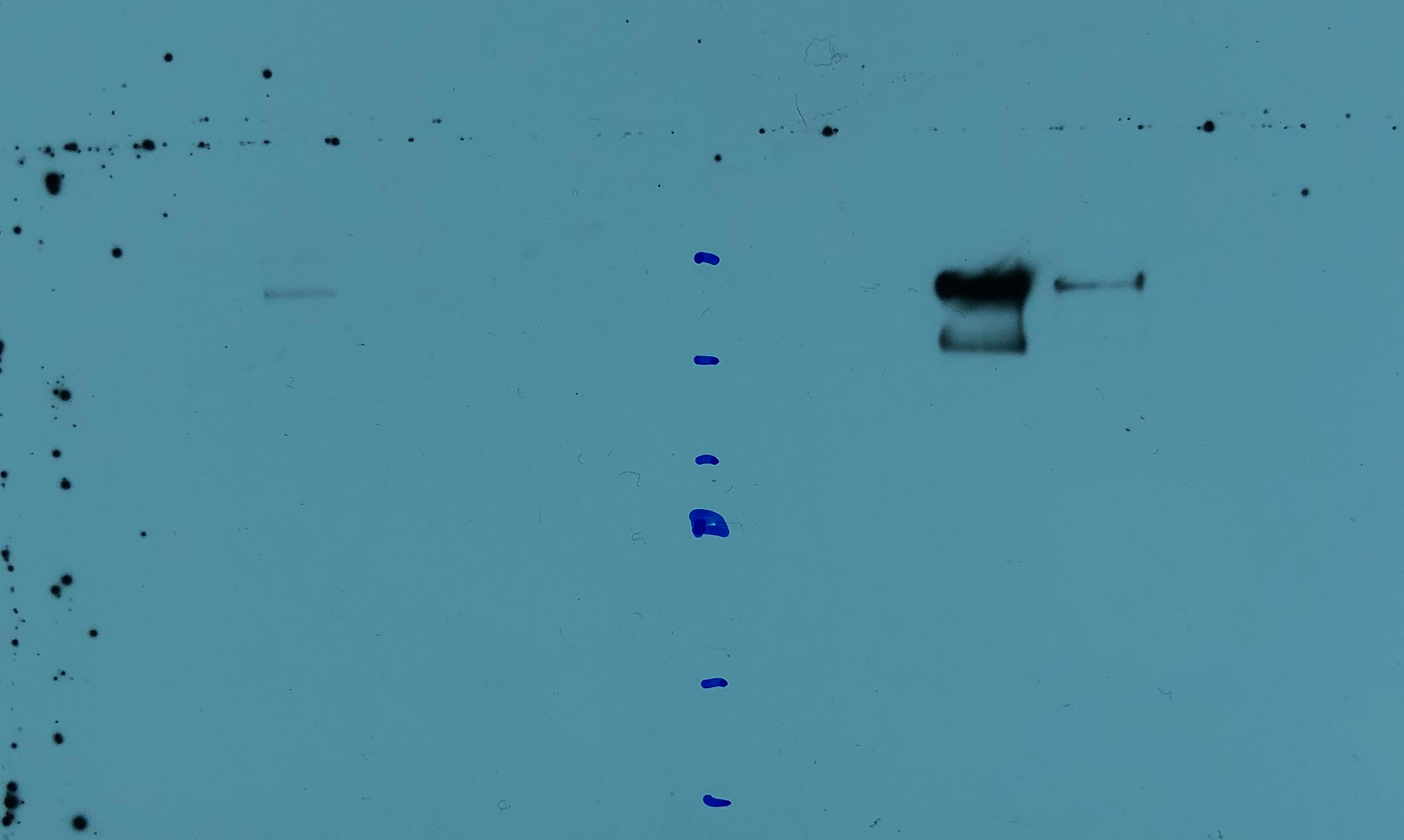

Supplement: Figure 5—source data 2. [file elife-89493-fig5-data2.zip › Figure 5 - source data 2/Raw_image_Figure5C-Input-IP-HA-SUMO.tif]

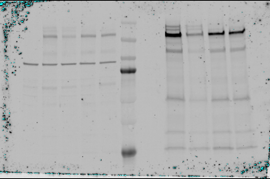

Supplement: Figure 5—source data 2. [file elife-89493-fig5-data2.zip › Figure 5 - source data 2/Raw_image_Figure5C-Bonus-intensity2.tif]

Figure 5C.

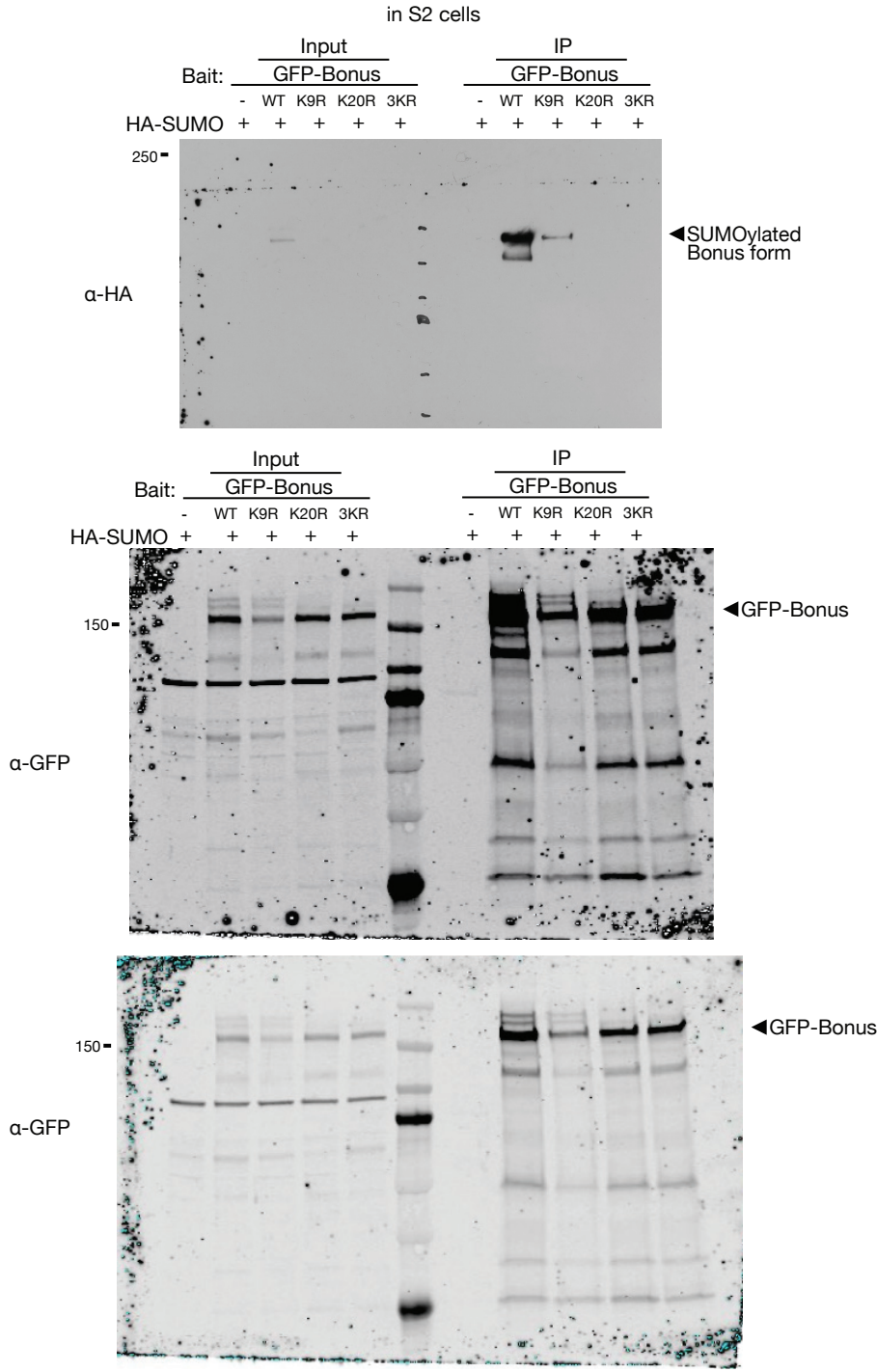

Supplement: Figure 5—source data 2. [file elife-89493-fig5-data2.zip › Figure 5 - source data 2/Figure5C-uncropped blot.pdf]

Figure 5F.

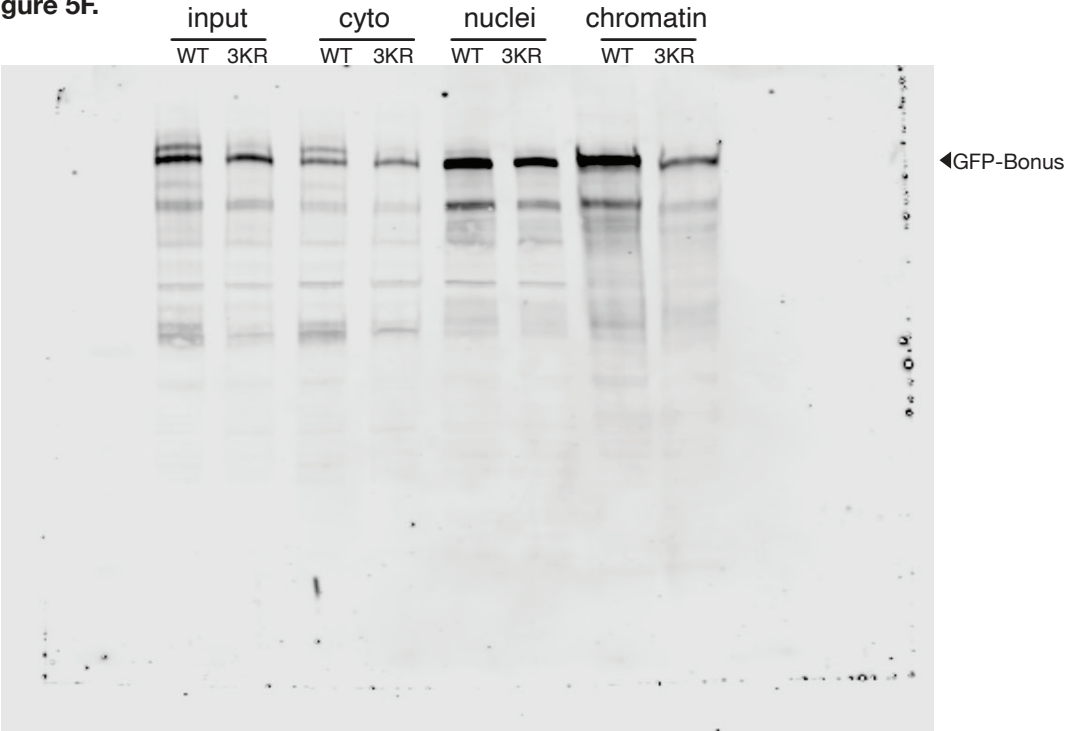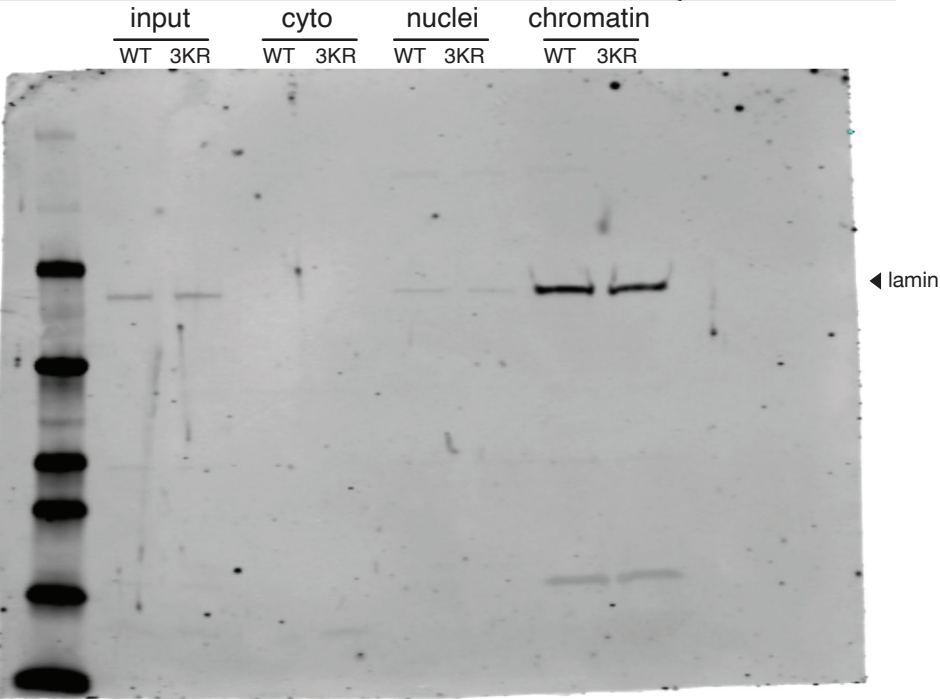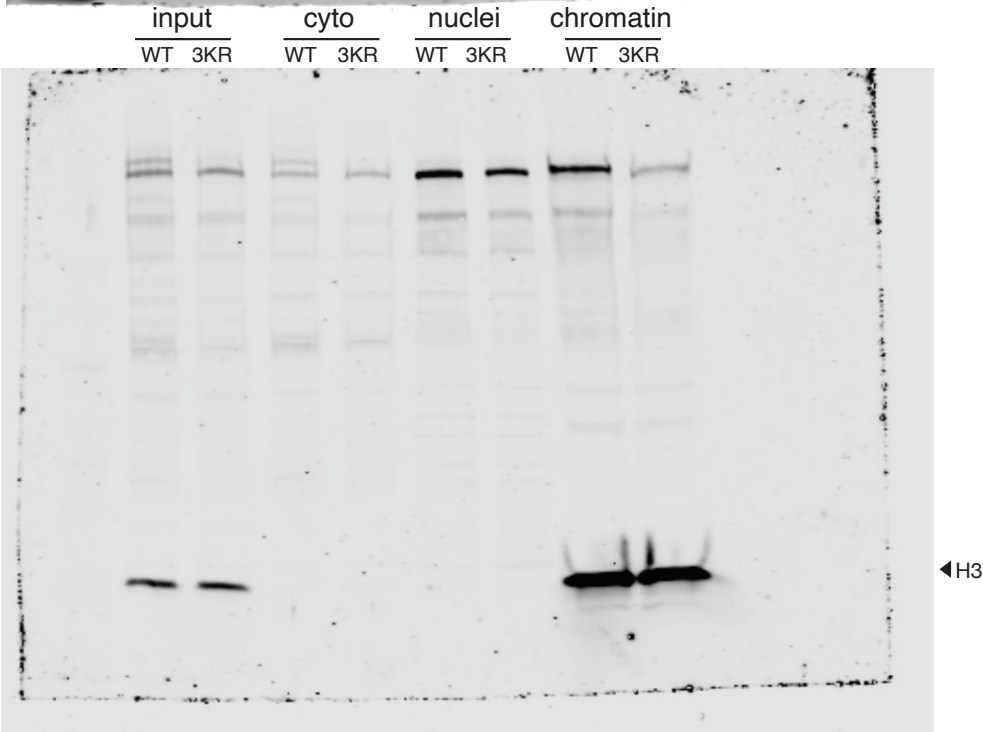

Supplement: Figure 5—source data 3. [file elife-89493-fig5-data3.zip › Figure 5 - source data 3/Figure5F-uncropped blot.pdf]

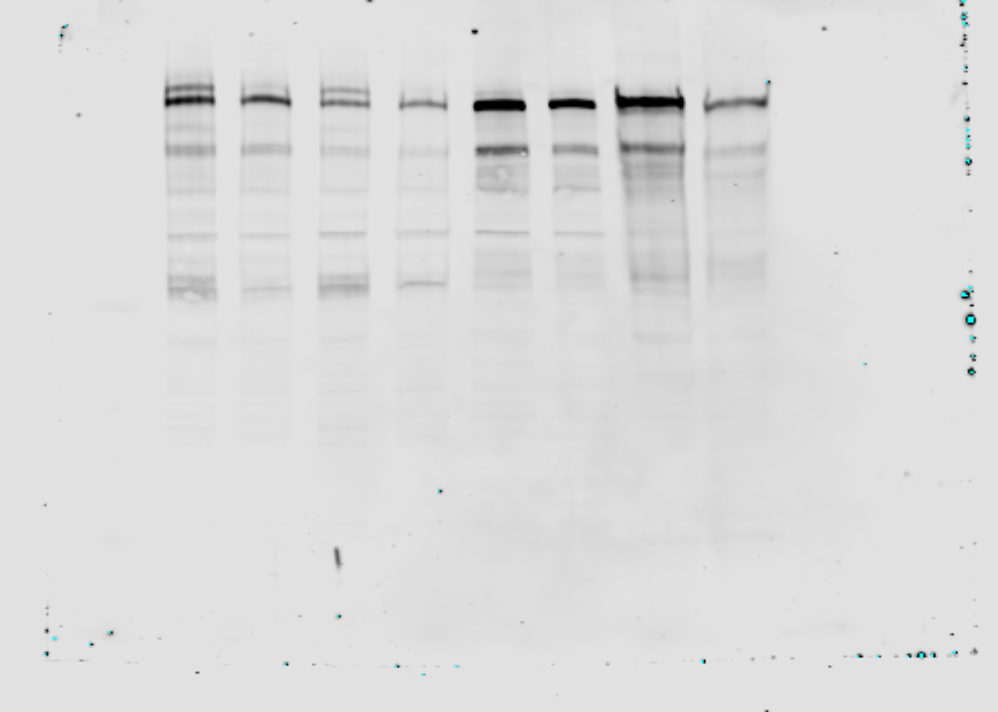

Supplement: Figure 5—source data 3. [file elife-89493-fig5-data3.zip › Figure 5 - source data 3/Raw_image_Figure5F-Bonus.tif]

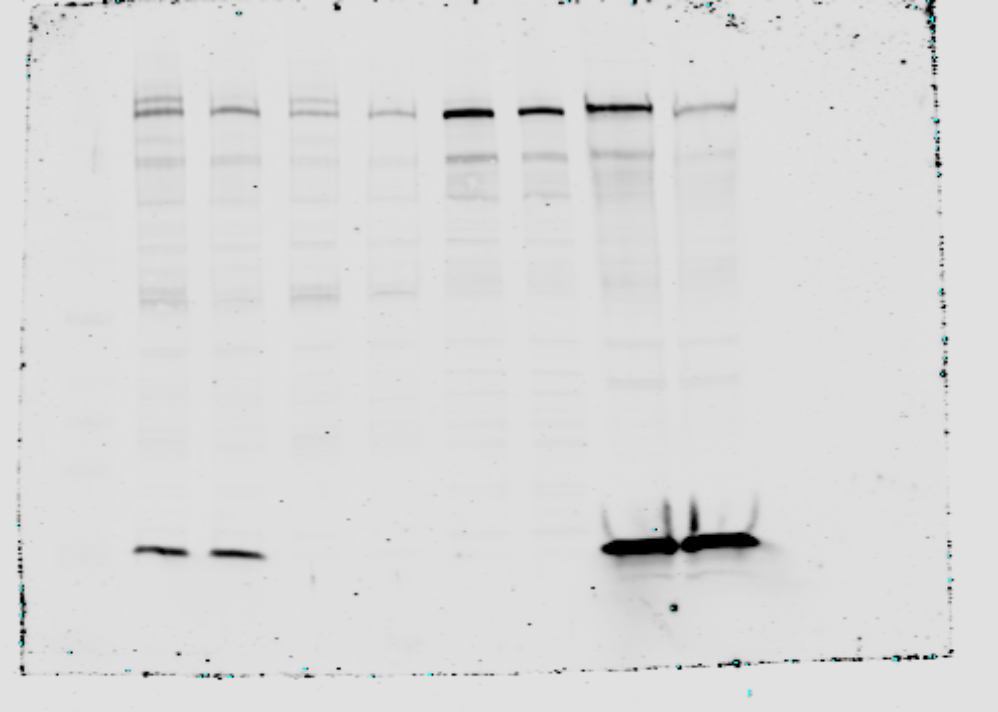

Supplement: Figure 5—source data 3. [file elife-89493-fig5-data3.zip › Figure 5 - source data 3/Raw_image_Figure5F-H3.tif]

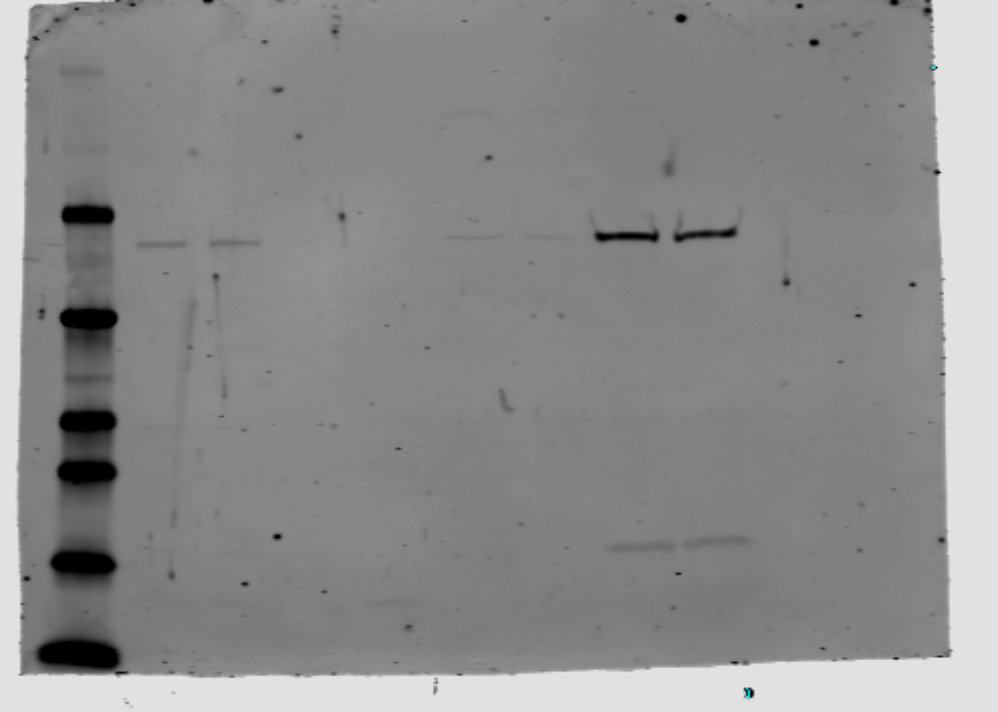

Supplement: Figure 5—source data 3. [file elife-89493-fig5-data3.zip › Figure 5 - source data 3/Raw_image_Figure5F-lamin.tif]

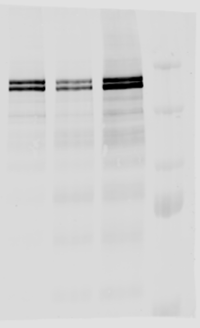

Supplement: Figure 5—source data 4. [file elife-89493-fig5-data4.zip › Figure 5 - source data 4/Raw_image_Figure5I-Input-SetDB1.tif]

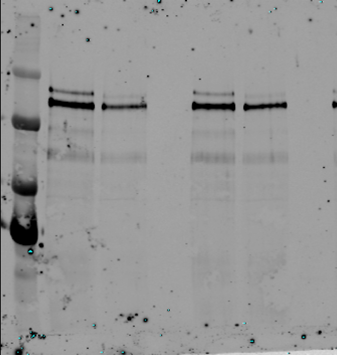

Supplement: Figure 5—source data 4. [file elife-89493-fig5-data4.zip › Figure 5 - source data 4/Raw_image_Figure5I-IP-Bonus.tif]

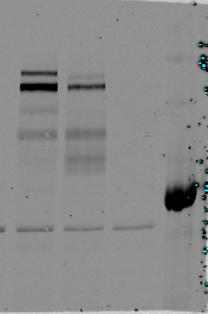

Supplement: Figure 5—source data 4. [file elife-89493-fig5-data4.zip › Figure 5 - source data 4/Raw_image_Figure5I-Input-Bonus.tif]

Figure 5I.

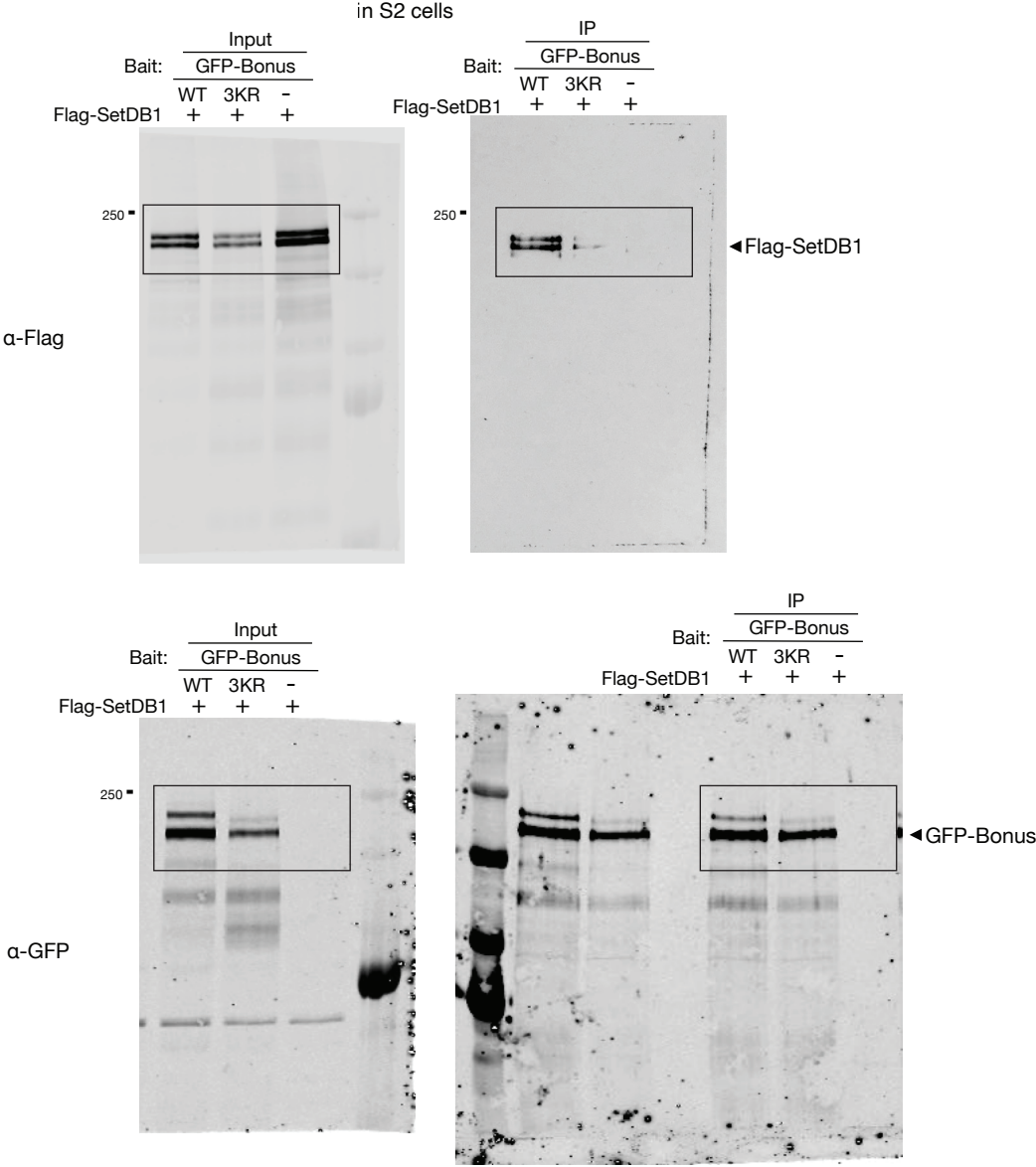

Supplement: Figure 5—source data 4. [file elife-89493-fig5-data4.zip › Figure 5 - source data 4/Figure5I-uncropped blot.pdf]

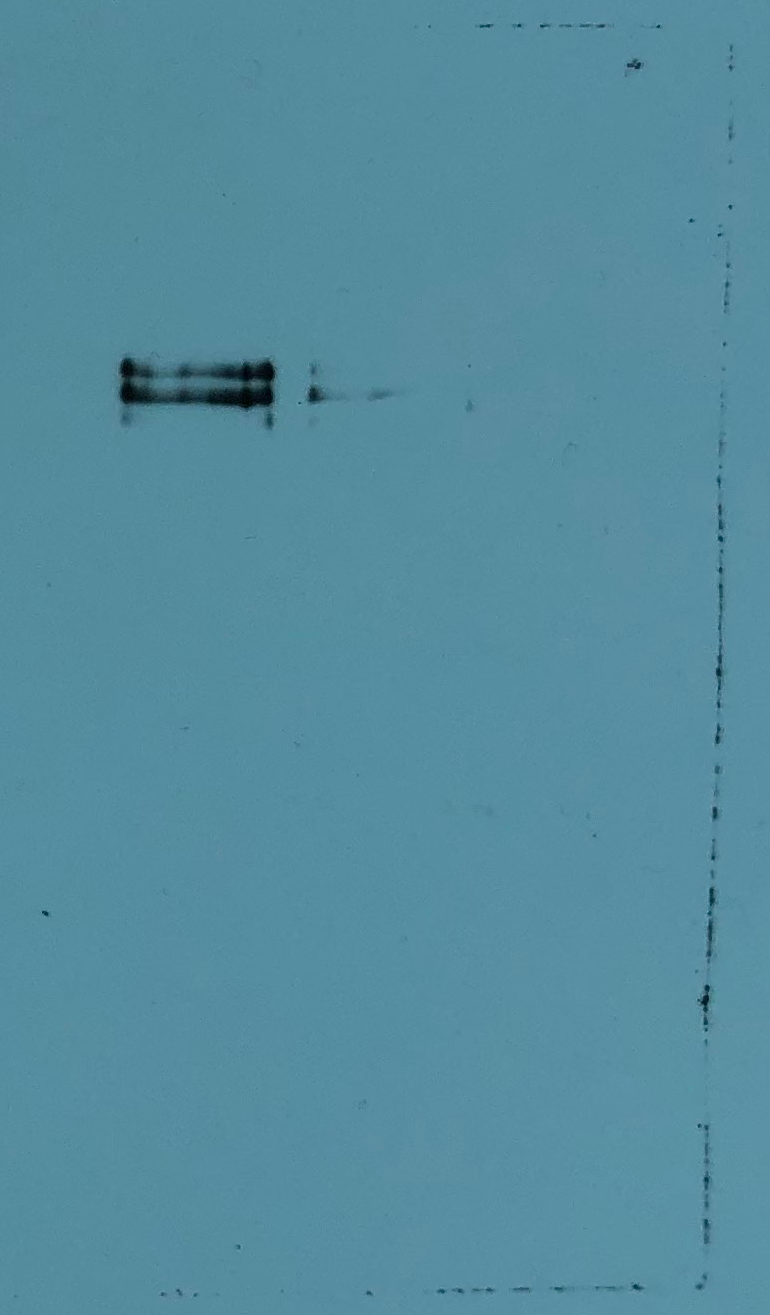

Supplement: Figure 5—source data 4. [file elife-89493-fig5-data4.zip › Figure 5 - source data 4/Raw_image_Figure5I-IP-SetDB1.tif]

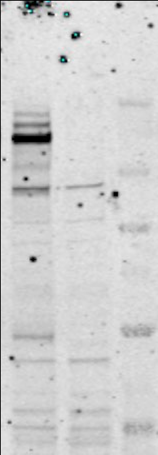

Supplement: Figure 5—figure supplement 1—source data 1. [file elife-89493-fig5-figsupp1-data1.zip › Figure 5 - figure supplement 1 - source data 1/Raw_image_Fig5-Fig-suppl1A-Input-Bonus.jpg]

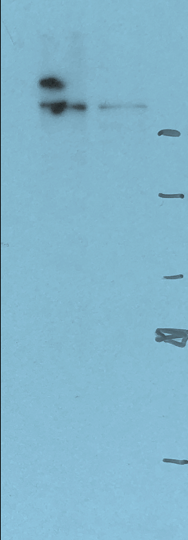

Supplement: Figure 5—figure supplement 1—source data 1. [file elife-89493-fig5-figsupp1-data1.zip › Figure 5 - figure supplement 1 - source data 1/Raw_image_Fig5-Fig-suppl1A-Input-SUMO.jpg]

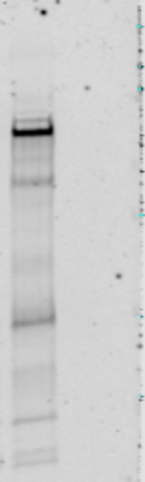

Supplement: Figure 5—figure supplement 1—source data 1. [file elife-89493-fig5-figsupp1-data1.zip › Figure 5 - figure supplement 1 - source data 1/Raw_image_Fig5-Fig-suppl1A-IP-Bonus.jpg]

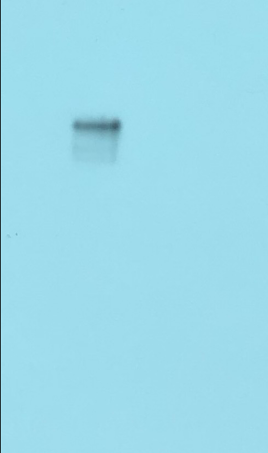

Supplement: Figure 5—figure supplement 1—source data 1. [file elife-89493-fig5-figsupp1-data1.zip › Figure 5 - figure supplement 1 - source data 1/Raw_image_Fig5-Fig-suppl1A-IP-SUMO.jpg]

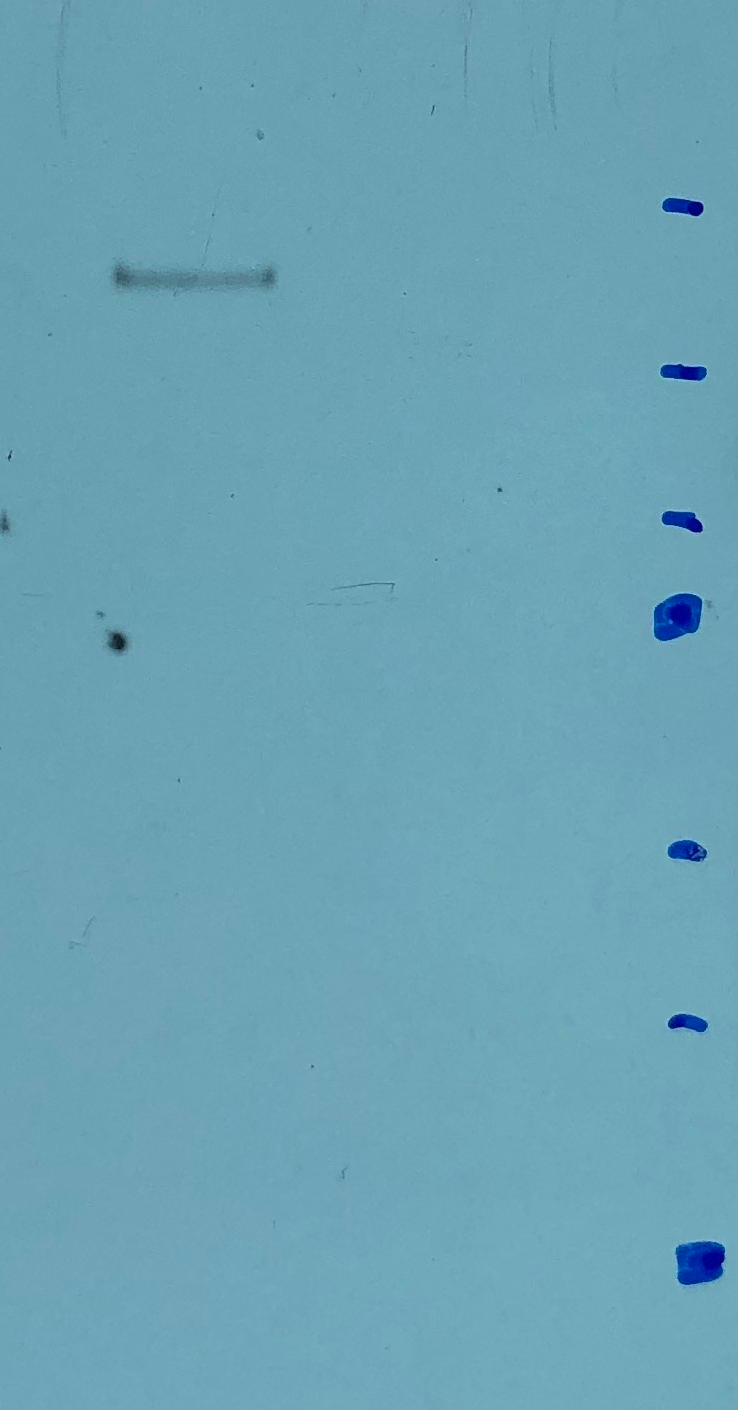

Supplement: Figure 5—figure supplement 1—source data 1. [file elife-89493-fig5-figsupp1-data1.zip › Figure 5 - figure supplement 1 - source data 1/Raw_image_Fig5-Fig-suppl1A-IP-SUMO-bottom.jpg]

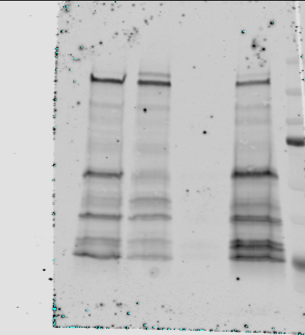

Supplement: Figure 5—figure supplement 1—source data 1. [file elife-89493-fig5-figsupp1-data1.zip › Figure 5 - figure supplement 1 - source data 1/Raw_image_Fig5-Fig-suppl1A-IP-Bonus-bottom.jpg]

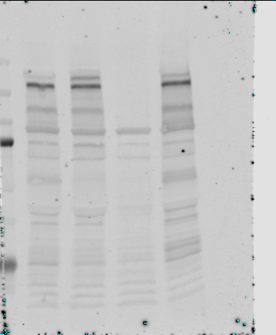

Supplement: Figure 5—figure supplement 1—source data 1. [file elife-89493-fig5-figsupp1-data1.zip › Figure 5 - figure supplement 1 - source data 1/Raw_image_Fig5-Fig-suppl1A-Input-Bonus-bottom.jpg]

Figure 5 - figure supplement 1A

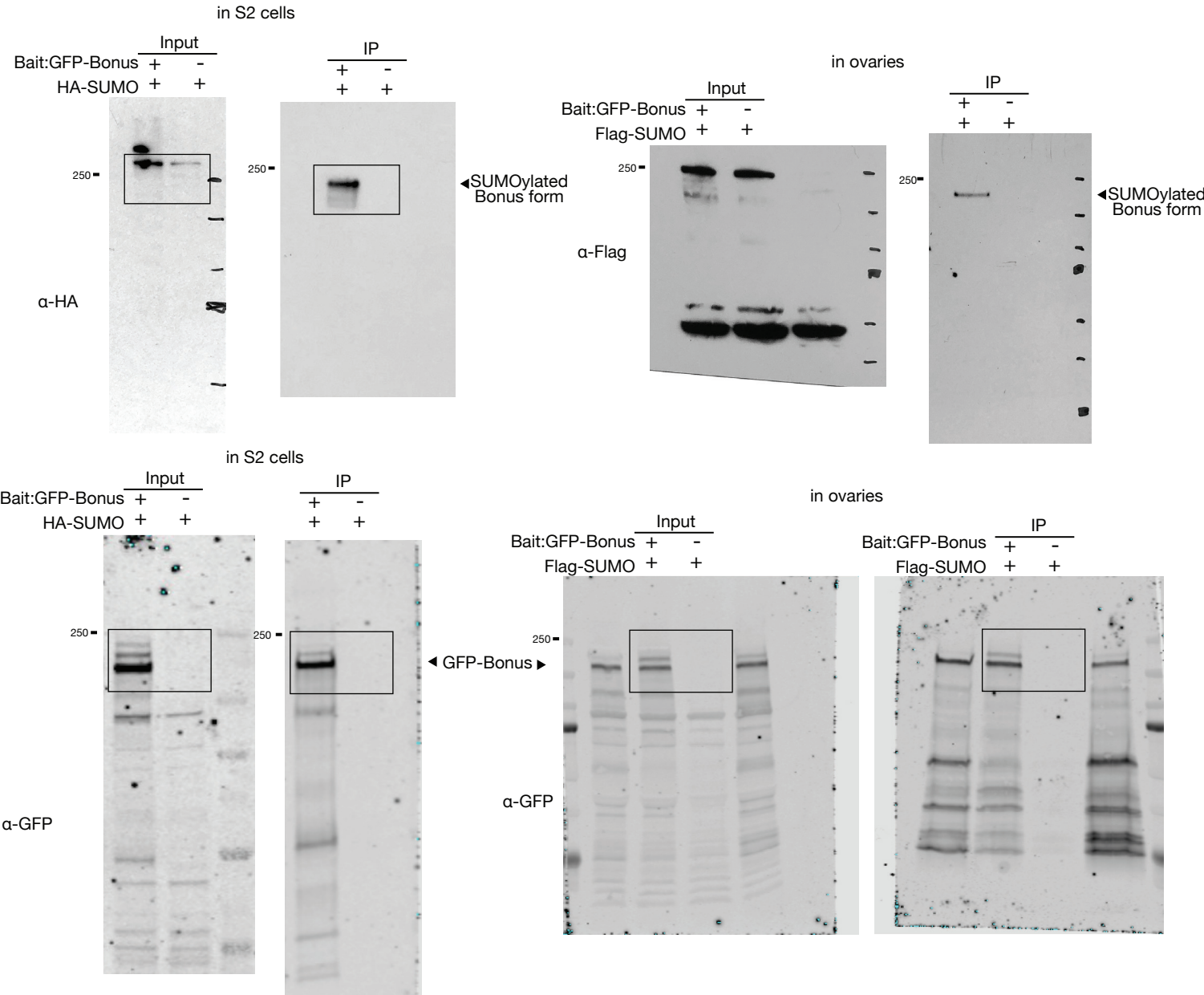

Supplement: Figure 5—figure supplement 1—source data 1. [file elife-89493-fig5-figsupp1-data1.zip › Figure 5 - figure supplement 1 - source data 1/Fig5-Figure suppl1A-uncropped blot.pdf]

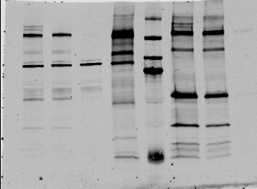

Supplement: Figure 5—figure supplement 1—source data 2. [file elife-89493-fig5-figsupp1-data2.zip › Figure 5 - figure supplement 1 - source data 2/Raw_image_Fig5-Fig-suppl1B-Bonus.jpg]

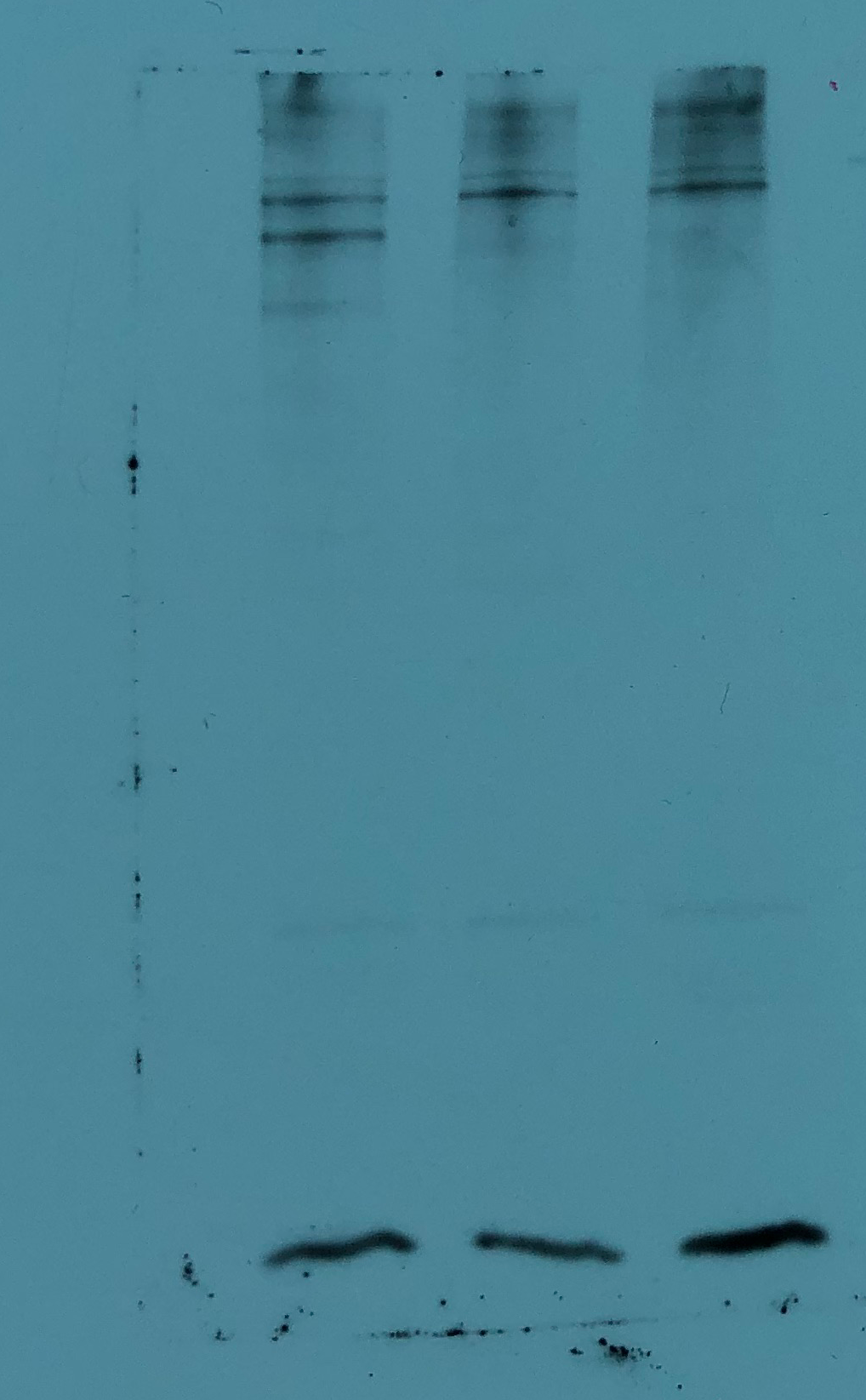

Supplement: Figure 5—figure supplement 1—source data 2. [file elife-89493-fig5-figsupp1-data2.zip › Figure 5 - figure supplement 1 - source data 2/Raw_image_Fig5-Fig-suppl1B-Input-SUMO.jpg]

Figure 5 - figure supplement 1B

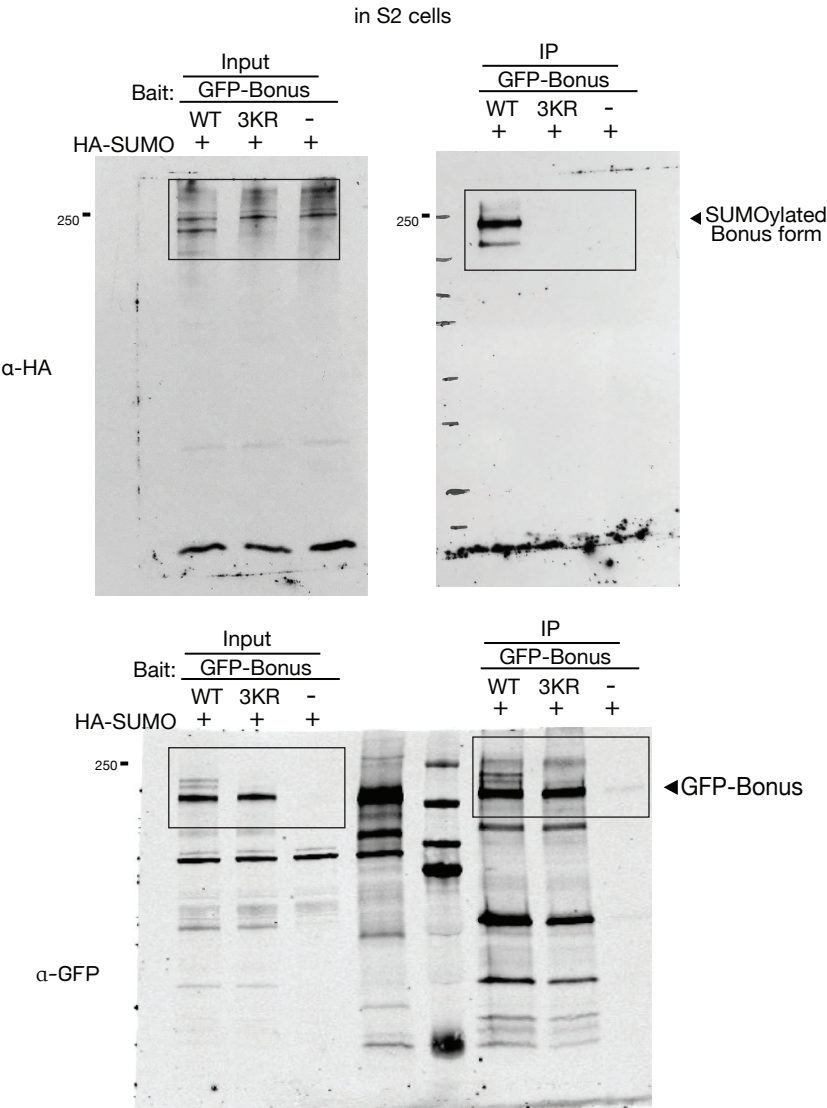

Supplement: Figure 5—figure supplement 1—source data 2. [file elife-89493-fig5-figsupp1-data2.zip › Figure 5 - figure supplement 1 - source data 2/Fig5-Figure suppl1B-uncropped blot.pdf]

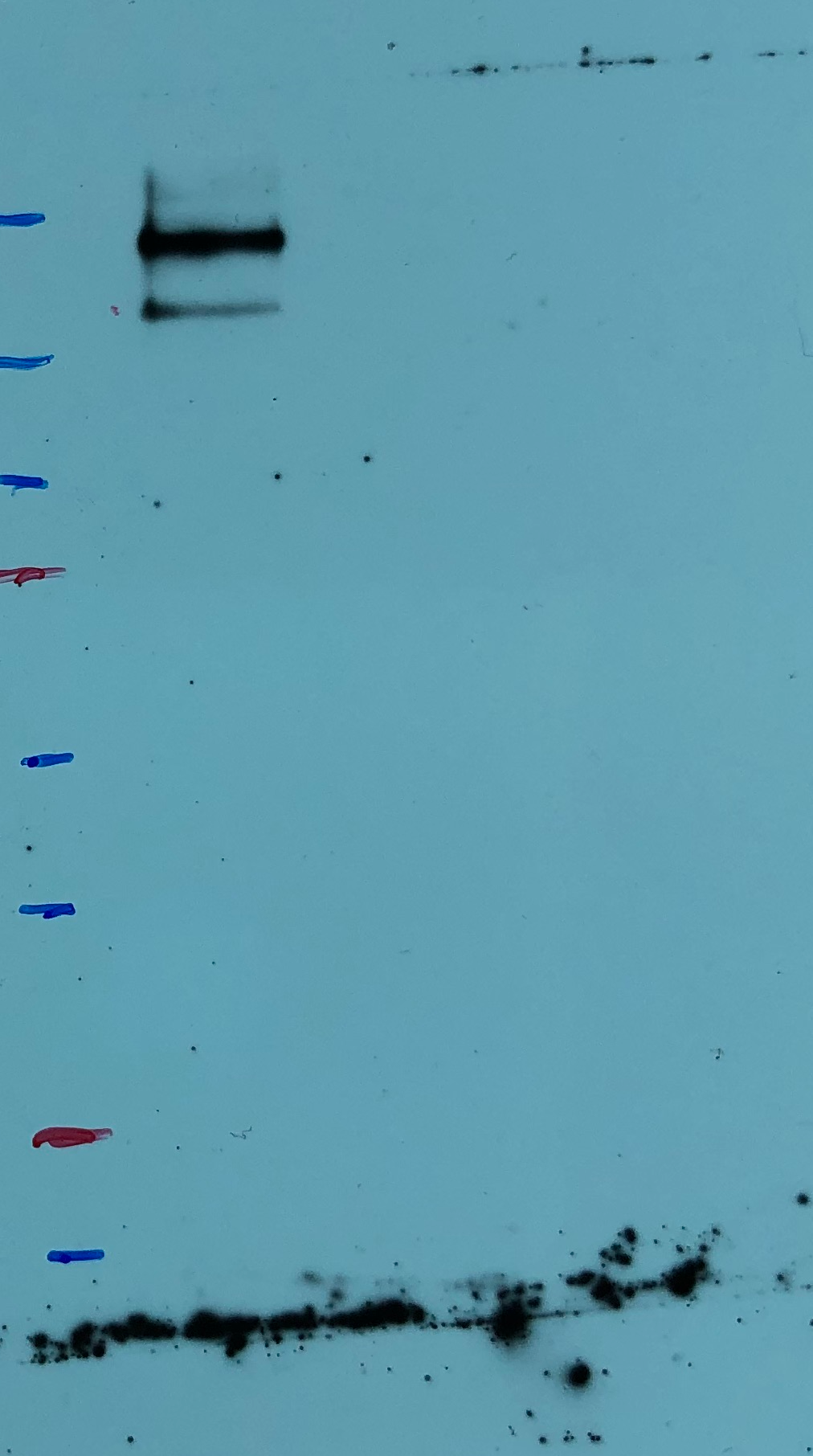

Supplement: Figure 5—figure supplement 1—source data 2. [file elife-89493-fig5-figsupp1-data2.zip › Figure 5 - figure supplement 1 - source data 2/Raw_image_Fig5-Fig-suppl1B-IP-SUMO.jpg]

Figure 5 - figure supplement 1C

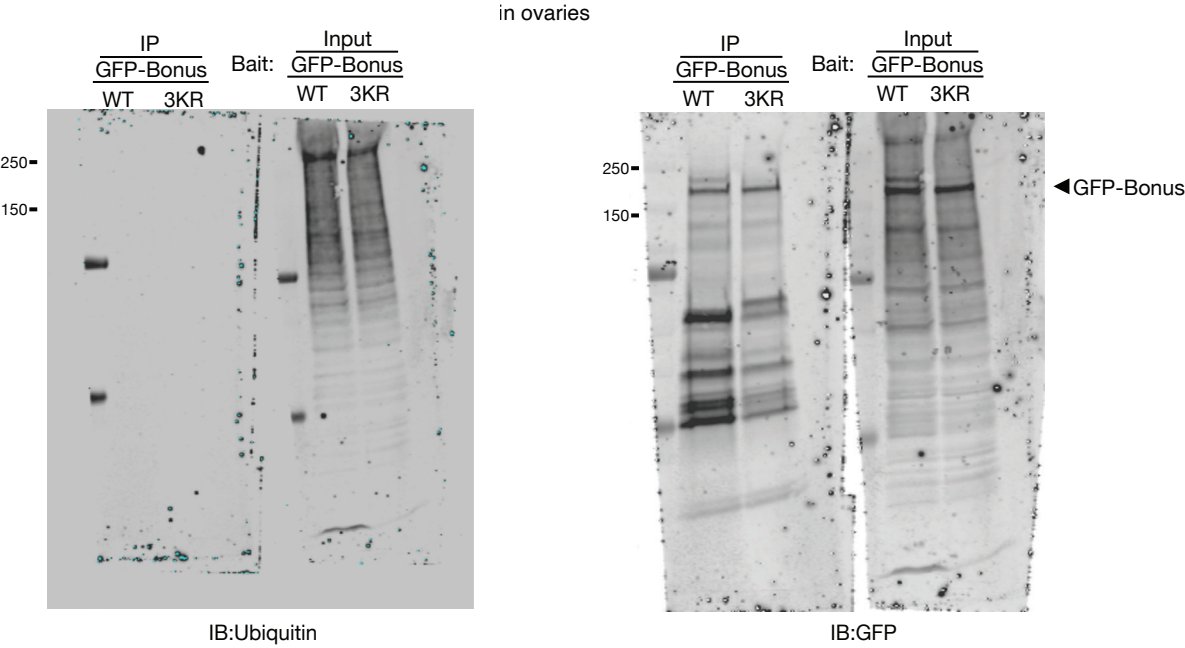

Supplement: Figure 5—figure supplement 1—source data 3. [file elife-89493-fig5-figsupp1-data3.zip › Figure 5 - figure supplement 1 - source data 3/Fig5-Figure suppl1C-uncropped blot.pdf]

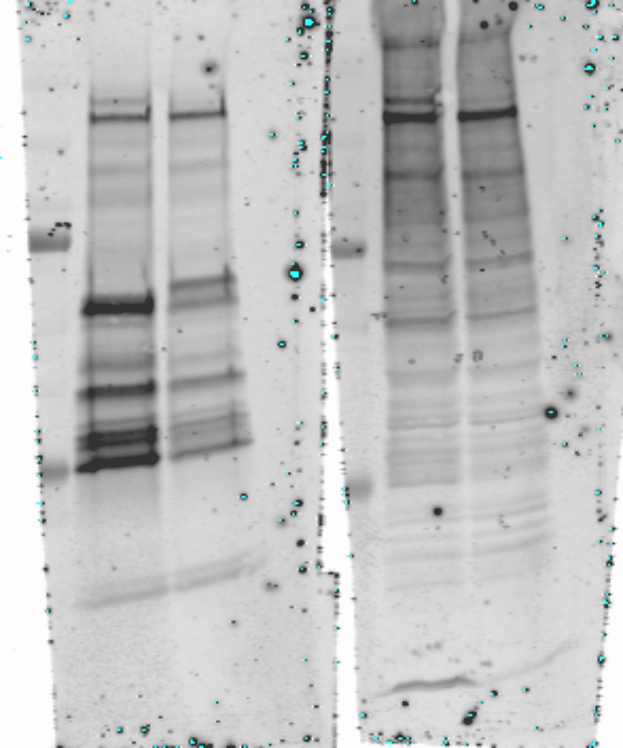

Supplement: Figure 5—figure supplement 1—source data 3. [file elife-89493-fig5-figsupp1-data3.zip › Figure 5 - figure supplement 1 - source data 3/Raw_image_Fig5-Fig-suppl1C-Bonus.tif]

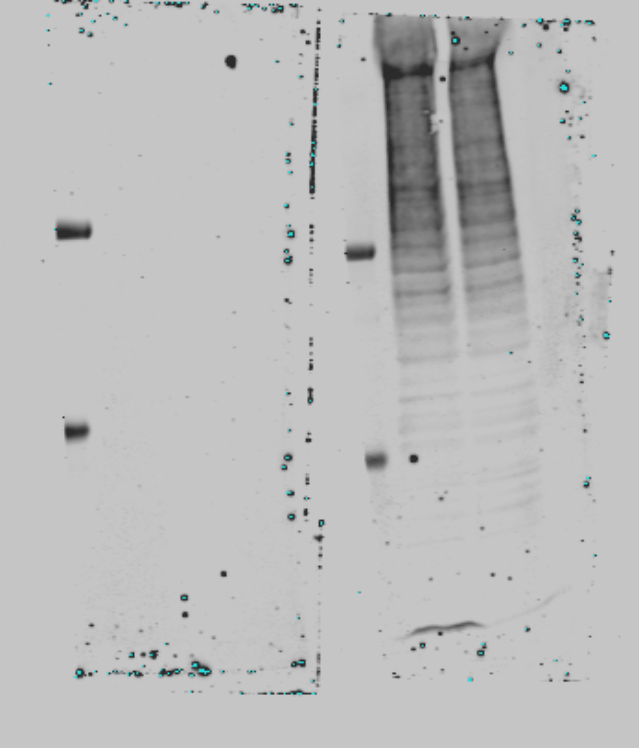

Supplement: Figure 5—figure supplement 1—source data 3. [file elife-89493-fig5-figsupp1-data3.zip › Figure 5 - figure supplement 1 - source data 3/Raw_image_Fig5-Fig-suppl1C-Ubiquitin.tif]

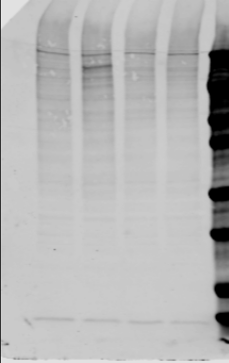

Supplement: Figure 5—figure supplement 1—source data 4. [file elife-89493-fig5-figsupp1-data4.zip › Figure 5 - figure supplement 1 - source data 4/Raw_image_Fig5-Fig-suppl1D-Input-SUMO+NEM.jpg]

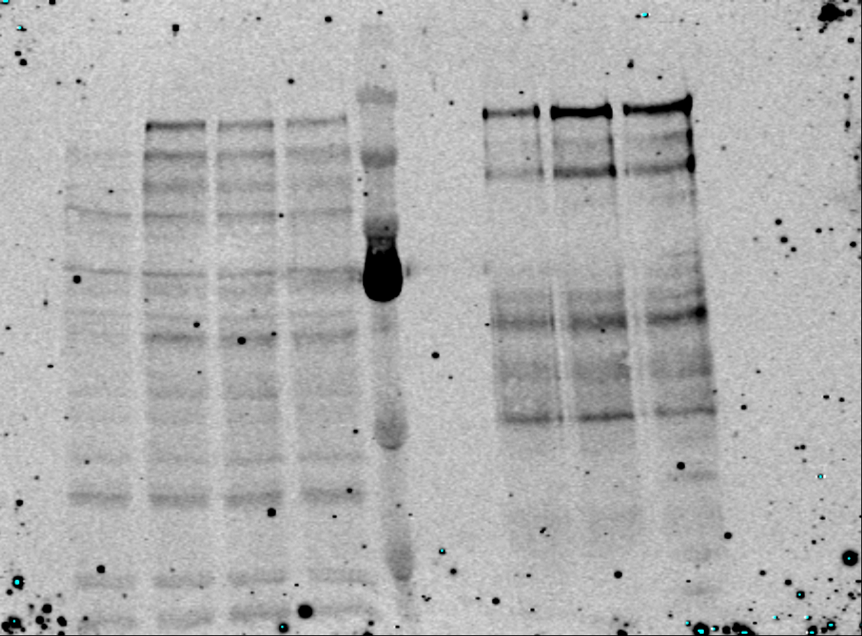

Supplement: Figure 5—figure supplement 1—source data 4. [file elife-89493-fig5-figsupp1-data4.zip › Figure 5 - figure supplement 1 - source data 4/Raw_image_Fig5-Fig-suppl1D-Bonus-NEM.jpg]

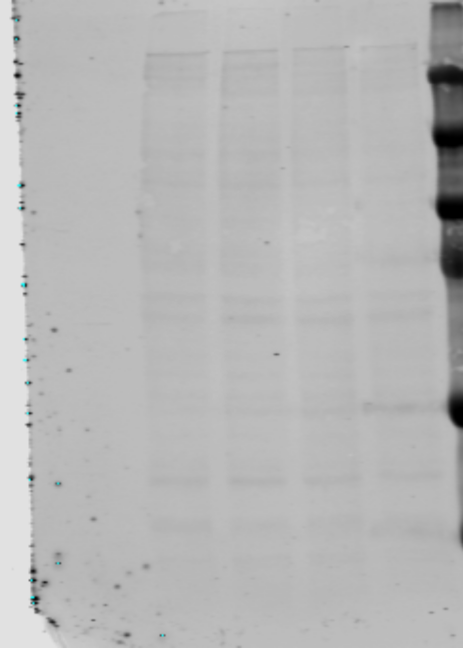

Supplement: Figure 5—figure supplement 1—source data 4. [file elife-89493-fig5-figsupp1-data4.zip › Figure 5 - figure supplement 1 - source data 4/Raw_image_Fig5-Fig-suppl1D-Input-SUMO-NEM.jpg]

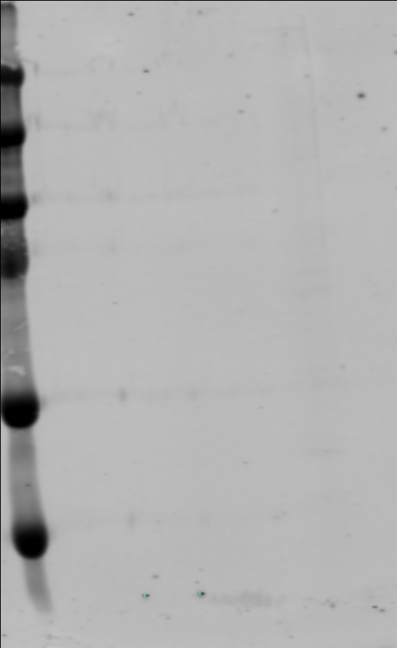

Supplement: Figure 5—figure supplement 1—source data 4. [file elife-89493-fig5-figsupp1-data4.zip › Figure 5 - figure supplement 1 - source data 4/Raw_image_Fig5-Fig-suppl1D-IP-SUMO-NEM.jpg]

Figure 5 - figure supplement 1D

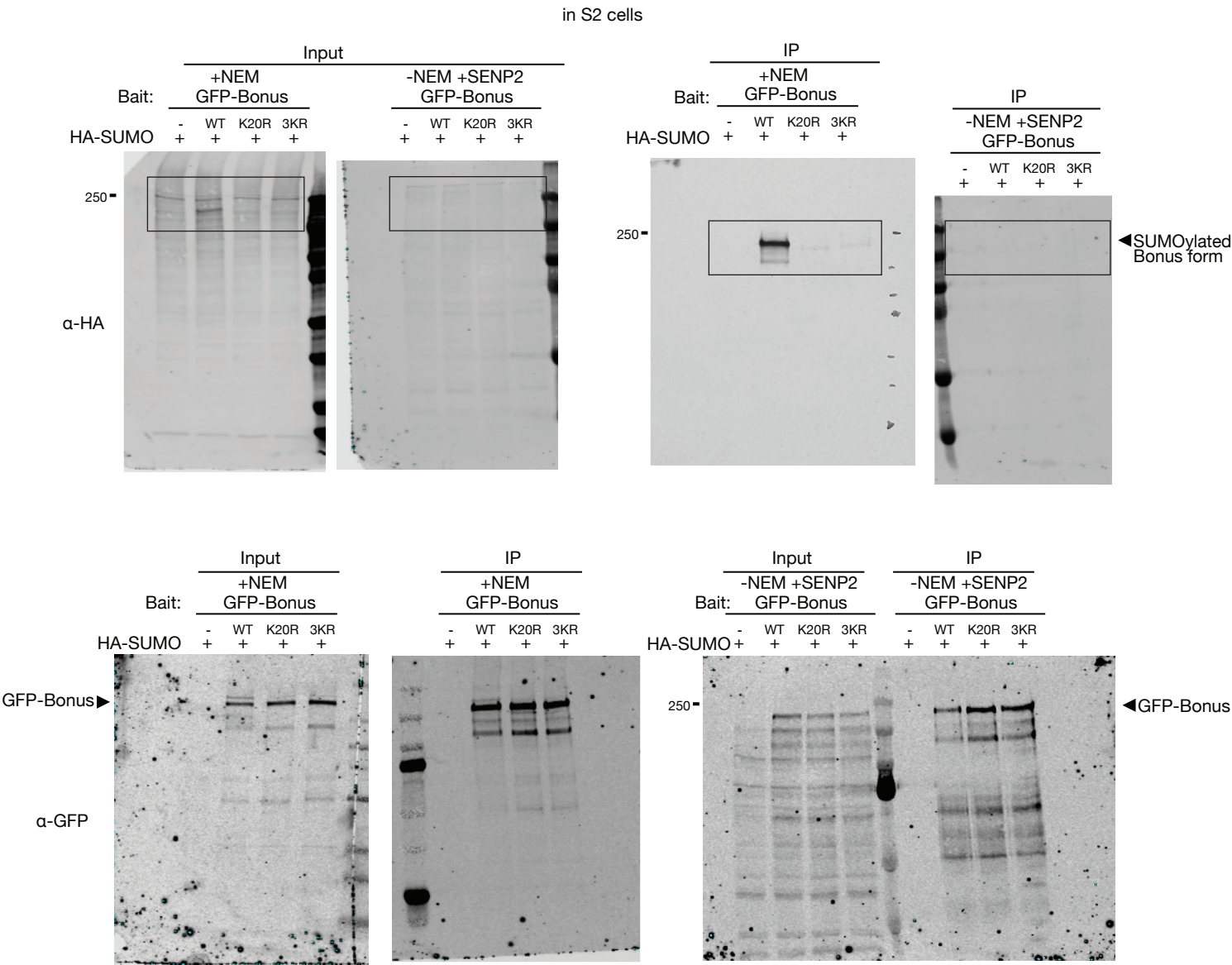

Supplement: Figure 5—figure supplement 1—source data 4. [file elife-89493-fig5-figsupp1-data4.zip › Figure 5 - figure supplement 1 - source data 4/Fig5-Figure suppl1D-uncropped blot.pdf]

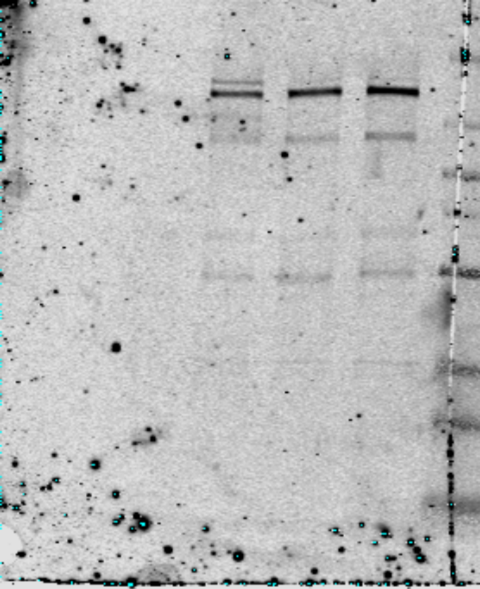

Supplement: Figure 5—figure supplement 1—source data 4. [file elife-89493-fig5-figsupp1-data4.zip › Figure 5 - figure supplement 1 - source data 4/Raw_image_Fig5-Fig-suppl1D-Input-Bonus+NEM.jpg]

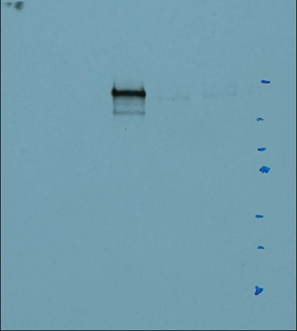

Supplement: Figure 5—figure supplement 1—source data 4. [file elife-89493-fig5-figsupp1-data4.zip › Figure 5 - figure supplement 1 - source data 4/Raw_image_Fig5-Fig-suppl1D-IP-SUMO+NEM.jpg]

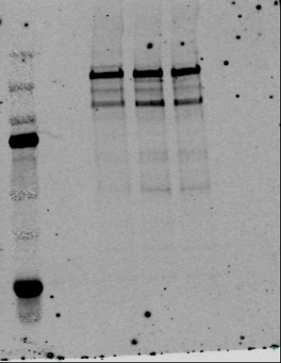

Supplement: Figure 5—figure supplement 1—source data 4. [file elife-89493-fig5-figsupp1-data4.zip › Figure 5 - figure supplement 1 - source data 4/Raw_image_Fig5-Fig-suppl1D-IP-Bonus+NEM.jpg]

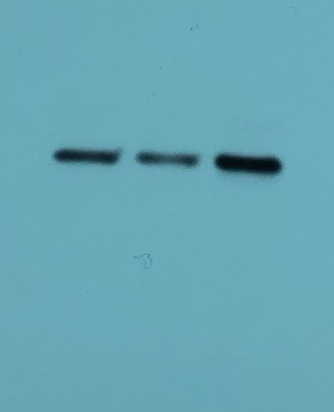

Supplement: Figure 5—figure supplement 2—source data 1. [file elife-89493-fig5-figsupp2-data1.zip › Figure 5 - figure supplement 2 - source data 1/Raw_image_Fig5-Fig-suppl2A-Input-Mi2-deltaN.jpg]

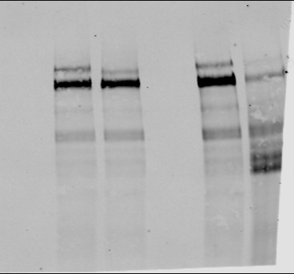

Supplement: Figure 5—figure supplement 2—source data 1. [file elife-89493-fig5-figsupp2-data1.zip › Figure 5 - figure supplement 2 - source data 1/Raw_image_Fig5-Fig-suppl2A-IP-Bonus.jpg]

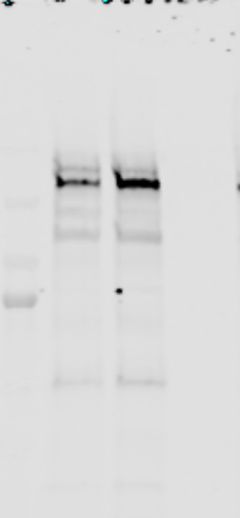

Supplement: Figure 5—figure supplement 2—source data 1. [file elife-89493-fig5-figsupp2-data1.zip › Figure 5 - figure supplement 2 - source data 1/Raw_image_Fig5-Fig-suppl2A-IP-Bonus-fordeltaC.jpg]

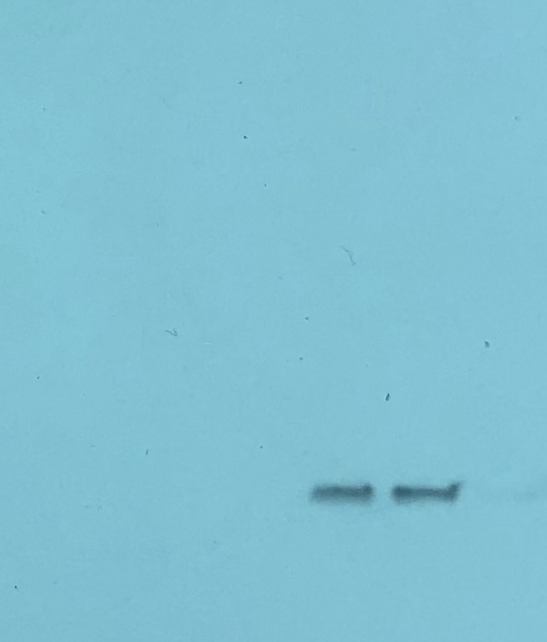

Supplement: Figure 5—figure supplement 2—source data 1. [file elife-89493-fig5-figsupp2-data1.zip › Figure 5 - figure supplement 2 - source data 1/Raw_image_Fig5-Fig-suppl2A-IP-Mi2-deltaC.jpg]

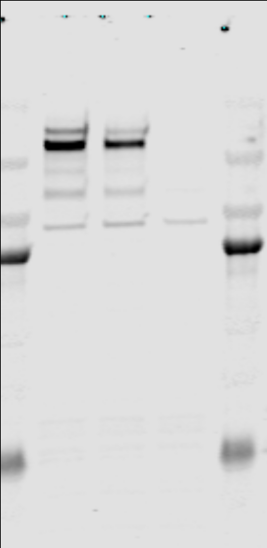

Supplement: Figure 5—figure supplement 2—source data 1. [file elife-89493-fig5-figsupp2-data1.zip › Figure 5 - figure supplement 2 - source data 1/Raw_image_Fig5-Fig-suppl2A-Inout-Bonus-fordeltaN.jpg]

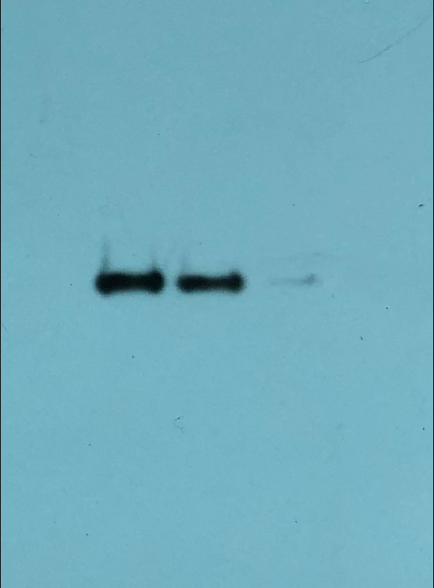

Supplement: Figure 5—figure supplement 2—source data 1. [file elife-89493-fig5-figsupp2-data1.zip › Figure 5 - figure supplement 2 - source data 1/Raw_image_Fig5-Fig-suppl2A-IP-Mi2-deltaN.jpg]

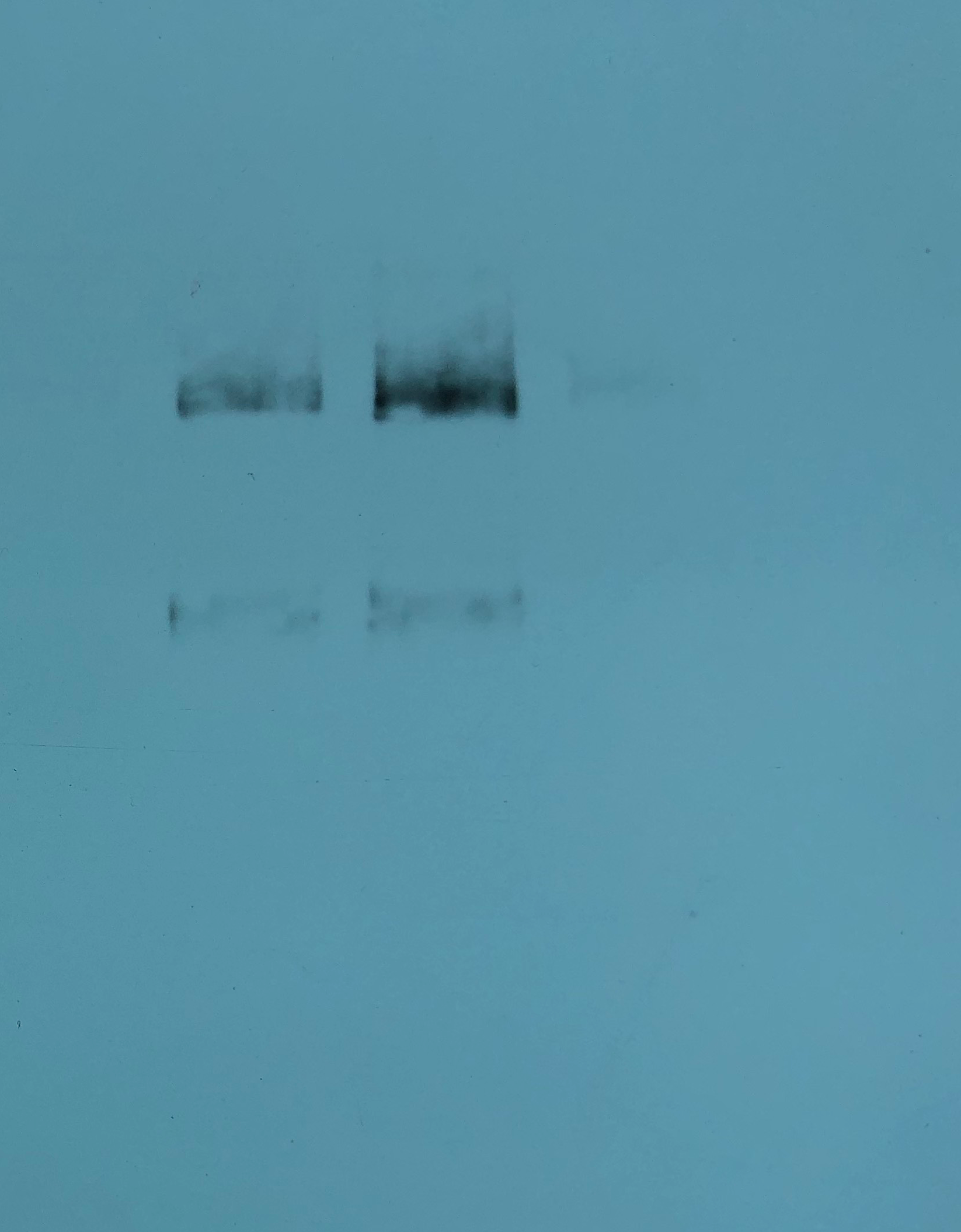

Supplement: Figure 5—figure supplement 2—source data 1. [file elife-89493-fig5-figsupp2-data1.zip › Figure 5 - figure supplement 2 - source data 1/Raw_image_Fig5-Fig-suppl2A-IP-Mi2.jpg]

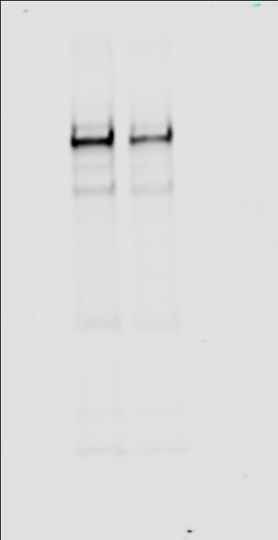

Supplement: Figure 5—figure supplement 2—source data 1. [file elife-89493-fig5-figsupp2-data1.zip › Figure 5 - figure supplement 2 - source data 1/Raw_image_Fig5-Fig-suppl2A-IP-Bonus-fordeltaN.jpg]

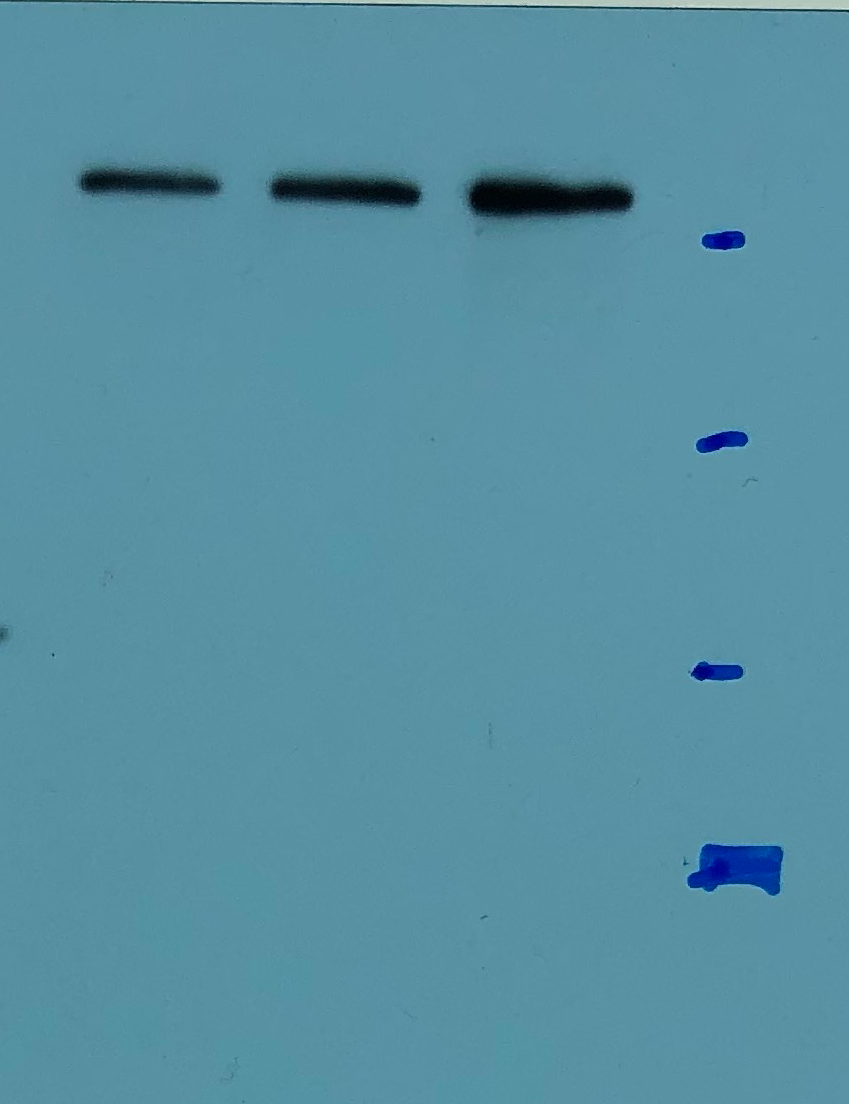

Supplement: Figure 5—figure supplement 2—source data 1. [file elife-89493-fig5-figsupp2-data1.zip › Figure 5 - figure supplement 2 - source data 1/Raw_image_Fig5-Fig-suppl2A-Input-Mi2.jpg]

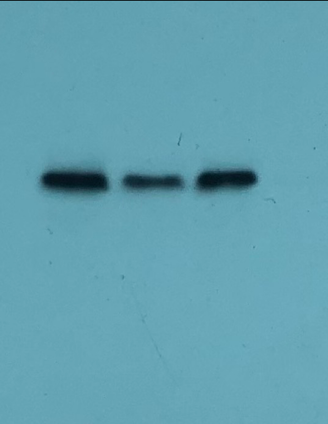

Supplement: Figure 5—figure supplement 2—source data 1. [file elife-89493-fig5-figsupp2-data1.zip › Figure 5 - figure supplement 2 - source data 1/Raw_image_Fig5-Fig-suppl2A-Input-Mi2-deltaC.jpg]

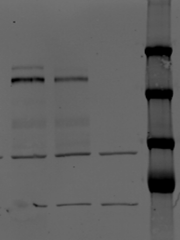

Supplement: Figure 5—figure supplement 2—source data 1. [file elife-89493-fig5-figsupp2-data1.zip › Figure 5 - figure supplement 2 - source data 1/Raw_image_Fig5-Fig-suppl2A-Input-Bonus.jpg]

Figure 5 - figure supplement 2A

in S2 cells

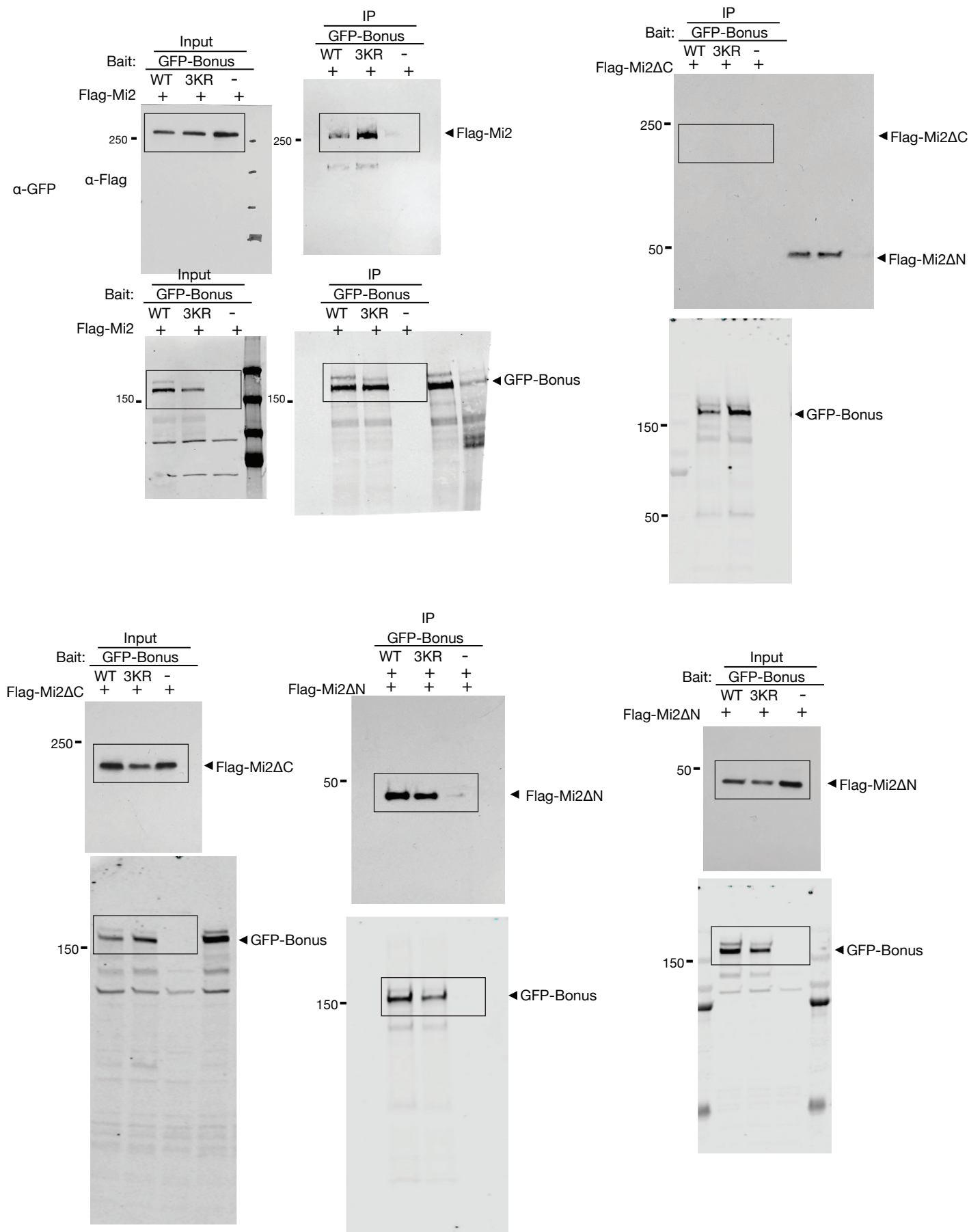

Supplement: Figure 5—figure supplement 2—source data 1. [file elife-89493-fig5-figsupp2-data1.zip › Figure 5 - figure supplement 2 - source data 1/Fig5-Figure suppl2A-uncropped blot.pdf]

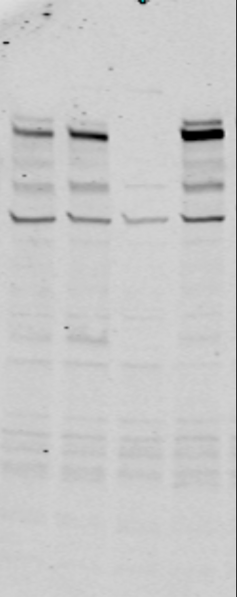

Supplement: Figure 5—figure supplement 2—source data 1. [file elife-89493-fig5-figsupp2-data1.zip › Figure 5 - figure supplement 2 - source data 1/Raw_image_Fig5-Fig-suppl2A-Input-Bonus-fordeltaC.jpg]

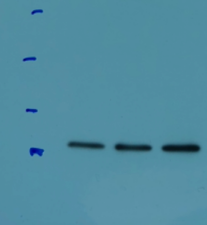

Supplement: Figure 5—figure supplement 2—source data 2. [file elife-89493-fig5-figsupp2-data2.zip › Figure 5 - figure supplement 2 - source data 2/Raw_image_Fig5-Fig-suppl2B-Input-Rpd3.jpg]

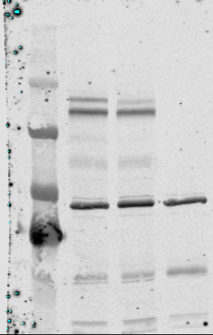

Supplement: Figure 5—figure supplement 2—source data 2. [file elife-89493-fig5-figsupp2-data2.zip › Figure 5 - figure supplement 2 - source data 2/Raw_image_Fig5-Fig-suppl2B-Input-Bonus.jpg]

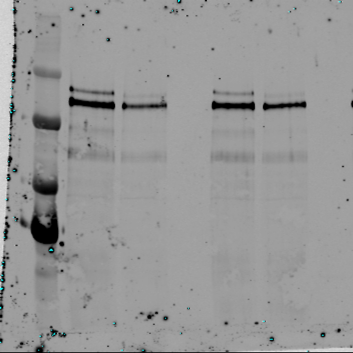

Supplement: Figure 5—figure supplement 2—source data 2. [file elife-89493-fig5-figsupp2-data2.zip › Figure 5 - figure supplement 2 - source data 2/Raw_image_Fig5-Fig-suppl2B-IP-Bonus.jpg]

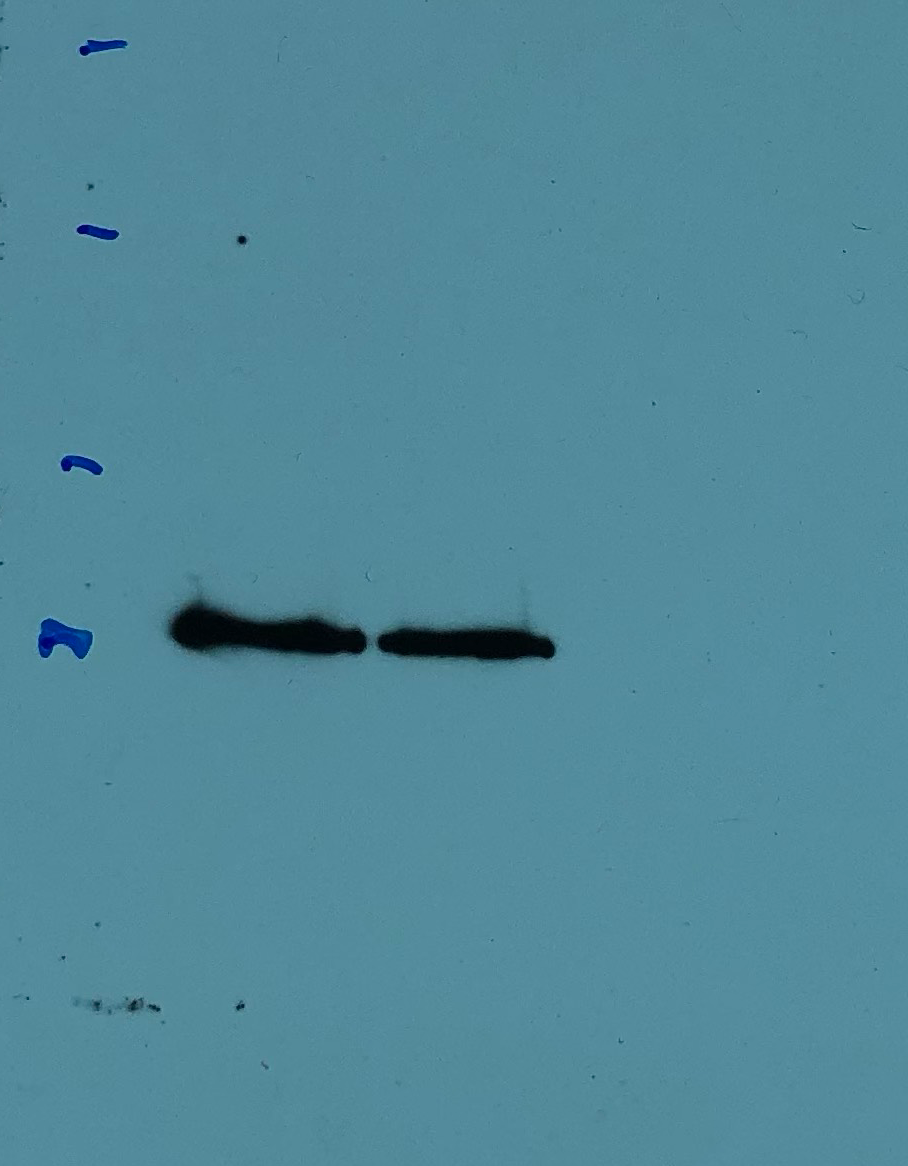

Supplement: Figure 5—figure supplement 2—source data 2. [file elife-89493-fig5-figsupp2-data2.zip › Figure 5 - figure supplement 2 - source data 2/Raw_image_Fig5-Fig-suppl2B-IP-Rpd3.jpg]

Figure 5 - figure supplement 2B

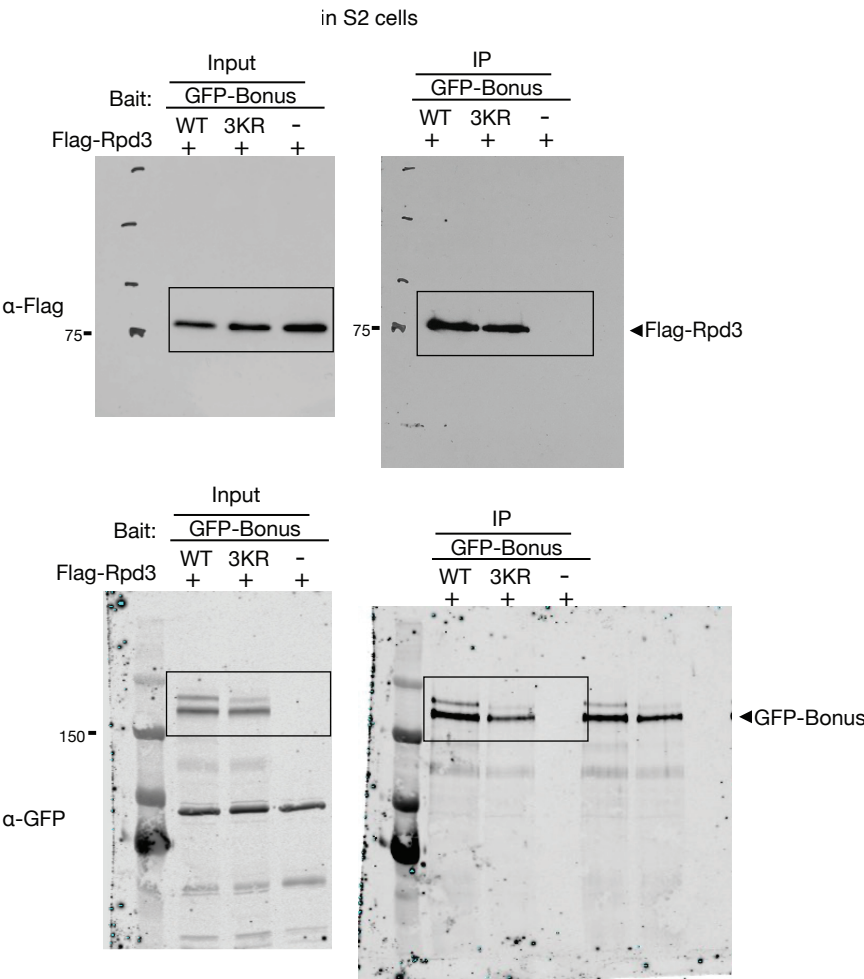

Supplement: Figure 5—figure supplement 2—source data 2. [file elife-89493-fig5-figsupp2-data2.zip › Figure 5 - figure supplement 2 - source data 2/Fig5-Figure suppl2B-uncropped blot.pdf]

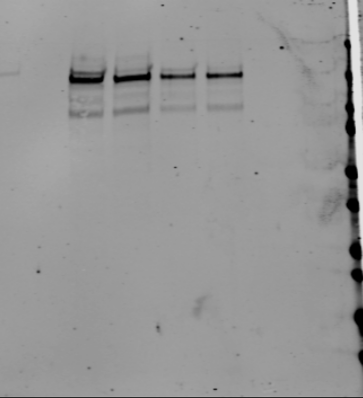

Supplement: Figure 5—figure supplement 2—source data 3. [file elife-89493-fig5-figsupp2-data3.zip › Figure 5 - figure supplement 2 - source data 3/Raw_image_Fig5-Fig-suppl2C-Input-Bonus.jpg]

Figure 5 - figure supplement 2C

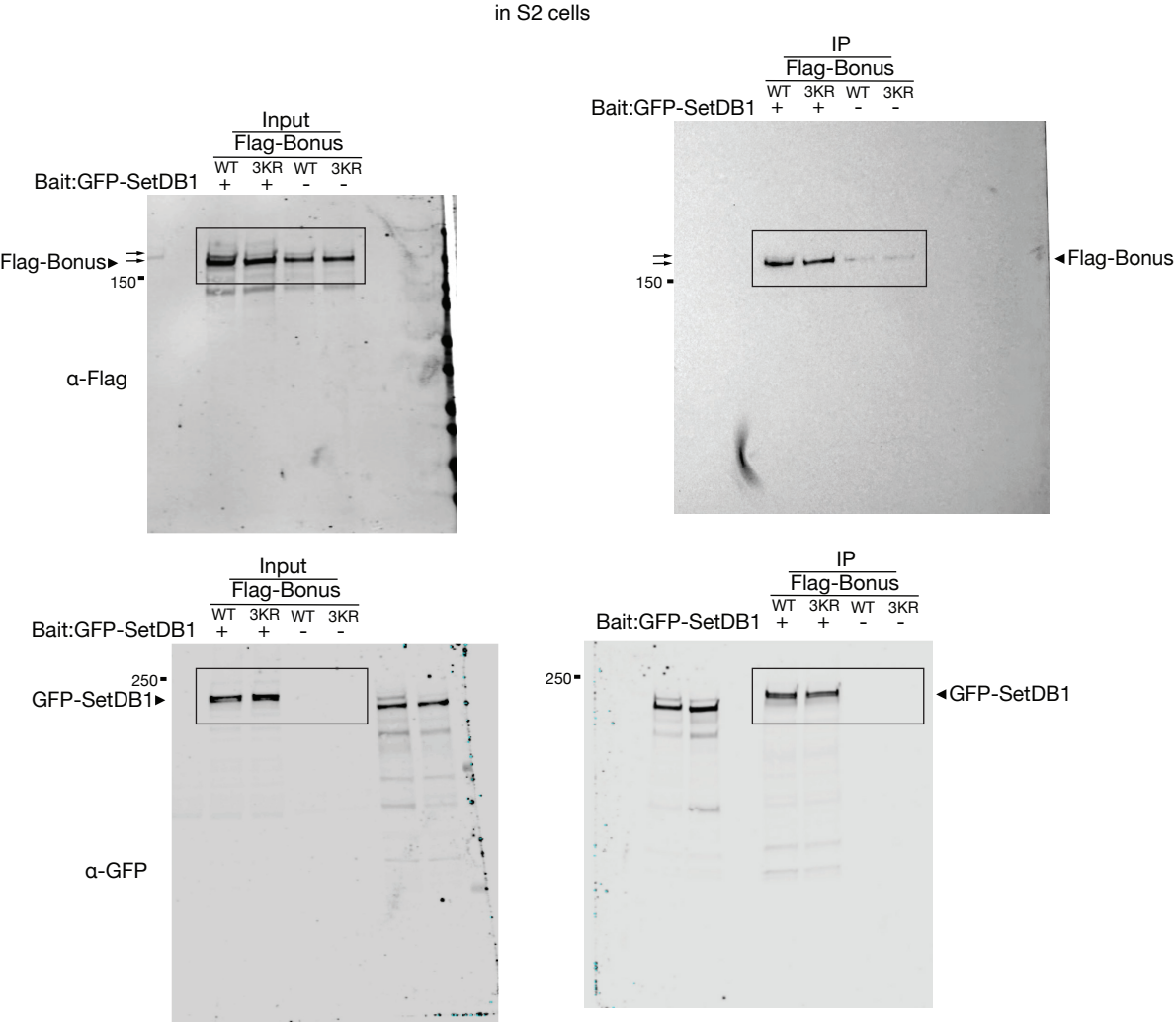

Supplement: Figure 5—figure supplement 2—source data 3. [file elife-89493-fig5-figsupp2-data3.zip › Figure 5 - figure supplement 2 - source data 3/Fig5-Figure suppl2C-uncropped blot.pdf]

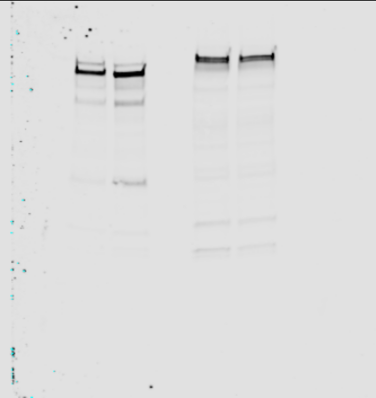

Supplement: Figure 5—figure supplement 2—source data 3. [file elife-89493-fig5-figsupp2-data3.zip › Figure 5 - figure supplement 2 - source data 3/Raw_image_Fig5-Fig-suppl2C-IP-SetDb1.jpg]

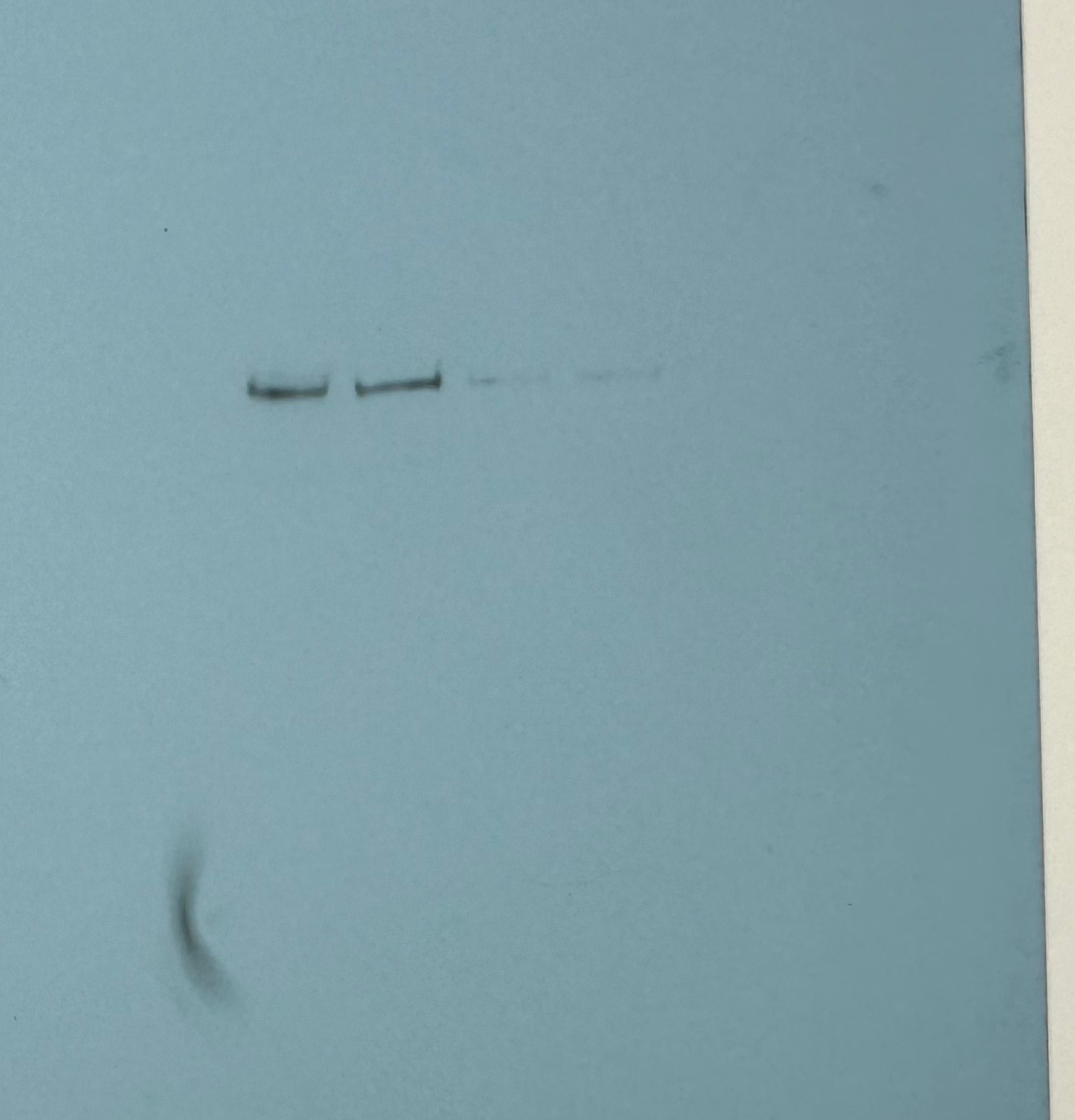

Supplement: Figure 5—figure supplement 2—source data 3. [file elife-89493-fig5-figsupp2-data3.zip › Figure 5 - figure supplement 2 - source data 3/Raw_image_Fig5-Fig-suppl2C-IP-Bonus.jpg]

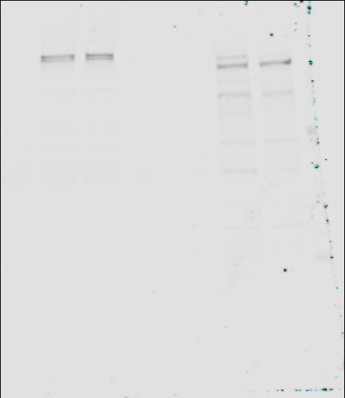

Supplement: Figure 5—figure supplement 2—source data 3. [file elife-89493-fig5-figsupp2-data3.zip › Figure 5 - figure supplement 2 - source data 3/Raw_image_Fig5-Fig-suppl2C-Input-SetDb1.jpg]

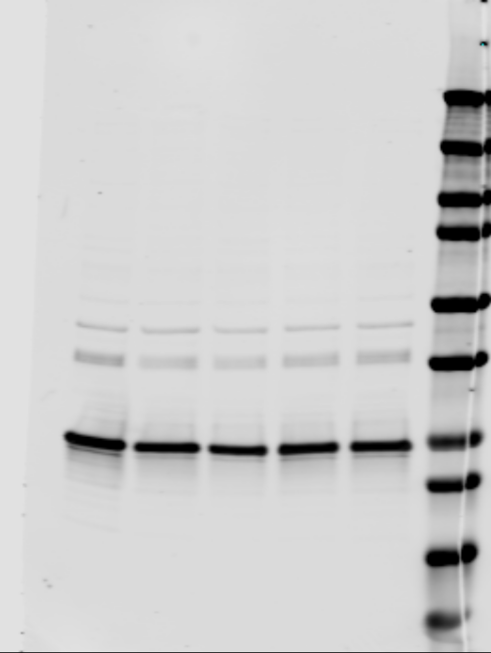

Supplement: Figure 6—source data 1. [file elife-89493-fig6-data1.zip › Figure 6 - source data 1/Raw_image_Figure6A-Input-Ubc9.jpg]

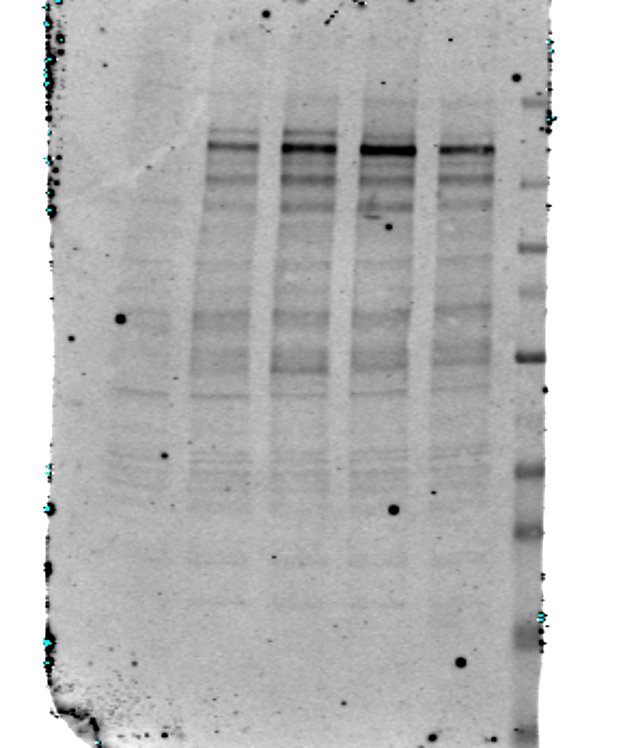

Supplement: Figure 6—source data 1. [file elife-89493-fig6-data1.zip › Figure 6 - source data 1/Raw_image_Figure6A-Input-Bonus.jpg]

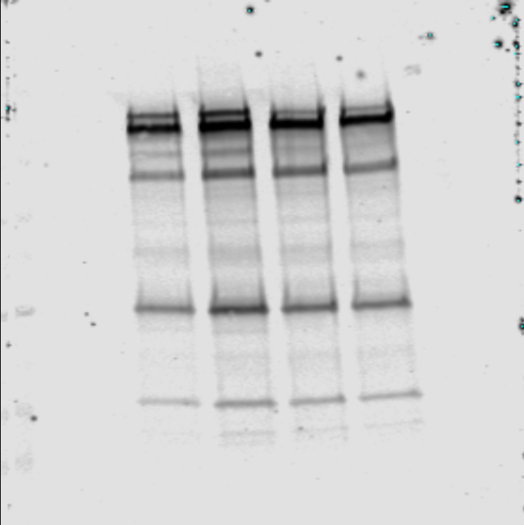

Supplement: Figure 6—source data 1. [file elife-89493-fig6-data1.zip › Figure 6 - source data 1/Raw_image_Figure6A-IP-Bonus.jpg]

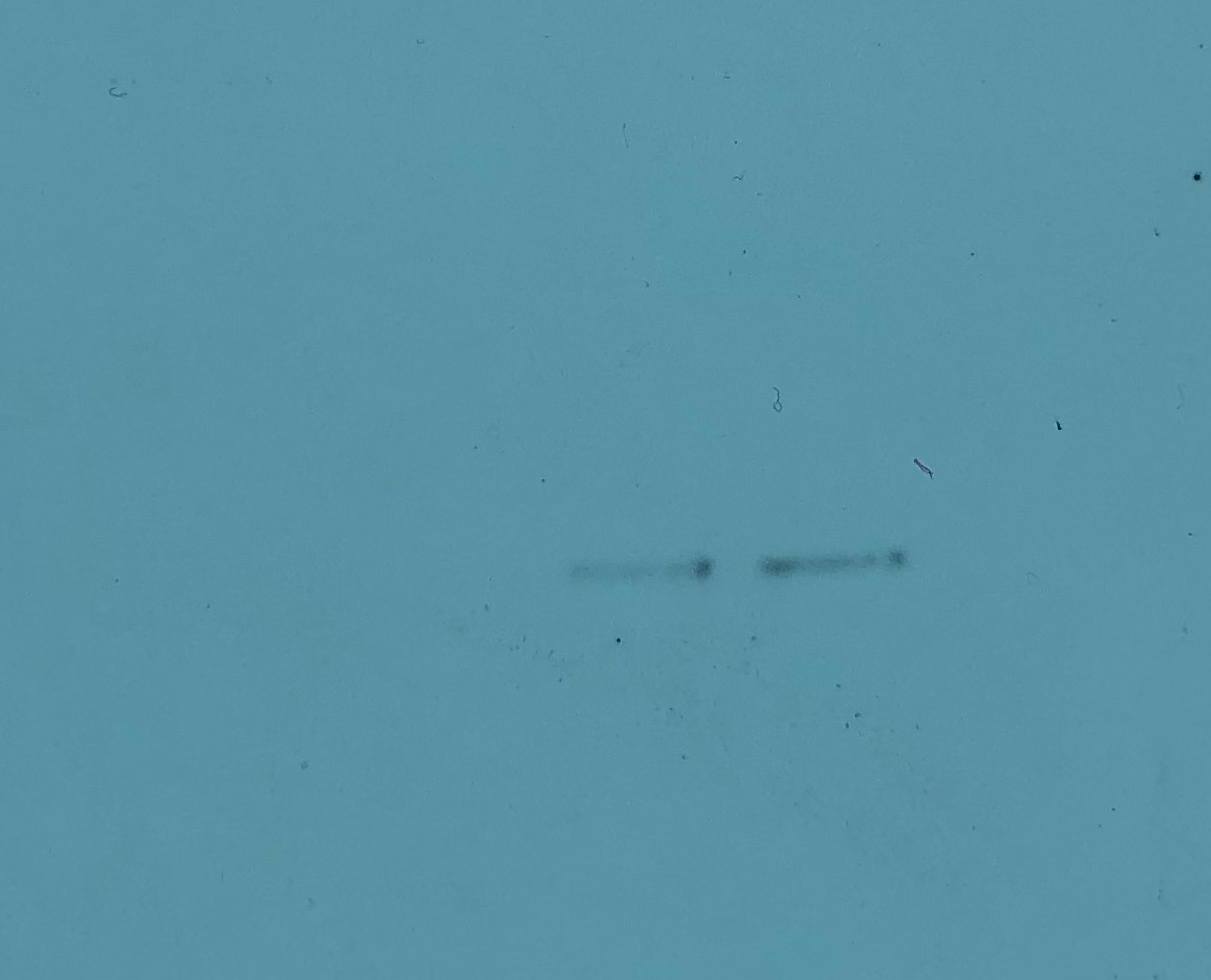

Supplement: Figure 6—source data 1. [file elife-89493-fig6-data1.zip › Figure 6 - source data 1/Raw_image_Figure6A-IP-Ubc9.jpg]

Figure 6A.

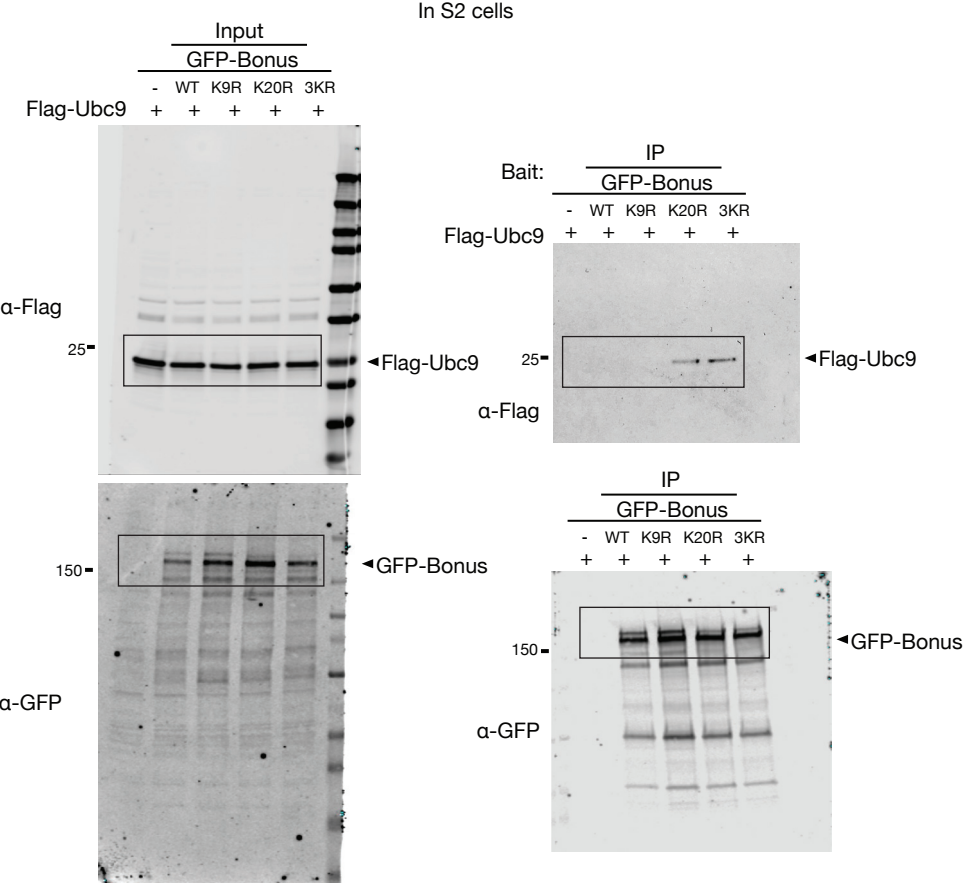

Supplement: Figure 6—source data 1. [file elife-89493-fig6-data1.zip › Figure 6 - source data 1/Figure6A-uncropped blot.pdf]

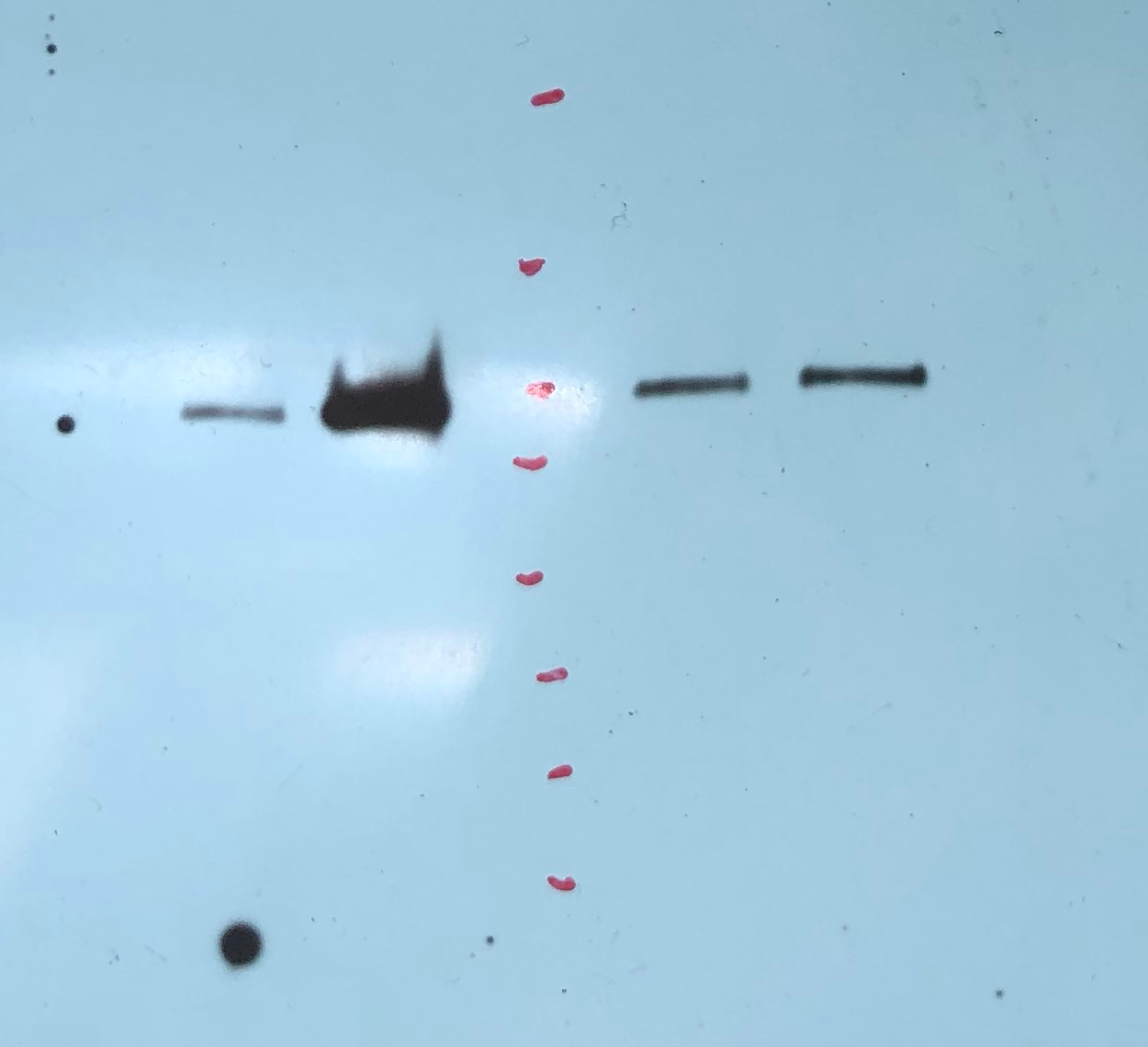

Supplement: Figure 6—source data 2. [file elife-89493-fig6-data2.zip › Figure 6 - source data 2/Raw_image_Figure6B-IP-Sv210.jpg]

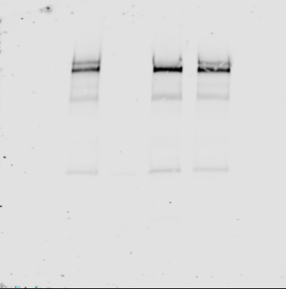

Supplement: Figure 6—source data 2. [file elife-89493-fig6-data2.zip › Figure 6 - source data 2/Raw_image_Figure6B-IP-Bonus.jpg]

Figure 6B.

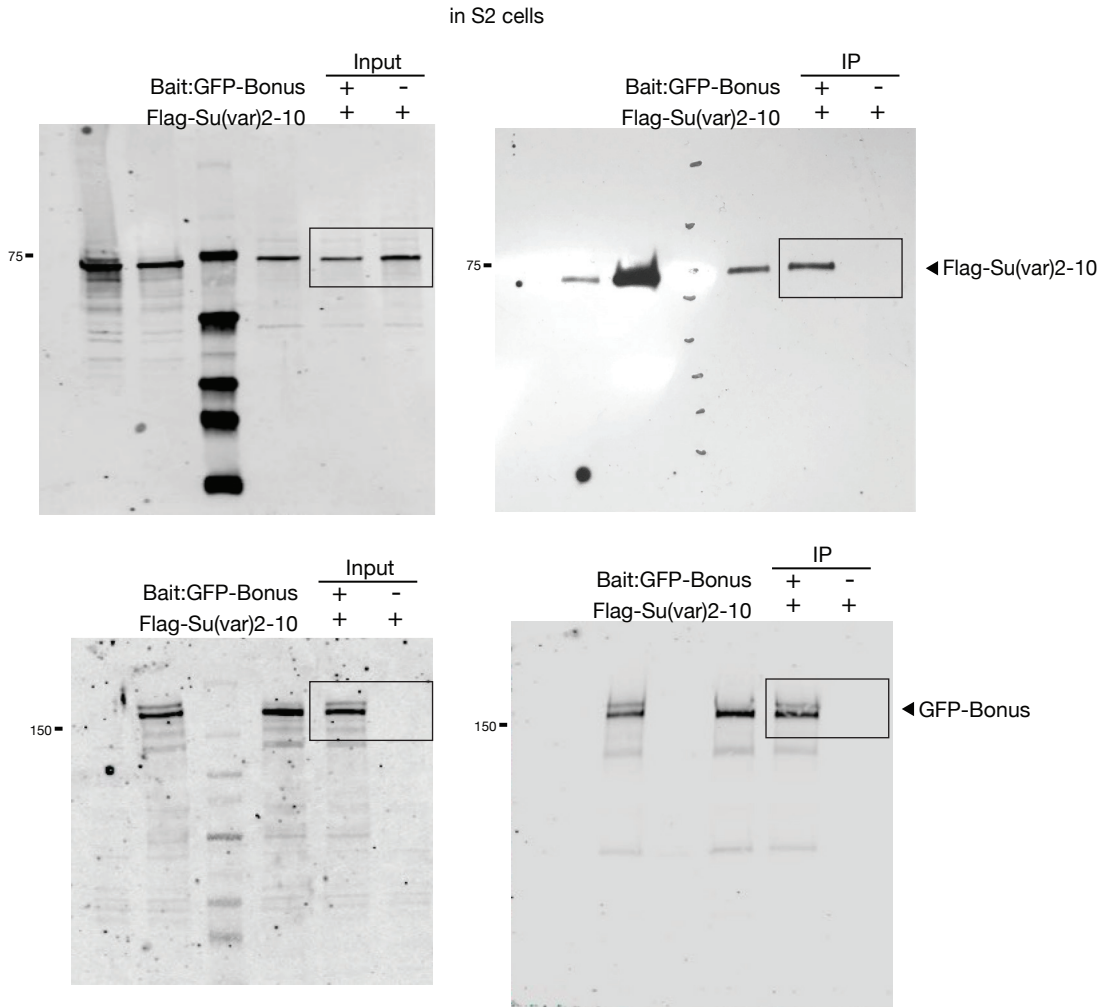

Supplement: Figure 6—source data 2. [file elife-89493-fig6-data2.zip › Figure 6 - source data 2/Figure6B-uncropped blot.pdf]

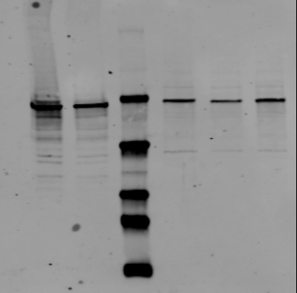

Supplement: Figure 6—source data 2. [file elife-89493-fig6-data2.zip › Figure 6 - source data 2/Raw_image_Figure6B-Input-Sv210.jpg]

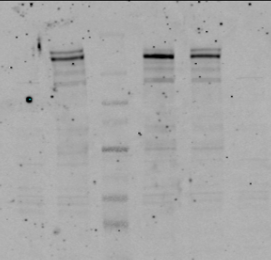

Supplement: Figure 6—source data 2. [file elife-89493-fig6-data2.zip › Figure 6 - source data 2/Raw_image_Figure6B-Input-Bonus.jpg]

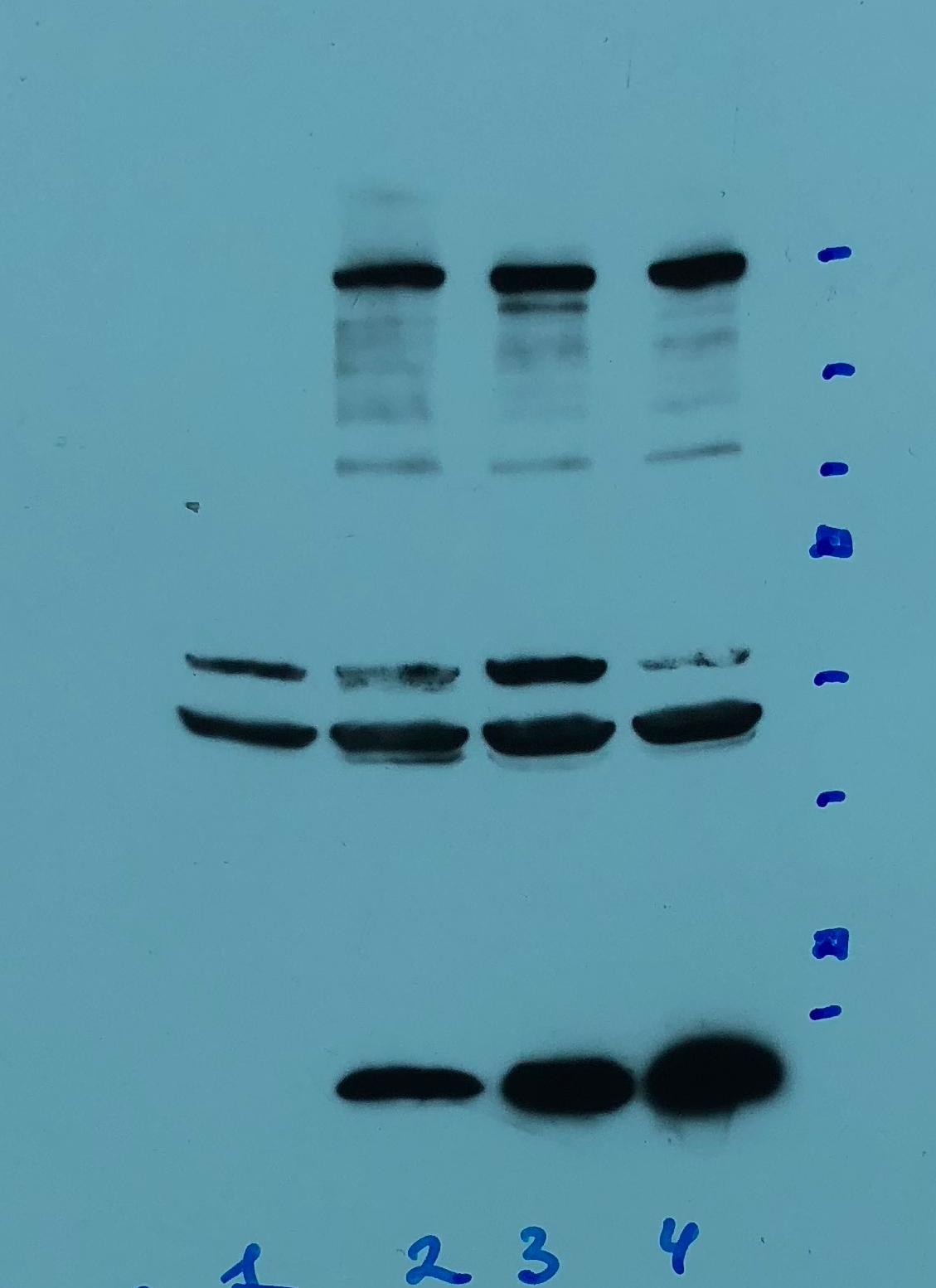

Supplement: Figure 6—source data 3. [file elife-89493-fig6-data3.zip › Figure 6 - source data 3/Raw_image_Figure6C-Input-SUMO.jpg]

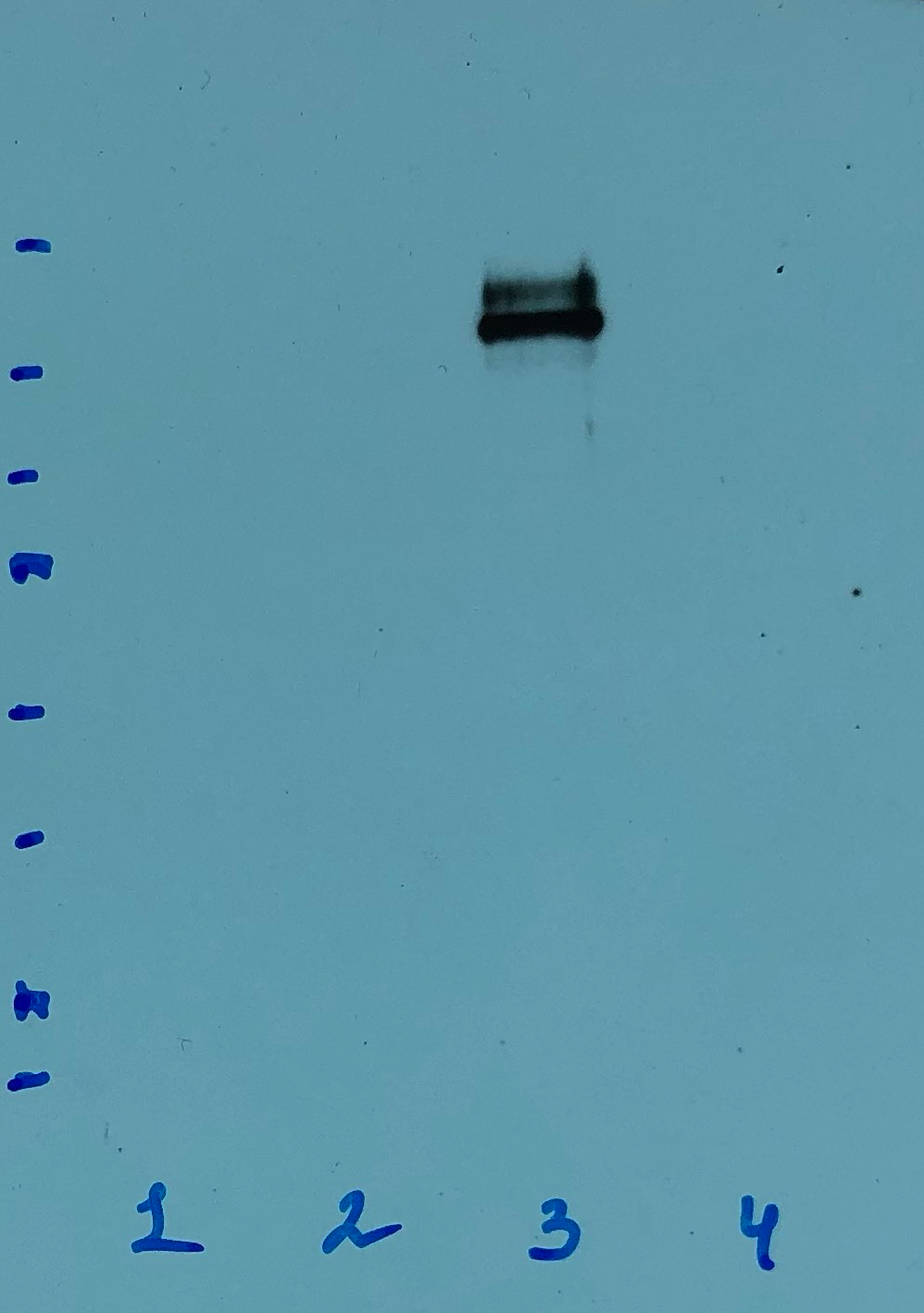

Supplement: Figure 6—source data 3. [file elife-89493-fig6-data3.zip › Figure 6 - source data 3/Raw_image_Figure6C-IP-SUMO.jpg]
