# Supplementary figures and images for: SUMOylation of Bonus, the Drosophila homolog of Transcription Intermediary Factor 1, safeguards germline identity by recruiting repressive chromatin complexes to silence tissue-specific genes (part 2 of 2)
Source: eLife. 2023 Nov 24;12:RP89493. doi: 10.7554/eLife.89493 (PMC10672805; doi:10.7554/eLife.89493)

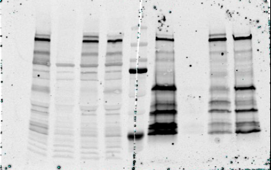

Supplement: Figure 6—source data 3. [file elife-89493-fig6-data3.zip › Figure 6 - source data 3/Raw_image_Figure6C-Bonus.jpg]

Figure 6C.

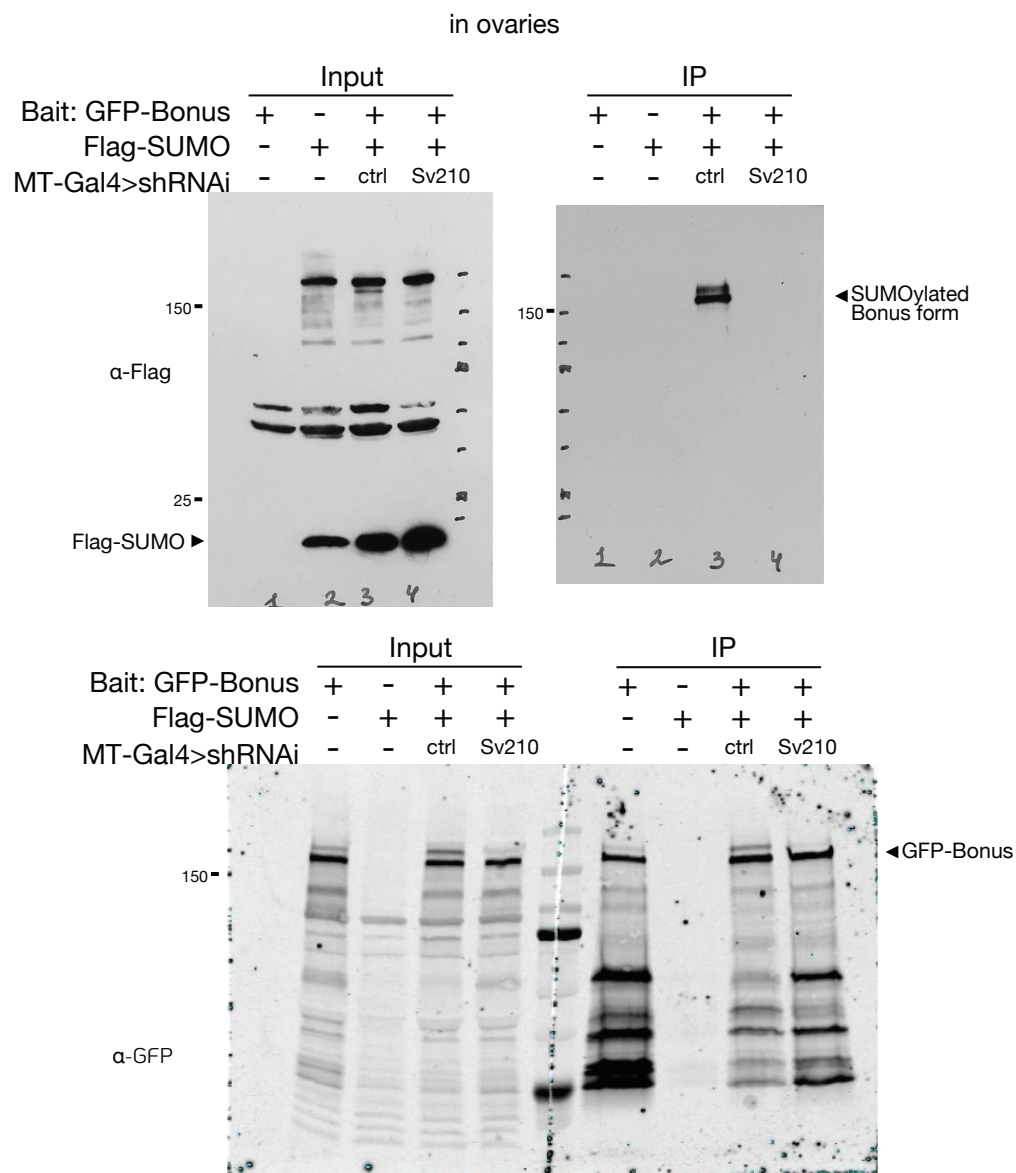

Supplement: Figure 6—source data 3. [file elife-89493-fig6-data3.zip › Figure 6 - source data 3/Figure6C-uncropped blot.pdf]

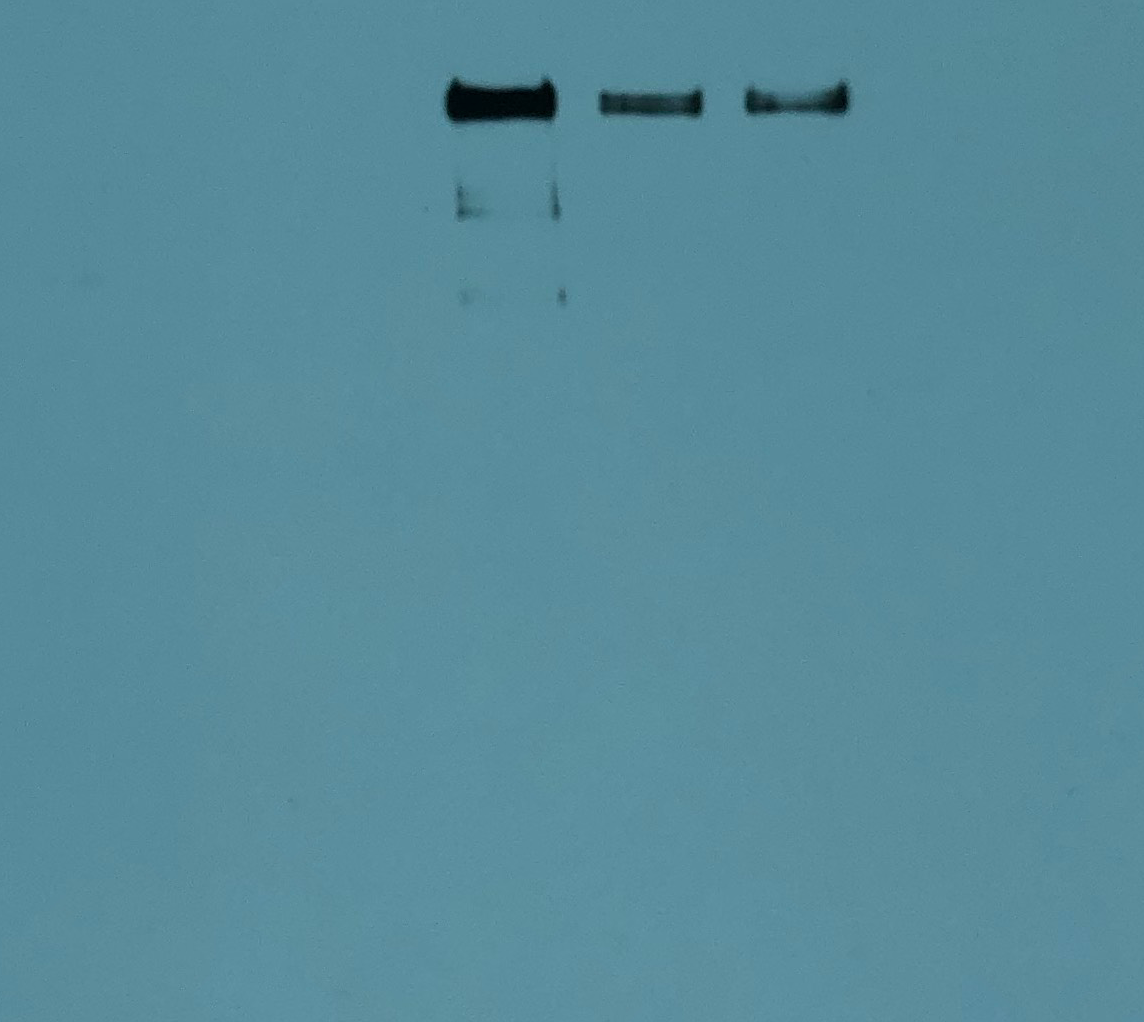

Supplement: Figure 6—figure supplement 1—source data 1. [file elife-89493-fig6-figsupp1-data1.zip › Figure 6 - figure supplement 1 - source data 1/Raw_image_Fig6-Fig-suppl1A-IP-Ubc9.jpg]

Figure 6 - figure supplement 1A

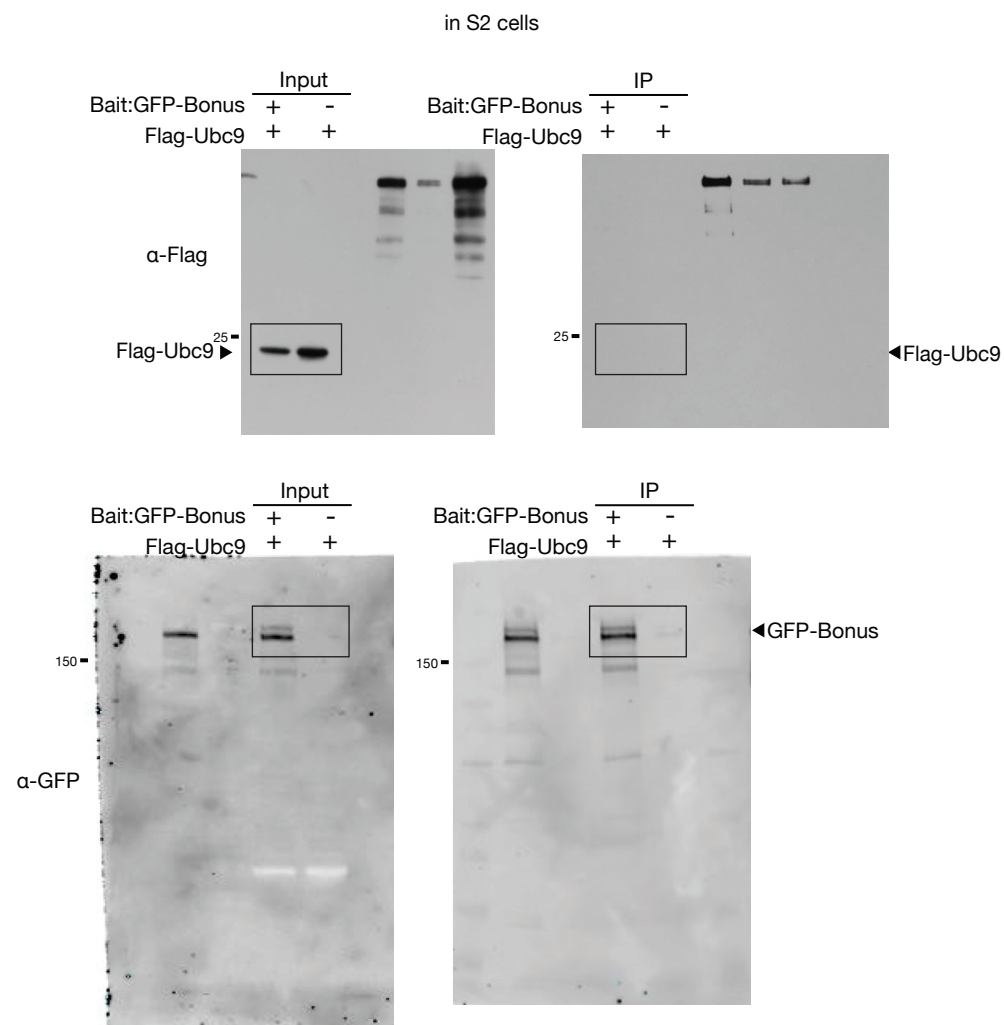

Supplement: Figure 6—figure supplement 1—source data 1. [file elife-89493-fig6-figsupp1-data1.zip › Figure 6 - figure supplement 1 - source data 1/Fig6-Figure suppl1A-uncropped blot.pdf]

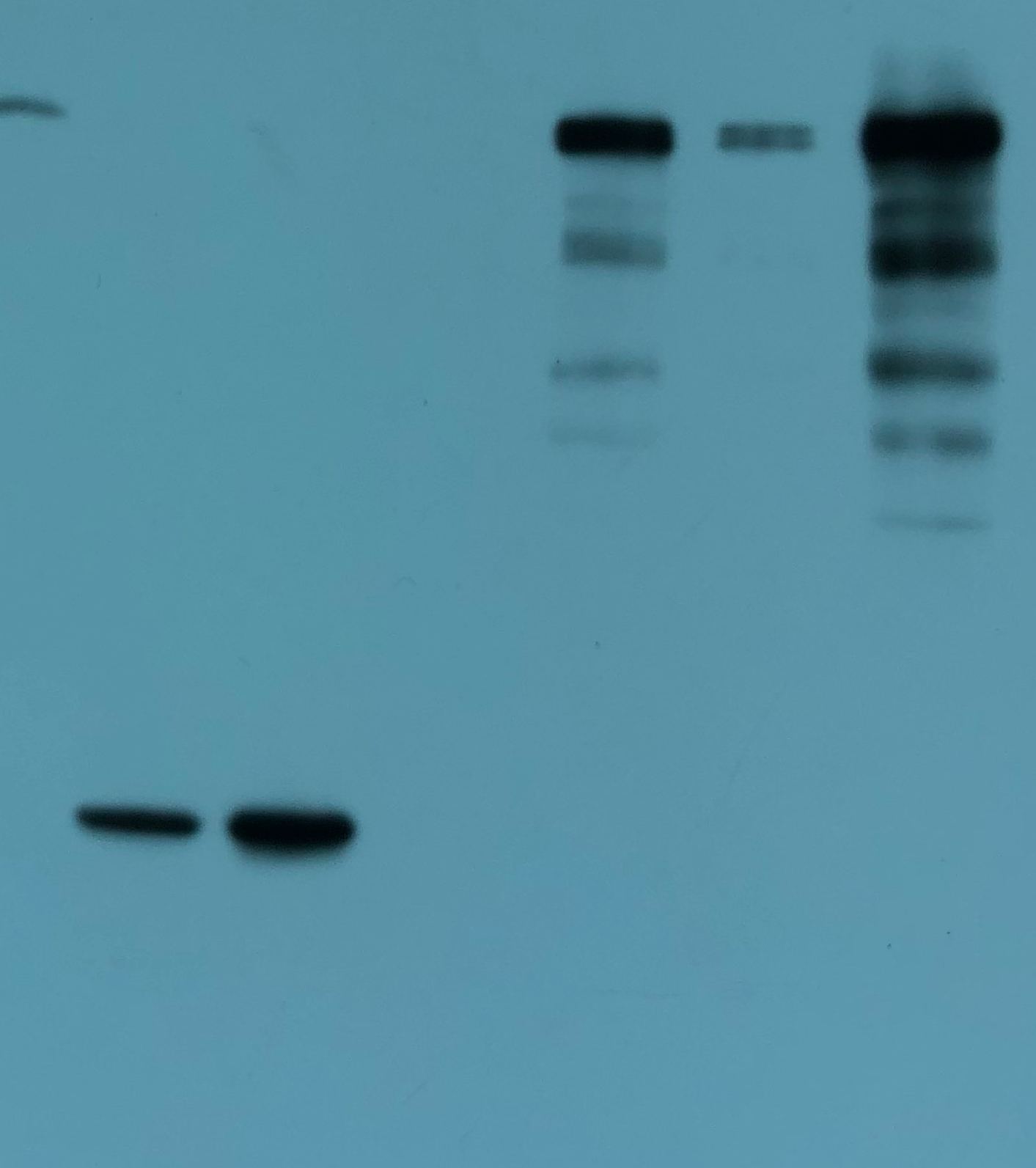

Supplement: Figure 6—figure supplement 1—source data 1. [file elife-89493-fig6-figsupp1-data1.zip › Figure 6 - figure supplement 1 - source data 1/Raw_image_Fig6-Fig-suppl1A-Input-Ubc9.jpg]

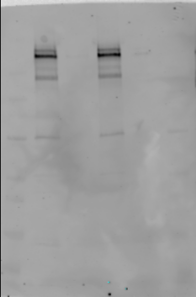

Supplement: Figure 6—figure supplement 1—source data 1. [file elife-89493-fig6-figsupp1-data1.zip › Figure 6 - figure supplement 1 - source data 1/Raw_image_Fig6-Fig-suppl1A-IP-Bonus.jpg]

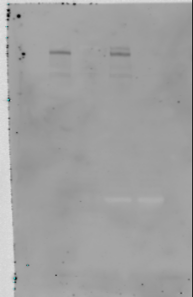

Supplement: Figure 6—figure supplement 1—source data 1. [file elife-89493-fig6-figsupp1-data1.zip › Figure 6 - figure supplement 1 - source data 1/Raw_image_Fig6-Fig-suppl1A-Input-Bonus.jpg]

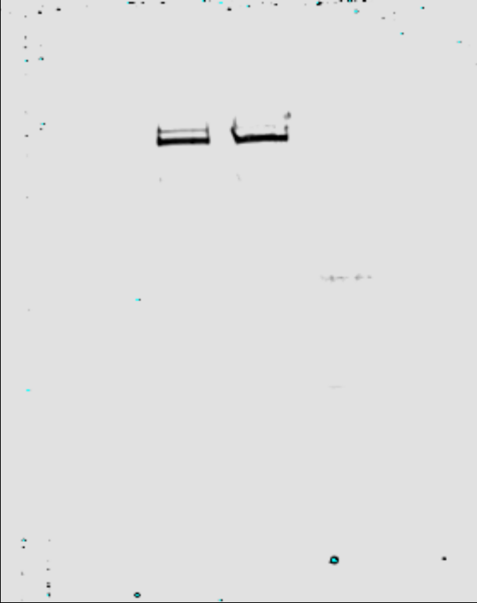

Supplement: Figure 6—figure supplement 1—source data 2. [file elife-89493-fig6-figsupp1-data2.zip › Figure 6 - figure supplement 1 - source data 2/Raw_image_Fig6-Fig-suppl1B-IP-Bonus.jpg]

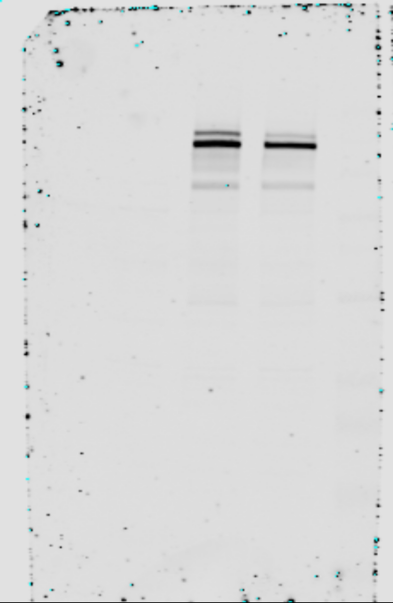

Supplement: Figure 6—figure supplement 1—source data 2. [file elife-89493-fig6-figsupp1-data2.zip › Figure 6 - figure supplement 1 - source data 2/Raw_image_Fig6-Fig-suppl1B-Input-Bonus.jpg]

Figure 6 - figure supplement 1B

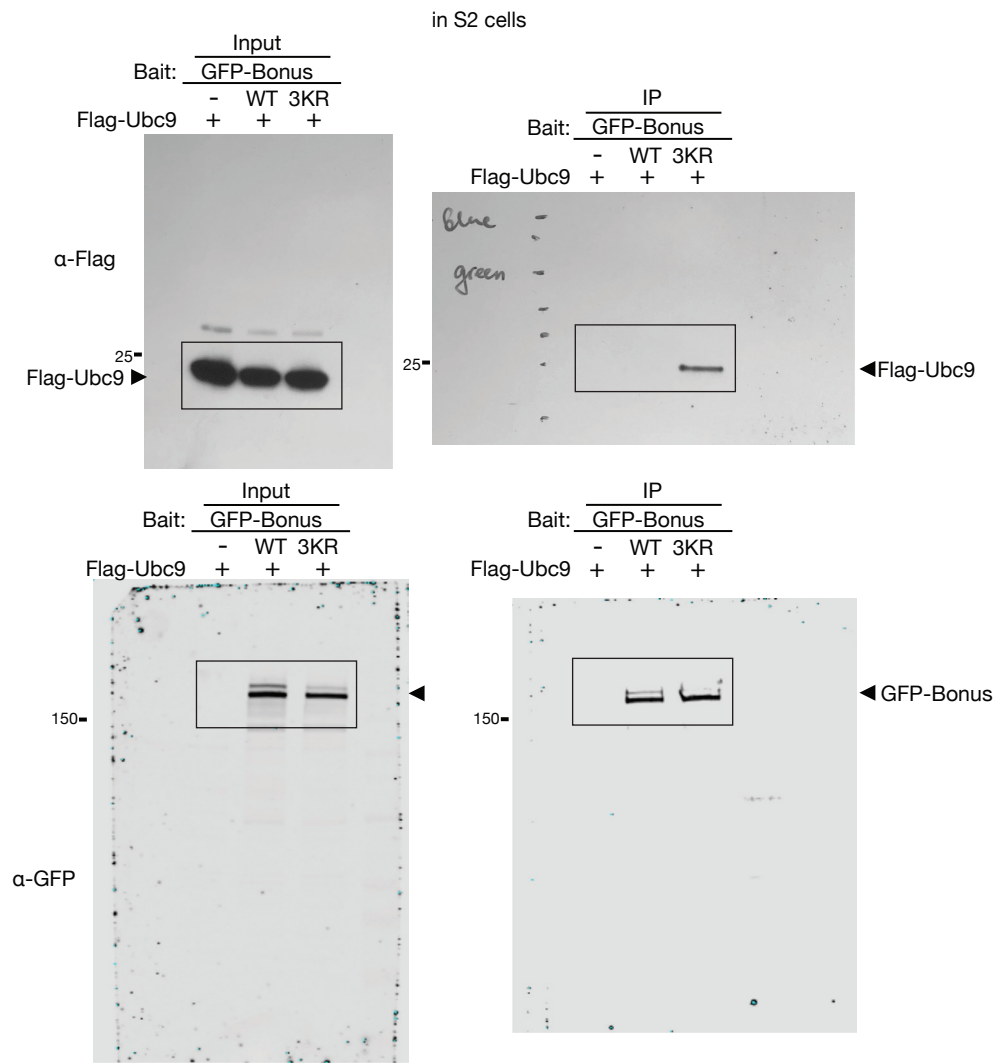

Supplement: Figure 6—figure supplement 1—source data 2. [file elife-89493-fig6-figsupp1-data2.zip › Figure 6 - figure supplement 1 - source data 2/Fig6-Figure suppl1B-uncropped blot.pdf]

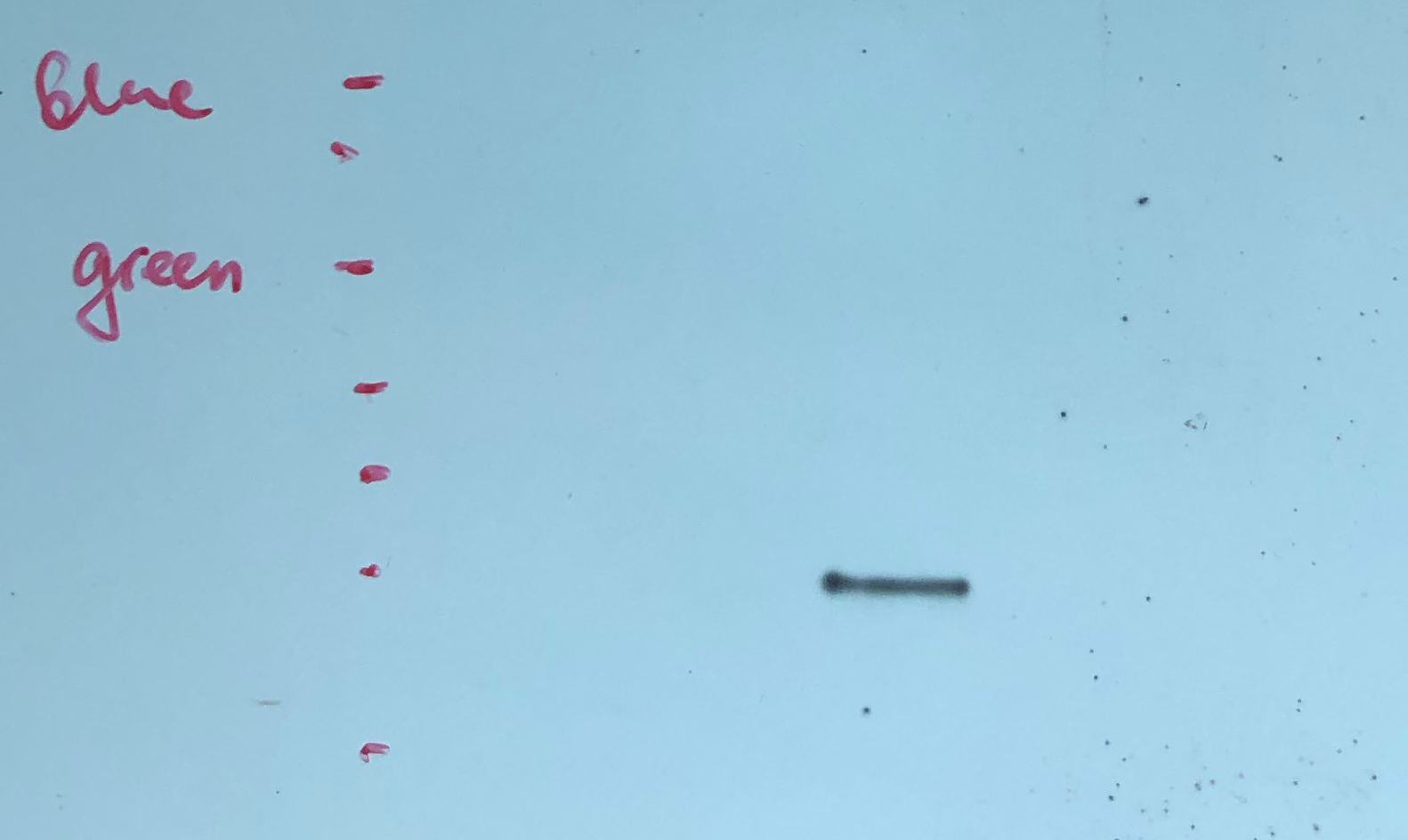

Supplement: Figure 6—figure supplement 1—source data 2. [file elife-89493-fig6-figsupp1-data2.zip › Figure 6 - figure supplement 1 - source data 2/Raw_image_Fig6-Fig-suppl1B-IP-Ubc9.jpg]

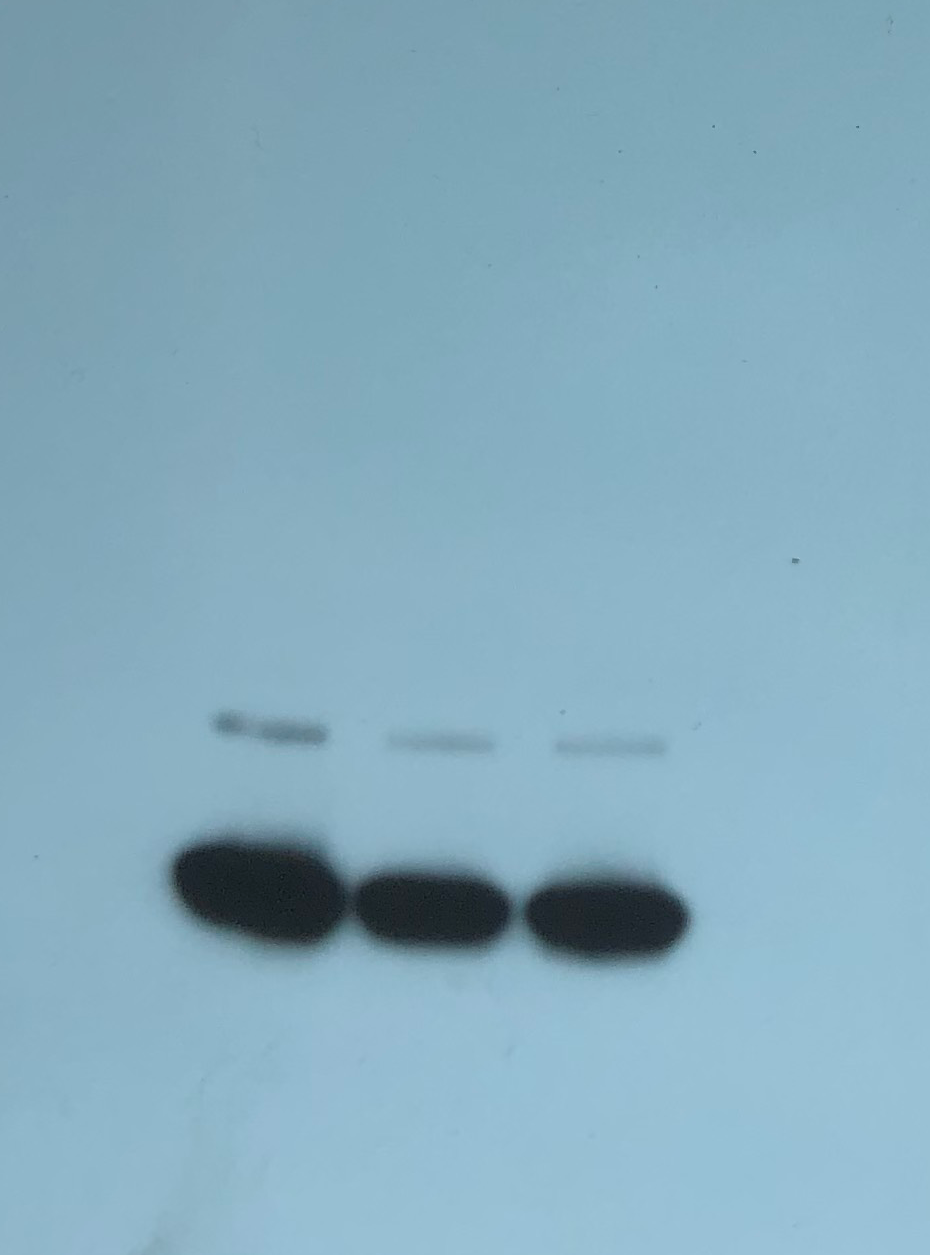

Supplement: Figure 6—figure supplement 1—source data 2. [file elife-89493-fig6-figsupp1-data2.zip › Figure 6 - figure supplement 1 - source data 2/Raw_image_Fig6-Fig-suppl1B-Input-Ubc9.jpg]
