# Supplementary material for: SUMOylation of Bonus, the Drosophila homolog of Transcription Intermediary Factor 1, safeguards germline identity by recruiting repressive chromatin complexes to silence tissue-specific genes
Source: eLife. 2023 Nov 24;12:RP89493. doi: 10.7554/eLife.89493 (PMC10672805; doi:10.7554/eLife.89493)
Supplement: Supplementary file 1. [file elife-89493-supp1.docx]

**Supplementary File 1.**

**Supplementary Table 1. Primers used for RT-qPCR and shRNAs.**

| Purpose | Name | Sequence (5’ to 3’) |
| --- | --- | --- |
| TRiP Short Hairpin | shBonus | CACACTCGTTTCCGAAATCAA |
| Oligos for RT-qPCR and ChIP-qPCR | bon-for | ACTTCTGGGTCTGACTGGCGAAG |
|  | bon-rev | TCAACGCACCACGACGTGG |
|  | rp49-for | CCGCTTCAAGGGACAGTATCTG |
|  | rp49-rev | ATCTCGCCGCAGTAAACGC |
|  | ple-for | AGCAGACCAAACAAACCGTCCTC |
|  | ple-rev | CACATCTTGCTCGGAGGGAAT |
|  | rbp6-for | CGCACCTCCCTATCTATCTATCT |
|  | rbp6-rev | GTGTTCTTTTTTGCATTTTCTGTGCTT |
|  | CG34353-for | TACCGTTTTTTGGCACCTACGC |
|  | CG34353-rev | GATGGTGCTAGAATCTTGCCC |
|  | pst-for | GCAGCAAGCTATTCGATCGCAG |
|  | pst-rev | CTGCCAATCCCACAGTGCCAC |
|  | mKate_reporter-for | TCAGAGGGGTGAACTTCCCA |
|  | mKate_reporter-rev | CTCCCAGCCGAGTGTTTTCT |
|  | luc_reporter-for | TGATTATGTCCGGCTACGTGAAT |
|  | luc_reporter-rev | GGTCCACGATGAAGAAGTGCTC |
|  | CG3191-for | CGAAGGAGAGGTGCAGATATTC |
|  | CG3191-rev | GCTGGAGGAACTTGATGTAGG |
|  | Spn88Eb-for | CGAGCGTGAGCAGGAAAT |
|  | Spn88Eb-rev | GACTTGTATATGGTGCGGTAGG |
